# Engineering a microbial biosynthesis platform for de novo production of tropane alkaloids

**DOI:** 10.1038/s41467-019-11588-w

**Published:** 2019-08-12

**Authors:** Prashanth Srinivasan, Christina D. Smolke

**Affiliations:** 1 Department of Bioengineering, Stanford University, Stanford, CA 94305 USA; 2 Chan Zuckerberg Biohub, San Francisco, CA 94158 USA

**Keywords:** Metabolic engineering, Natural product synthesis, Applied microbiology, Secondary metabolism

## Abstract

Tropane alkaloids (TAs) are a class of phytochemicals produced by plants of the nightshade family used for treating diverse neurological disorders. Here, we demonstrate de novo production of tropine, a key intermediate in the biosynthetic pathway of medicinal TAs such as scopolamine, from simple carbon and nitrogen sources in yeast (*Saccharomyces cerevisiae*). Our engineered strain incorporates 15 additional genes, including 11 derived from diverse plants and bacteria, and 7 disruptions to yeast regulatory or biosynthetic proteins to produce tropine at titers of 6 mg/L. We also demonstrate the utility of our engineered yeast platform for the discovery of TA derivatives by combining biosynthetic modules from distant plant lineages to achieve de novo production of cinnamoyltropine, a non-canonical TA. Our engineered strain constitutes a starting point for future optimization efforts towards realizing industrial fermentation of medicinal TAs and a platform for the synthesis of TA derivatives with enhanced bioactivities.

## Abstract (web summary)

Tropane alkaloids (TAs) are a group of phytochemicals that are used to treat neurological disorders. Here, the authors engineer baker’s yeast to produce tropine, a key intermediate in the biosynthetic pathway of TAs, and cinnamoyltropine, a non-canonical TA, from simple carbon and nitrogen sources.

## Introduction

Tropane alkaloids (TAs) are anticholinergic secondary metabolites produced by *Atropa*, *Duboisia*, and other genera of the nightshade family (*Solanaceae*). Several TAs, including atropine, hyoscyamine, and scopolamine, are classified as essential medicines by the World Health Organization for treatment of organophosphate and nerve agent poisoning, gastrointestinal spasms, cardiac arrhythmia, and symptoms of Parkinson’s disease^1,2^. TAs are sourced primarily via cultivation in Australia of *Duboisia* species, in which they accumulate to 0.2–4% by weight in plant tissue^3^. Geographical restriction and reliance on monocultures renders the TA supply vulnerable to pests, changes in land use, and climate. No total chemical syntheses for TAs are sufficiently economical for industrial use due to challenging stereochemistries^4^. Efforts to improve TA production in native species and engineer production via overexpression of rate-limiting enzymes in transgenic hairy root cultures have had limited success^5,6^. Limited tools for genetic manipulation of *Solanaceae*, long generation times, and challenges with adapting hairy root cultures to large-scale bioreactors makes engineering plant hosts cost- and labor-intensive.

Baker’s yeast (*Saccharomyces cerevisiae*) is an attractive platform for industrial production of plant natural products with smaller resource and time requirements than plant-based supply chains. In addition to genetic tractability, metabolic plasticity, and compatibility with large-scale cultivation in bioreactors, yeast provides many requisite endomembrane structures and subcellular compartments for reconstitution of plant pathways with multiple cytochrome P450 enzymes^7^. Yeast-based cellular factories have been described for terpenoids such as artemisinic acid^8^, phenylpropanoids such as resveratrol and naringenin^9–11^, benzylisoquinoline alkaloids such as hydrocodone and noscapine^12–14^, and monoterpene indole alkaloids such as strictosidine^15^.

**Fig. 1:**
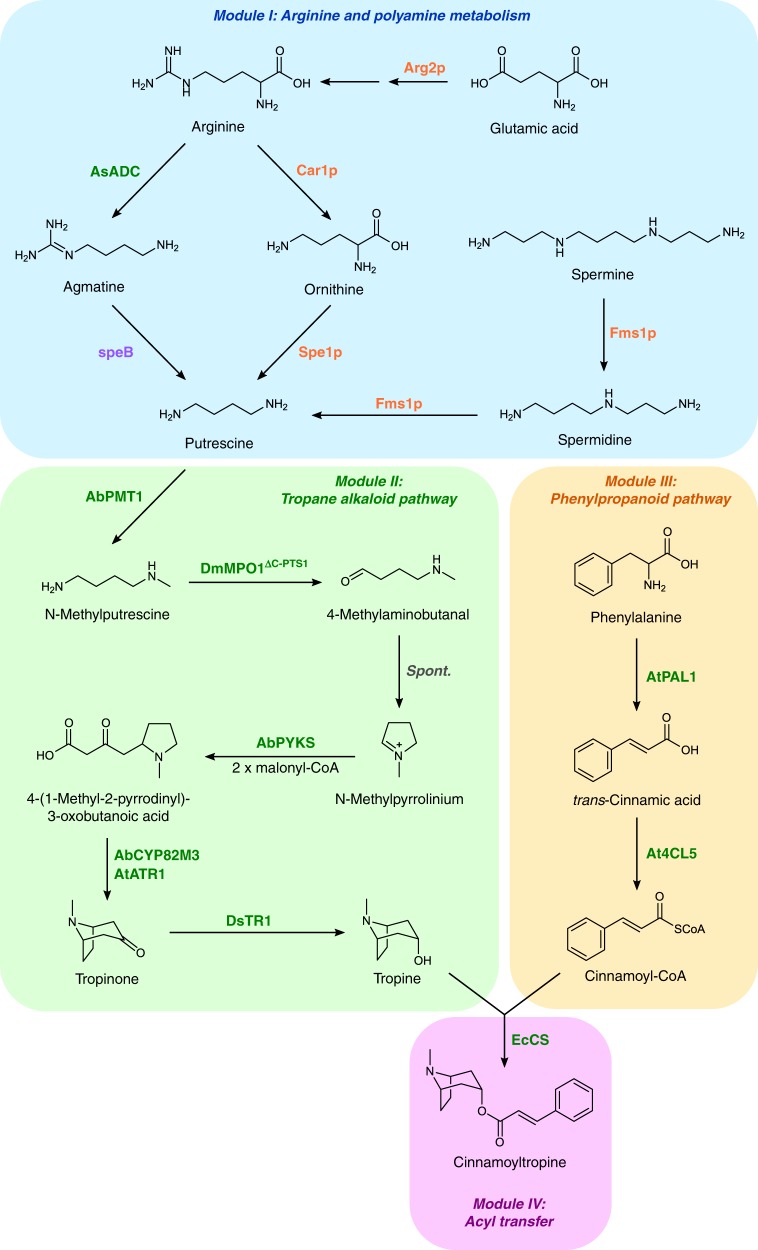
Engineered pathway for de novo biosynthesis of tropane alkaloids in yeast. Color scheme for enzyme labels: orange, overexpressed yeast enzymes; green, plant enzymes; violet, bacterial enzymes; grey, spontaneous (non-enzymatic) step. Enzyme symbols: Arg2p, glutamate *N-*acetyltransferase; Car1p, arginase; AsADC, *Avena sativa* arginine decarboxylase; speB, agmatine ureohydrolase; Spe1p, ornithine decarboxylase; Fms1p, polyamine oxidase; AbPMT1, *Atropa belladonna* putrescine *N-*methyltransferase 1; DmMPO1^ΔC-PTS1^, *Datura metel N-*methylputrescine oxidase 1 with peroxisome targeting sequence 1 and truncated C-terminus; AbPYKS, *A. belladonna* pyrrolidine ketide synthase; AbCYP82M3, *A. belladonna* tropinone synthase; AtATR1, *Arabidopsis thaliana* NADP^+^-cytochrome P450 reductase; DsTR1, *Datura stramonium* tropinone reductase I; AtPAL1, *A. thaliana* phenylalanine ammonia-lyase; At4CL5, *A. thaliana* 4-coumarate-CoA ligase 5; EcCS, *Erythroxylum coca* cocaine synthase

One challenge preventing a microbial production platform for TAs has been the lack of a fully characterized biosynthetic pathway. Medicinally relevant TAs, such as hyoscyamine and scopolamine, consist of an arginine-derived 8-azabicyclo[3.2.1]octane (tropine) moiety esterified with a phenylalanine-derived phenyllactate group by an unknown acyltransferase mechanism. The product, littorine, undergoes an internal free radical-mediated rearrangement catalyzed by a cytochrome P450 (CYP80F1) to produce hyoscyamine aldehyde^16,17^, which is reduced to hyoscyamine by an unidentified dehydrogenase or reductase. Finally, a bifunctional 2-oxoglutarate/Fe(II)-dependent hyoscyamine 6β-hydroxylase/oxygenase (H6H) catalyzes the conversion of hyoscyamine to scopolamine. Although the biosynthetic steps from arginine to the intermediate *N-*methylpyrrolinium (NMPy) were established over decades of work, the enzymes responsible for converting NMPy to tropine remained elusive (Fig. 1)^18^. Recently, researchers reported the discovery of an unusual type III polyketide synthase (PKS) from *Atropa belladonna*, which condenses two malonyl-CoAs with NMPy in the absence of an additional starter unit to form 4-(1-methyl-2-pyrrolidinyl)-3-oxobutanoic acid (MPOB), and a cytochrome P450, which catalyzes the oxidation and cyclization of MPOB to tropinone^19^, thereby completing the biosynthetic pathway between arginine and tropine (Fig. 1).

Although TA biosynthetic genes have been expressed in *Escherichia coli* and yeast for enzyme characterization^16,17^ or production of TAs from fed precursors^20–22^, no microbial platforms have been engineered to produce medicinal TAs from central metabolites. Very recently, the production of tropine and pseudotropine in engineered *S. cerevisiae* was reported^23^. However, limited optimization of precursor production in that study may present challenges in directing sufficient flux through the pathway to enable production of downstream TAs.

Here, we engineer a yeast platform for production of tropine from simple sugars. Our final strain combines four overexpressed yeast enzymes, seven disruptions to native regulatory proteins and biosynthetic enzymes, and nine heterologous enzymes derived from diverse plants and bacteria to produce tropine at titers of 5.9 mg/L (Fig. 1). We improve putrescine production by over 70-fold via overexpression of genes involved in arginine metabolism, disruption of polyamine regulatory mechanisms, and reconstitution of an alternate putrescine biosynthetic pathway. We optimize activity of a diamine oxidase by identifying orthologs from transcriptome data and eliminating a competing side reaction, resulting in a 68% improvement in NMPy production. De novo tropine production is achieved through expression of the recently discovered PKS and cytochrome P450, and optimization efforts to fine-tune expression stability of the P450 enzyme, alleviate auxotrophy, mitigate flux bottlenecks, and optimize media result in a 28-fold increase in tropine titers. We demonstrate the utility of the platform for producing non-natural and non-canonical TAs by implementing a metabolic module for phenylpropanoid biosynthesis and an acyltransferase from a distant TA-producing plant lineage to achieve de novo biosynthesis of cinnamoyltropine, a tropane ester not conventionally found in medicinal TA-producing plants (Fig. 1). Our work provides a platform for further engineering efforts directed towards optimizing TA production for industrially relevant titers and characterizing novel TA derivatives.

## Results

### Engineering a platform strain for putrescine overproduction

**Fig. 2:**
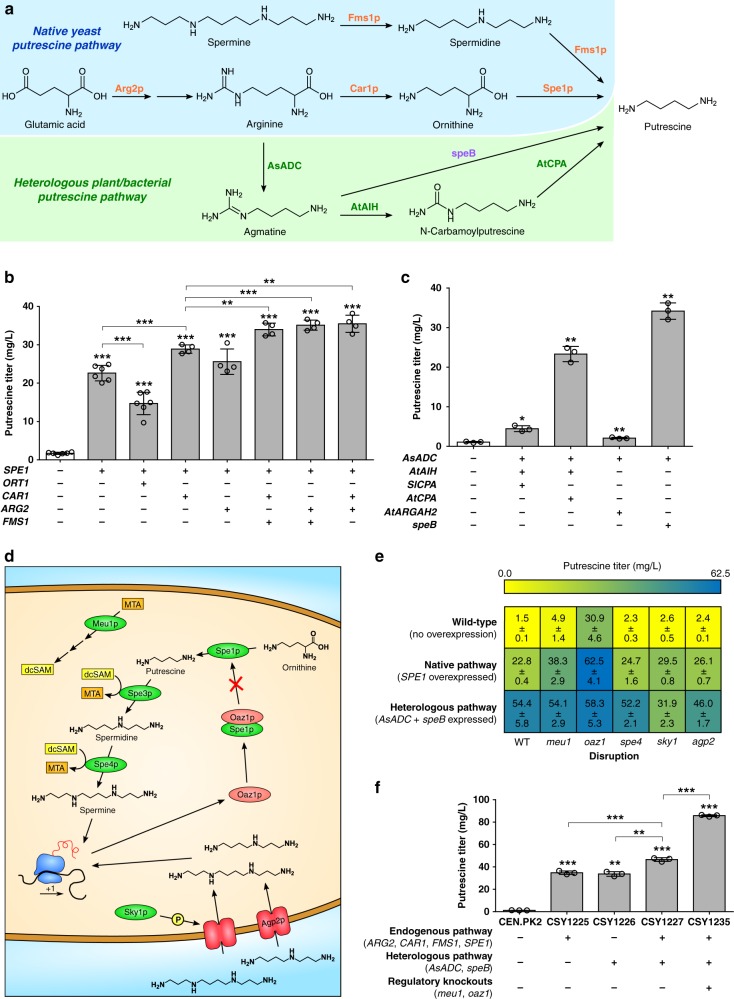
Engineering yeast for improved production of putrescine. **a** Endogenous yeast and heterologous biosynthetic pathways for production of putrescine from central metabolites. Color scheme for enzyme labels: orange, overexpressed yeast enzymes; green, plant enzymes; violet, bacterial enzymes. **b** Putrescine production in yeast strains engineered for overexpression of endogenous enzymes in arginine and polyamine metabolism. Additional copies of native genes were expressed from low-copy plasmids in wild-type yeast (CEN.PK2). **c** Putrescine production in yeast strains engineered to express a heterologous biosynthetic pathway from plants and bacteria. Heterologous enzymes were expressed from low-copy plasmids in wild-type yeast. **d** Illustration of the endogenous regulatory pathways that tightly control intracellular putrescine levels during normal yeast growth. **e** Heat map of putrescine production in yeast strains with disruptions to endogenous polyamine biosynthesis regulatory mechanisms. For overexpression of native or heterologous putrescine pathways, indicated genes were expressed from low-copy plasmids in wild-type yeast (WT) or each single disruption strain. **f** Summary of engineering efforts for optimizing putrescine production in yeast. Plus symbol indicates expression of at least one gene from the pathway, whereas minus indicates expression of no genes from the pathway. For all metabolite quantification, strains were cultured in selective (YNB-DO) media with 2% dextrose at 30 °C for 48 h (**b, c, f**) or 72 h (**e**) before LC-MS/MS analysis. All data represent the mean of *n* = 6 (**b**, columns 1, 2, and 3), *n* = 4 (**b**, columns 4–8), or *n* = 3 (**c, f**) biologically independent samples (open circles in **b, c, f**) and error bars show standard deviation. Student’s two-tailed *t*-test: *P < 0.05, **P < 0.01, ***P < 0.001. Unless otherwise indicated, statistical significance is shown relative to the corresponding control (i.e., CEN.PK2). Source data of Fig. 2b, c, e and f are provided as a Source Data file

The tropine moiety of TAs is derived from arginine via putrescine, a polyamine required for ribosome biogenesis and mRNA translation^24^. Putrescine and other polyamine concentrations are regulated to remain low during normal cell growth^25–27^. We focused on engineering a putrescine-overproducing strain by overexpressing native genes involved in arginine metabolism and polyamine biosynthesis (Fig. 2a). Glutamate *N-*acetyltransferase (Arg2p) catalyzes the first step in arginine biosynthesis from glutamate. The guanidinium group of arginine is removed by an arginase (Car1p) to produce ornithine in the mitochondria, which is exported to the cytosol by an ornithine transporter (Ort1p). Cytosolic ornithine is decarboxylated to putrescine by an ornithine decarboxylase (ODC; Spe1p). Putrescine is also produced by dealkylation of spermine and spermidine by a polyamine oxidase (Fms1p).

To examine the effect of overexpression of these enzymes on putrescine production, we co-transformed wild-type yeast (CEN.PK2) with combinations of three low-copy plasmids, expressing *SPE1* (pCS4211), *ORT1* (pCS4194), *CAR1* (pCS4195), *ARG2* (pCS4196), *FMS1* (pCS4199), or blue fluorescent protein (BFP; pCS4208, 4212, 4213) as a negative control. We quantified putrescine accumulation in the medium following 48 h of growth by LC-MS/MS (Fig. 2b). *SPE1* overexpression resulted in a 13-fold increase in putrescine titer to 23 mg/L. While co-overexpression of *CAR1* or *ARG2* with *SPE1* resulted in 28 and 13% increases in putrescine titer relative to *SPE1* alone, overexpression of *ORT1* with *SPE1* caused a 35% decrease in putrescine. *ORT1* overexpression caused impairment in growth rate, which may contribute to decreased putrescine production. Overexpression of any three of *SPE1*, *CAR1*, *ARG2*, and *FMS1* increased putrescine titers to ~35 mg/L. The results suggest that a greater than 30-fold improvement in production of putrescine, a metabolite whose biosynthesis is regulated to remain at low levels (~1.5 mg/L), is achieved by overexpressing combinations of four genes: *SPE1*, *CAR1*, *ARG2*, and *FMS1*.

We leveraged the diversity of polyamine pathways from other organisms to further increase putrescine production in yeast. These alternate biosynthetic routes differ in the order in which functional groups are removed from arginine (Fig. 2a). Many bacteria and plants express an alternate route through which arginine is decarboxylated by arginine decarboxylase (ADC) to agmatine. In plants, the guanidine group of agmatine is converted to a urea by an iminohydrolase (AIH) to produce *N-*carbamoylputrescine (NCP), from which the amide group is removed by an amidase (CPA) to yield putrescine^28^. Some bacteria have an agmatine ureohydrolase (AUH) that removes the guanidine group from agmatine to directly produce putrescine^29^.

To reconstruct the heterologous putrescine pathways, we selected an ADC from *Avena sativa* (*AsADC*) with demonstrated activity in *S. cerevisiae*^29^, an AIH from *Arabidopsis thaliana* (*AtAIH*), two CPA orthologs from *Solanum lycopersicum* (*SlCPA*) and *A. thaliana* (*AtCPA*), an AUH from *E. coli* (*speB*), and an arginase from *A. thaliana* (*AtARGAH2*) with demonstrated ureohydrolase activity^28^. We reconstituted the three-step (arginine → agmatine → NCP → putrescine) or two-step (arginine → agmatine → putrescine) putrescine pathways in a stepwise fashion by co-transforming the wild-type strain with plasmids expressing *AsADC* (pCS4225), *AtAIH* (pCS4226), and either *SlCPA* (pCS4222) or *AtCPA* (pCS4221); or *AsADC* and either *speB* (pCS4223) or *AtARGAH2* (pCS4224). All transformations were performed with three low-copy plasmids, using a BFP negative control plasmid (pCS4208, 4212, 4213). We analyzed relative accumulation of agmatine, NCP, and putrescine in the medium by LC-MS/MS after 48 h (Fig. 2c, Supplementary Fig. 2). Reconstitution of the plant-specific pathway comprising AsADC, AtAIH, and AtCPA enabled putrescine titers of 23 mg/L, a 22-fold improvement relative to wild-type titers. SlCPA was poorly functional and enabled putrescine titers of 4.5 mg/L when combined with AsADC and AtAIH, similar to levels when expressing only AsADC and AtAIH. Reconstitution of the bacterial shortcut pathway via AsADC and SpeB enabled putrescine titers of 34 mg/L, 32-fold higher than wild-type. Despite reports of AtARGAH2 exhibiting activity in *S. cerevisiae*^28^, we observed virtually no activity when this enzyme was co-expressed with AsADC.

We examined overexpression of the native pathway with the reconstituted plant-bacterial shortcut pathway to increase putrescine titers. We combined the top-performing triad of overexpressed native genes (*SPE1*, *ARG2*, *CAR1*) with the top-performing heterologous putrescine pathway (*AsADC*, *speB*) by co-transforming the wild-type strain with a low-copy plasmid encoding *SPE1*, *AsADC*, and *speB* (pCS4239) and low-copy plasmids encoding *ARG2* (pCS4196) and *CAR1* (pCS4216), and measured putrescine titers in the medium by LC-MS/MS after 48 h. The resulting strain (CSY1227) produced putrescine at titers of 47 mg/L, greater than either pathway individually (CSY1225, CSY1226) but less than expected for a purely additive effect (Fig. 2f), indicating that polyamine regulatory mechanisms may be limiting putrescine production.

Yeast polyamine biosynthesis is regulated by several mechanisms (Fig. 2d). Methylthioadenosine phosphorylase (Meu1p) catalyzes the driving step in the recycling pathway for decarboxylated S-adenosylmethionine (dcSAM) from methylthioadenosine (MTA), which inhibits the activity of spermidine synthase (Spe3p)^25^. dcSAM-dependent alkylation of putrescine to spermidine and spermine is catalyzed by Spe3p and spermine synthase (Spe4p)^30^. We expected disruption of *MEU1* and *SPE4* to inhibit dcSAM-dependent alkylation of putrescine, reducing flux to other polyamines. Polyamine biosynthesis is also regulated by an antizyme-mediated negative feedback loop^31^, where the *OAZ1* gene encodes antizyme-1, a competitive inhibitor of ornithine decarboxylase (Spe1p). A polyamine-induced ribosomal frameshift enables translation of full-length antizyme at high polyamine levels, thereby imposing feedback inhibition. We expected disruption of *OAZ1* to eliminate this source of feedback inhibition limiting putrescine production. Finally, polyamine uptake is mediated by a signaling pathway involving Agp2p, a plasma membrane permease, and Sky1p, a protein kinase thought to interact with Agp2p and whose deletion reduces uptake of spermidine and spermine^27,32^. We expected disruption of *AGP2* and *SKY1* to reduce accumulation of spermidine and spermine, decreasing polyamine-induced expression of Oaz1p and alleviating Spe1p inhibition.

We examined deregulation of polyamine biosynthesis regulatory mechanisms to increase putrescine production by constructing single-gene disruptions for *MEU1*, *OAZ1*, *SPE4*, *SKY1*, and *AGP2* by inserting nonsense mutations within each open reading frame in wild-type yeast. We overexpressed ODC (*SPE1*) or co-expressed *AsADC* and *speB* from low-copy plasmids (pCS4225, 4223) in each of the single-gene disruption strains, and measured putrescine titers in the medium via LC-MS/MS after 72 h (Fig. 2e). *MEU1* and *OAZ1* disruption improved putrescine titers by 68 and 174%, respectively, when the native pathway via *SPE1* was overexpressed, but did not significantly impact titers when the heterologous *AsADC*/*speB* pathway was overexpressed, consistent with the mechanism of action for these enzymes. Disruption of *OAZ1* resulted in a 21-fold increase in putrescine titer in cells with neither the native nor heterologous pathways overexpressed, highlighting the impact of the antizyme feedback inhibition system in restricting putrescine biosynthesis. Disruption of *SKY1* and *AGP2* resulted in 29 and 14% respective increases in putrescine titer when overexpressed with *SPE1*, but decreased titers by 41% with expression of *AsADC* and *speB*. Disruption of *SPE4* did not impact putrescine production in the context of *SPE1* overexpression or *AsADC* and *speB* expression. These results indicate that substantial improvements to putrescine production via the native Spe1p-dependent pathway may be achieved by disrupting *MEU1* and *OAZ1*.

Finally, we combined the *MEU1* and *OAZ1* knockouts with overexpression of the native and heterologous putrescine biosynthetic genes. We integrated additional copies of the genes *ARG2*, *CAR1*, and *FMS1* into the genome of a *meu1*/*oaz1* double-disruption strain (CSY1234), and transformed this strain with a low-copy plasmid expressing *SPE1*, *AsADC*, and *speB* (pCS4239), resulting in strain CSY1235. LC-MS/MS analysis of the medium of CSY1235 indicated that putrescine titers reached 86 mg/L after 48 h (Fig. 2f), representing a 71-fold improvement relative to wild-type yeast.

### Optimization of de novo N-methylpyrrolinium production

We proceeded with reconstitution of the subsequent biosynthetic steps towards tropine. The first two enzymes in the tropine pathway branch between putrescine and NMPy are common to nicotine biosynthesis^33^. Putrescine is converted to *N-*methylputrescine (NMP) by a SAM-dependent *N-*methyltransferase (PMT), which is oxidized to 4-methylaminobutanal (4MAB) by a copper-dependent diamine oxidase (MPO). 4MAB is unstable in aqueous solution and spontaneously cyclizes via intramolecular nucleophilic attack to form NMPy (Fig. 1).

**Fig. 3:**
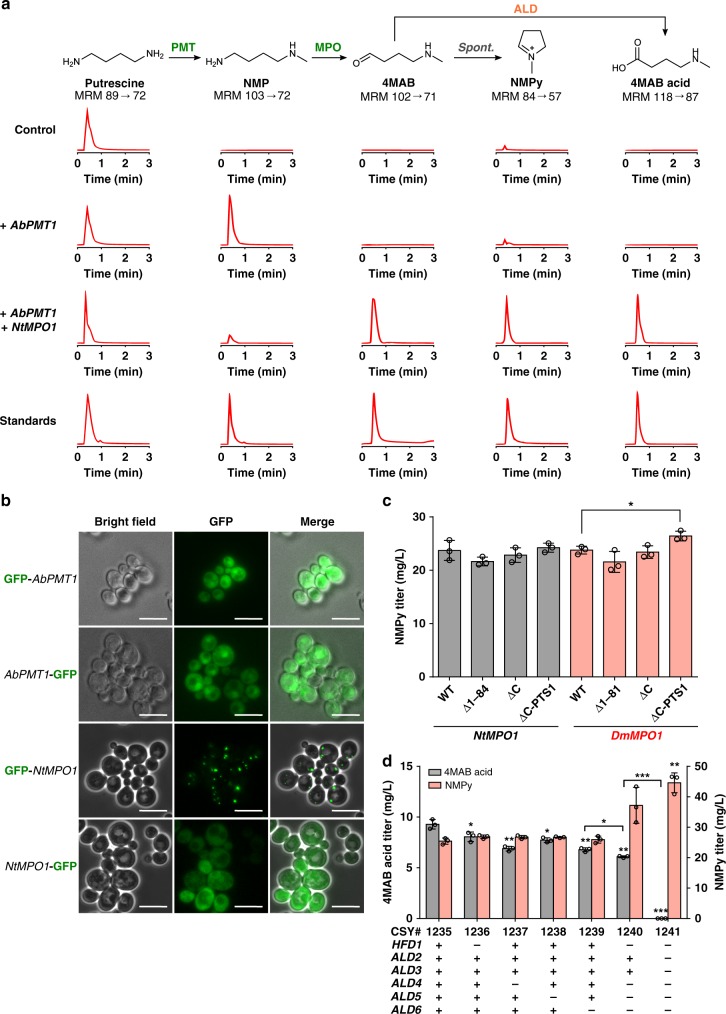
Optimization of *N-*methylpyrrolinium production in yeast. **a** Engineered biosynthetic pathway for production of *N-*methylpyrrolinium (NMPy) from putrescine and stepwise substrate and product tracking. The proposed mechanism for formation of the 4MAB acid side product via activity of an endogenous yeast enzyme (ALD) is shown. Extracted ion chromatogram MRM traces are shown for each metabolite along the pathway and for authentic standards using the highest precursor ion/product ion transition for each metabolite (Supplementary Table 3). Control represents strain CSY1235 expressing *SPE1*, *AsADC*, and *speB* on a low-copy plasmid (pCS4239). Chromatogram traces are representative of three biological replicates. Enzyme symbols: PMT, putrescine *N-*methyltransferase; MPO, *N-*methylputrescine oxidase; ALD, aldehyde dehydrogenase. **b** Characterization of the subcellular localization of putrescine *N-*methyltransferase and *N-*methylputrescine oxidase by epifluorescence microscopy. Microscopy was performed on wild-type yeast expressing *N-* or *C-*terminal GFP-tagged *AbPMT1* or *NtMPO1* from low-copy plasmids. Scale bar, 10 μm. **c** Effect of *N-* and *C-*terminal truncations to methylputrescine oxidase on NMPy production in engineered yeast. Wild-type (WT) enzymes and indicated truncations were expressed from low-copy plasmids (pCS4233-4238) in CSY1235 together with *SPE1*, *AsADC*, and *speB* (pCS4239) and *AbPMT1* (pCS4193). **d** Effect of aldehyde dehydrogenase gene disruptions on production of the 4MAB acid side product and NMPy in engineered yeast. Plus and minus symbols indicate presence or absence of functional enzyme, respectively. For **a**, **c**, **d**, strains were cultured in selective (YNB-DO) media with 2% dextrose at 30 °C for 48 h before LC-MS/MS analysis. All data represent the mean of *n* = 3 biologically independent samples (open circles in **c,** **d**) and error bars show standard deviation. Student’s two-tailed *t*-test: *P < 0.05, **P < 0.01, ***P < 0.001. Unless otherwise indicated, statistical significance is shown relative to the corresponding control (CSY1235). Source data of Fig. 3c and d are provided as a source data file

We focused on optimizing NMPy production in our putrescine-overproducing strain. We co-transformed strain CSY1235, which harbors a low-copy plasmid expressing *SPE1*, *AsADC*, and *speB* (pCS4239), with low-copy plasmids expressing a PMT from *A. belladonna* (*AbPMT1*; pCS4193) and a MPO from *Nicotiana tabacum* (*NtMPO1*; pCS4218). All transformations were performed with a three-plasmid system as described above. We compared intermediate accumulation in the medium from cells expressing each successive enzyme between putrescine and NMPy by LC-MS/MS after 48 h. Although the product of NtMPO1 (4MAB) and its spontaneous cyclization product (NMPy) were detectable with expression of AbPMT1 and NtMPO1 (Fig. 3a), levels were far lower than their precursors, NMP and putrescine (Supplementary Fig. 3). We verified that *MEU1* disruption and its impact on SAM recycling did not inhibit putrescine *N-*methylation by AbPMT1 (Supplementary Fig. 4).

We initially suspected that the low 4MAB/NMPy levels might be due to poor expression and/or activity of NtMPO1, possibly due to an unfavorable chemical environment resulting from incorrect subcellular localization. We explored improvements in MPO activity through a combination of subcellular localization studies, identification of MPO orthologs, and enzyme truncations (Supplementary Note 1). Although localization of MPO activity to the cytosol from the peroxisome (Supplementary Fig. 5b, Fig. 3b) did not yield improvement in NMPy production (Supplementary Fig. 5c), a C-terminally truncated MPO ortholog from *Datura metel* (*DmMPO1*^*ΔC-PTS1*^) resulted in 55 and 11% respective increases in 4MAB and NMPy titers relative to NtMPO1 (Supplementary Figure 6d, Fig. 3c). All further optimization was performed using *DmMPO1*^*ΔC-PTS1*^. 4MAB and NMPy levels remained lower than expected given the majority of NMP was consumed upon MPO expression (Fig. 3a, Supplementary Fig. 3), suggesting side reaction(s) that consume the product(s) of MPO, drawing flux away from TA biosynthesis. We observed accumulation of 4MAB acid by LC-MS/MS after 48 h when *AbPMT1* and *DmMPO1*^*ΔC-PTS1*^ were co-expressed from low-copy plasmids (pCS4193, 4238) in the putrescine-overproducing strain (CSY1235), but no accumulation in the absence of MPO (Fig. 3a), indicating that aldehyde dehydrogenases may be oxidizing 4MAB.

We examined the role of six gene(s) (*ALD2*-*ALD6*, *HFD1*) encoding enzymes with aldehyde dehydrogenase activity^34,35^ in 4MAB acid production. *ALD2* and *ALD3* encode nearly identical cytosolic dehydrogenases, which catalyze the oxidation of 3-aminopropanal to β-alanine^36^. *ALD4*, *ALD5*, and *ALD6* encode mitochondrial and cytosolic acetaldehyde dehydrogenases, which oxidize acetaldehyde to acetate during fermentative growth^37^ and an array of aliphatic and aromatic aldehydes to carboxylic acids^34^. Based on the annotated dehydrogenase activity of *HFD1* and *ALD4-6* on 4-aminobutanal in the KEGG database, we constructed individual knockouts strains for these targets (CSY1236-1239) by inserting a series of nonsense mutations within their open reading frames in the putrescine-overproducing strain (CSY1235). We co-expressed *AbPMT1* and *DmMPO1*^*ΔC-PTS1*^ from low-copy plasmids (pCS4193, 4238) in each single disruption strain and measured 4MAB acid accumulation in the media by LC-MS/MS after 48 h. The *HFD1* and *ALD4-6* disruptions resulted in 13–27% decreases in 4MAB acid titer and marginal (<6%) increases in 4MAB or NMPy titer (Supplementary Fig. 7, Fig. 3d).

Although *ALD4*-*6* are considered essential genes for their role in acetate and acetyl-CoA production^37^, the genes are partially redundant and the lethal phenotype of double and triple knockouts can be rescued by supplementing media with acetate^37,38^. We constructed a quadruple knockout strain (CSY1240) with disruptions to *HFD1* and *ALD4-6*, and which expressed *AbPMT1* and *DmMPO1*^*ΔC-PTS1*^ from low-copy plasmids (pCS4193, 4238). This strain showed a 34% reduction in 4MAB acid and 33 and 45% respective increases in 4MAB and NMPy titers compared to the strain with no disruptions (CSY1235) (Supplementary Fig. 7, Fig. 3d), indicating at least one other aldehyde dehydrogenase remained active on 4MAB. Although Ald2p and Ald3p do not oxidize the same diversity of substrates as Ald4p-Ald6p, the similarity of 4MAB to the Ald2p/Ald3p native substrate, 3-aminopropanal, may enable them to oxidize 4MAB. We constructed an *ALD*-null strain (CSY1241) by deleting the *ALD2-ALD3* genes from the quadruple knockout strain (CSY1240) and co-expressing *AbPMT1* and *DmMPO1*^*ΔC-PTS1*^ from low-copy plasmids (pCS4193, 4238). The presence of pantothenate in the media enabled the *ALD*-null strain to grow with no additional nutrient supplementation. Following 48 h of growth, LC-MS/MS analysis revealed that disruption of all *ALD* genes eliminated 4MAB acid and increased 4MAB and NMPy titers by 59% and 76%, respectively, compared to the strain with all genes intact (Supplementary Fig. 7, Fig. 3d). We constructed an optimized NMPy-producing strain by integrating a putrescine-overproduction gene cassette (pCS4239; *SPE1*, *AsADC*, *speB*) into the genome of the *ALD*-null strain (CSY1241) to create CSY1242, and integrated *AbPMT1* and *DmMPO1*^*ΔC-PTS1*^ to create CSY1243. We monitored production of putrescine, NMP, and NMPy by CSY1243 in shake-flask cultures over 96 h (Supplementary Fig. 8). LC-MS/MS analysis indicated that accumulation of putrescine and NMP was minimal (5.1 mg/L and 0.033 mg/L, respectively) and NMPy titers reached 40 mg/L.

### Optimization of de novo tropine biosynthesis in yeast

**Fig. 4:**
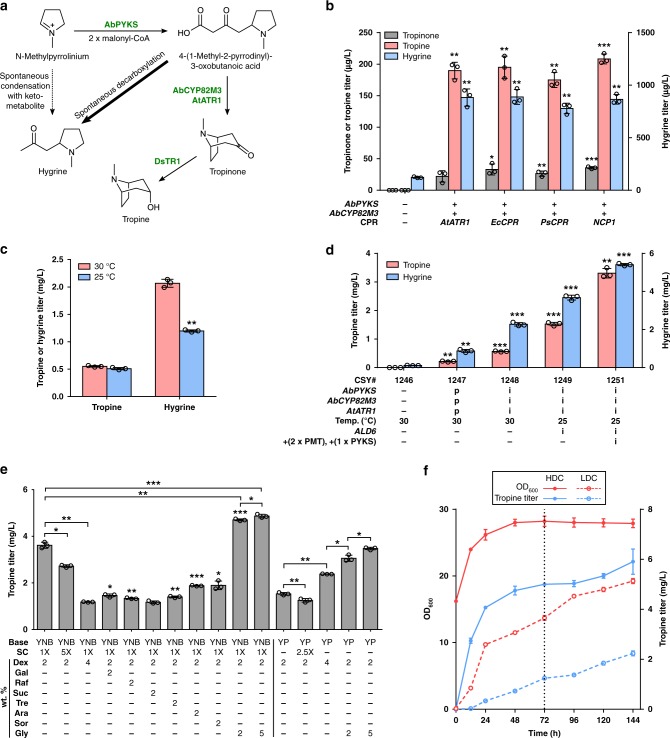
Optimization of de novo tropine biosynthesis in yeast. **a** Engineered pathway for production of tropine from *N-*methylpyrrolinium and proposed spontaneous side reactions producing hygrine. Putative major and minor side reactions in yeast are indicated by bold and dotted arrows, respectively. **b** Production of tropine and related intermediates with expression of *AbPYKS*, *AbCYP82M3*, and *DsTR1* in engineered yeast. Indicated genes were expressed from low-copy plasmids in CSY1246; plus and minus symbols indicate presence or absence of enzyme. **c** Effect of growth temperature on spontaneous hygrine production in CSY1248. **d** Summary of genetic optimization to increase tropine production in engineered yeast. Minus symbol indicates absence of gene; ‘p’ and ‘i’ indicate gene expression from low-copy plasmid or genomic integration, respectively. Strains were cultured in selective (YNB-DO) media (**b, d**: CSY1247) or non-selective (YNB-SC) media (**c, d**: CSY1246, 1248, 1249, 1251) with 2% dextrose at 30 °C or 25 °C for 48 h before LC-MS/MS analysis. **e** Optimization of media conditions to improve tropine production in CSY1251. Minus symbols indicate absence of media component. Strains were cultured for 72 h prior to LC-MS/MS analysis. YNB yeast nitrogen base, YP yeast extract plus peptone, SC synthetic complete amino acid mixture, Dex dextrose, Gal galactose, Raf raffinose, Suc sucrose, Tre trehalose, Ara arabinose, Sor sorbitol, Gly glycerol. For **b**–**e**, data represent the mean of *n* = 3 biologically independent samples (open circles) and error bars show standard deviation. Student’s two-tailed *t*-test: *P < 0.05, **P < 0.01, ***P < 0.001. **f** Cell growth and tropine production in shake-flask cultures of CSY1251 over 6 days. Low-density (LDC) and high-density (HDC) cultures were grown in non-selective media (YNB-G; YNB + 1 × amino acids + 2% dextrose + 5% glycerol) at 25 °C and 300 rpm. At *t* = 72 h (dotted vertical line), cultures were supplemented with additional amino acids, dextrose, and glycerol to final concentrations of 1×, 2%, and 2%, respectively. Metabolite titers were quantified by LC-MS/MS. Data indicate the mean of *n* = 3 biologically independent samples and error bars show standard deviation. Source data of Fig. 4b–f are provided as a source data file

The precise mechanism and associated enzymes for the biosynthesis of tropinone from NMPy remained unknown until recently, when a root-expressed PKS and cytochrome P450 were described in *A. belladonna*^19^. The identified PKS, pyrrolidine ketide synthase (PYKS), uses the activated pyrrolinium moiety to initiate condensation with two malonyl-CoA units to form MPOB. The P450 enzyme, tropinone synthase (CYP82M3), catalyzes the second ring closure to tropinone by reforming the unsaturated pyrrolinium group via hydroxylation and subsequent dehydration^19^. Tropinone is reduced by a stereospecific reductase, tropinone reductase 1 (TR1), to produce tropine^18^ (Fig. 4a).

We obtained yeast codon-optimized DNA sequences encoding *A. belladonna* pyrrolidine ketide synthase (*AbPYKS*), tropinone synthase (*AbCYP82M3*), *Datura stramonium* tropinone reductase I (*DsTR1*), and four different CPRs, including plant CPRs from *A. thaliana*, *Eschscholzia californica*, and *Papaver somniferum* and the yeast CPR (*NCP1*). We constructed strain CSY1246 by integrating *DsTR1* into the genome of the NMPy-producing strain (CSY1243), and expressing *AbPYKS* (pCS4246), *AbCYP82M3* (pCS4247), and each CPR (pCS4200-4203) from low-copy plasmids. We monitored accumulation of NMPy, MPOB, tropinone, and tropine by LC-MS/MS in the media after 48 h (Fig. 4b, Supplementary Fig. 9b). Tropine production levels were comparable (175–210 μg/L) with all CPR partners. Although accumulation of tropinone, the product of AbCYP82M3, was minimal, a substantial portion of MPOB remained unconsumed by AbCYP82M3 (Supplementary Fig. 9b), implicating the P450 enzyme as a primary bottleneck (Supplementary Discussion). In addition, hygrine, a derivative of NMPy, accumulated to titers almost four-fold greater than tropine (775–900 μg/L). Hygrine accumulation had previously been observed during in vitro characterization of AbPYKS and AbCYP82M3 via spontaneous decarboxylation of MPOB^19^ (Fig. 4a). We observed reduced hygrine accumulation in a control strain lacking AbPYKS and AbCYP82M3, suggesting a second mechanism for hygrine production in the yeast environment. We hypothesized that a metabolite present in the media and/or produced by yeast might undergo spontaneous decarboxylative condensation with NMPy to form hygrine (Fig. 4a). We performed co-substrate feeding experiments with NMPy-producing and –non-producing strains, which suggested that acetate supplementation may increase levels of an endogenous keto-metabolite, such as acetoacetate or acetoacetyl-CoA, which condenses with NMPy to form hygrine (Supplementary Note 2).

Plasmid-based overexpression of eukaryotic P450s can impose a significant burden on yeast cells due to overproliferation of ER membranes^39^. We examined integration of the tropine biosynthesis genes into the yeast genome to enable more stable *AbCYP82M3* expression and improve tropine production. Given the ability of the *A. thaliana* CPR (AtATR1) to pair with various plant P450 enzymes^7^, we constructed a tropine-producing platform strain (CSY1248) by integrating *AtATR1, AbPYKS*, and *AbCYP82M3* into the genome of CSY1246. We compared tropine and hygrine accumulation for CSY1248 to plasmid-based expression (pCS4246, 4247, 4200) in CSY1246 (denoted CSY1247) via LC-MS/MS analysis after 48 h (Fig. 4d). Genomic expression of *AbPYKS*, *AbCYP82M3*, and *AtATR1* increased tropine titers by nearly three-fold (565 μg/L) relative to plasmid-based expression (214 μg/L), likely due to more consistent P450 expression and decreased auxotrophic burden^39^. Hygrine accumulation also increased by 2.6-fold, suggesting that genomic integration may improve *AbPYKS* expression and result in increased MPOB production and decarboxylation to hygrine.

We examined the impact of temperature on spontaneous hygrine production. As *A. belladonna* and other TA-producing *Solanaceae* are adapted to cooler climates, we suspected that growth of strains expressing *Solanaceae* genes at 25 °C may improve enzyme folding and/or activity, enabling comparable production of tropine to that at 30 °C while reducing the rate of spontaneous hygrine production. We grew cultures of the tropine-producing strain (CSY1248) at 30 °C and 25 °C and compared the accumulation of tropine and hygrine via LC-MS/MS analysis of the medium after 48 h. While tropine titers were minimally impacted by the decrease in temperature, hygrine accumulation was decreased by 42% at 25 °C compared to at 30 °C, resulting in a 60% increase in the ratio of tropine to hygrine produced (Fig. 4c). All further experiments for tropine production were performed at 25 °C. We attempted to reduce hygrine production from spontaneous condensation with an endogenous acetate-derived metabolite by eliminating acetate auxotrophy. We observed that reconstitution of *ALD6* in CSY1249 resulted in a 2.7-fold increase in tropine titers (1.5 mg/L) relative to CSY1248 (565 μg/L) despite a 1.6-fold increase in hygrine accumulation (Fig. 4d), and that elimination of acetate auxotrophy may improve metabolite flux through the entire pathway (Supplementary Note 3).

### Optimization of flux bottlenecks and media conditions

To identify pathway bottlenecks between putrescine and tropine, we expressed an additional copy of *AbPMT1* (pCS4193), *DmMPO1*^*ΔC-PTS1*^ (pCS4238), *AbPYKS* (pCS4246), *AbCYP82M3* (pCS4247), or *DsTR1* (pCS4310) from low-copy plasmids in CSY1249 and compared intermediate production to that of CSY1249 expressing BFP (pCS4208, pCS4212, or pCS4213) by LC-MS/MS after 48 h. Additional copies of *AbCYP82M3, DmMPO1*^*ΔC-PTS1*^, or *DsTR1* resulted in no significant changes to intermediate levels (Supplementary Fig. 12b, d, e). An additional copy of *AbPMT1* resulted in increases in titers of intermediates downstream of putrescine, including an over two-fold increase in tropine titers (Supplementary Fig. 12a), suggesting that PMT was limiting pathway flux. A 26% increase in tropine titer was observed with an additional copy of *AbPYKS* (Supplementary Fig. 12c). We constructed an optimized tropine-producing strain by integrating two additional copies of PMT from *A. belladonna* (*AbPMT1*) and *D. stramonium* (*DsPMT1*) and an additional copy of *AbPYKS* in CSY1249. The resulting strain (CSY1251) showed substantial (1.4 to 5.3-fold) increases in the production of intermediates downstream of putrescine and produced tropine at titers of 3.3 mg/L after 48 h, over double that of CSY1249 (Supplementary Fig. 13, Fig. 4d).

We examined the impact of media composition on tropine production in CSY1251 and observed that under the best media condition (yeast nitrogen base + 1X amino acids + 2% dextrose + 5% glycerol; denoted YNB-G), tropine titers reached 4.9 mg/L after 72 h, 35% greater than with 2% dextrose alone (Fig. 4e; Supplementary Note 4). To model enhanced oxygen transfer in a scaled-up culture and identify remaining pathway bottlenecks, we performed time course experiments by culturing CSY1251 in YNB-G in 50-mL shake flasks and monitored metabolite titers by LC-MS/MS for 6 days. We examined low-density cultures (LDCs), wherein initial cell density is low (OD ~ 0.1–0.5), and high-density cultures (HDCs) in which cells are grown to a moderate initial density (OD ~ 15) and transferred to fresh media. We approximated a fed-batch culture by supplementing media with dextrose, glycerol, and amino acids after three days. Tropine titers reached 5.9 mg/L in HDC (OD_final_ ~ 28) and 2.2 mg/L in LDC (OD_final_ ~ 19), highlighting the importance of starting culture density (Fig. 4f). Although NMP and tropinone did not accumulate appreciably, titers of hygrine, putrescine, and NMPy reached 10, 30, and 30 mg/L, respectively, in HDC (Supplementary Fig. 14). Overall, our strategies for optimizing tropine production resulted in a 28-fold improvement in titer to nearly 6 mg/L.

### De novo production of the non-canonical TA cinnamoyltropine

Medicinally relevant TAs such as hyoscyamine and scopolamine are derived from littorine, the product of tropine condensation with phenyllactate. Although the enzymes catalyzing this reaction have not yet been identified in *Solanaceae*, analogous TAs and associated acyltransferases have been identified in *Erythroxylaceae*, an evolutionarily distant lineage of plants which are thought to have evolved the ability to produce TAs independently^40^. An acyltransferase denoted cocaine synthase from *Erythroxylum coca* (EcCS) catalyzes condensation of methylecgonine (2-carbomethyoxy-3β-tropine) with benzoic acid in the final step of the cocaine biosynthetic pathway^41^. EcCS has been demonstrated to condense various fed acyl acceptors and donors when expressed in yeast, including metabolites from plant phenylpropanoid pathways^42^. We used our engineered yeast platform to combine TA biosynthetic genes from *Solanaceae* and *Erythroxylaceae* with an acyl donor biosynthetic module to enable de novo production of TAs.

We selected cinnamic acid, a phenylpropanoid intermediate^10^ whose biosynthesis in yeast has been demonstrated^43^, as the acyl donor for condensation with tropine by EcCS (Fig. 1). Cinnamate can be produced from phenylalanine via a phenylalanine ammonia-lyase from *A. thaliana* (AtPAL1). Since EcCS requires a coenzyme A (CoA)-activated acyl donor, we used a 4-coumarate-CoA ligase from *A. thaliana* (At4CL5) with activity on cinnamate^44^ to enable cinnamoyl-CoA biosynthesis. We obtained yeast codon-optimized DNA sequences for these genes and assembled them into a two-plasmid expression system. *AtPAL1* was expressed from a low-copy plasmid (pCS4252), whereas *At4CL5* and *EcCS* were expressed from a high-copy plasmid (pCS4207) to compensate for the low activity of EcCS on non-native substrates. We expressed *AtPAL1*, *At4CL5*, and *EcCS* in our tropine-producing strain (CSY1251), denoted CSY1282, and measured accumulation of the product, cinnamoyl-3α-tropine (referred to as cinnamoyltropine), in the medium via LC-MS/MS after 72 h.

**Fig. 5:**
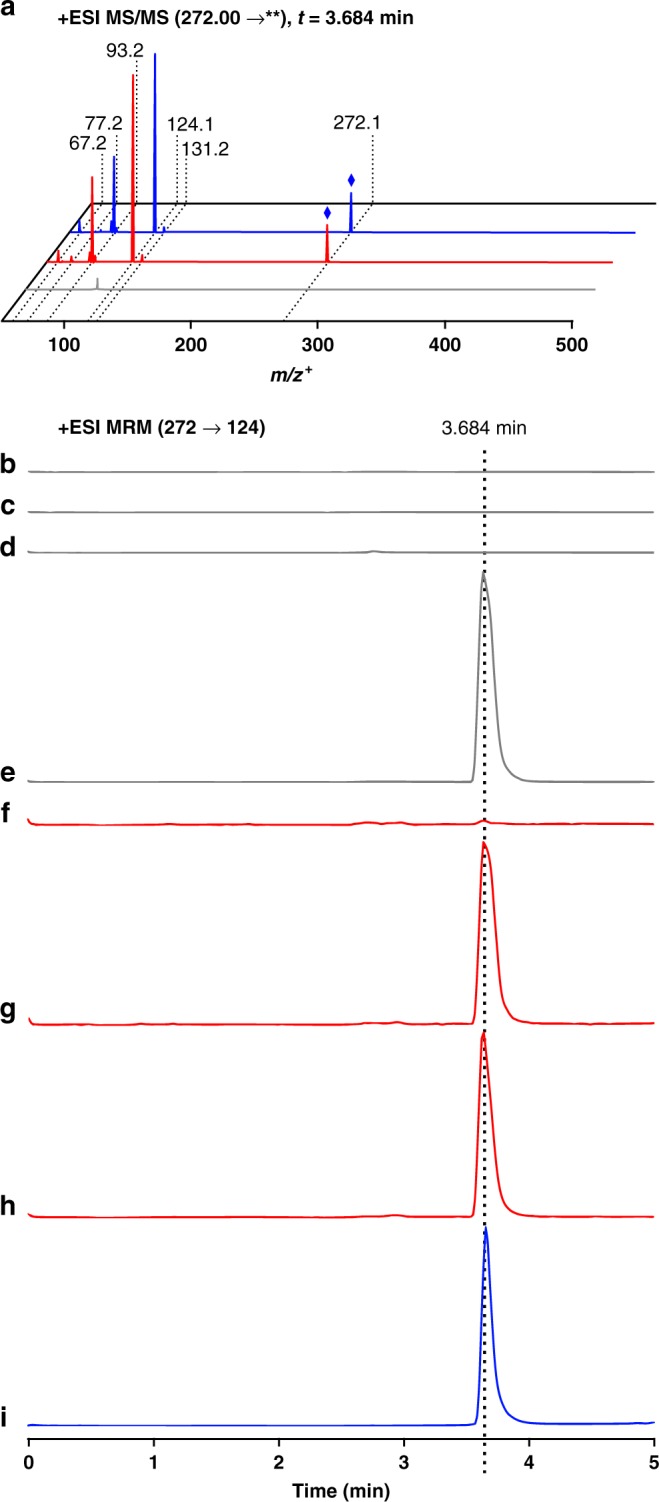
De novo production of the non-canonical tropane alkaloid cinnamoyltropine in yeast. **a** Tandem MS/MS spectra of extracellular medium of CSY1251 (gray), CSY1282 (red), or a genuine cinnamoyltropine standard (blue) for a parent mass of *m/z*^+^ = 272. Blue diamond indicates parent compound peak. **b**–**i** Validation of EcCS acyltransferase activity on cinnamic acid and α-tropine via substrate feeding. Strains were transformed with combinations of plasmids expressing *AtPAL1* (low-copy plasmid pCS4252) and/or *At4CL5* and *EcCS* (high-copy plasmid pCS4207), and then cultured in media with different supplemented substrates, as follows: **b** CEN.PK2 + At4CL5 + EcCS + 0.1 mM *trans*-cinnamic acid; **c** CEN.PK2 + At4CL5 + EcCS + 0.5 mM α-tropine; **d** CEN.PK2 + AtPAL1 + At4CL5 + EcCS; **e** CEN.PK2 + AtPAL1 + At4CL5 + EcCS + 0.5 mM α-tropine; **f** CSY1251 + At4CL5 + EcCS; **g** CSY1251 + At4CL5 + EcCS + 0.2 mM *trans*-cinnamic acid; **h** CSY1251 + AtPAL1 + At4CL5 + EcCS; **i** 25 nM cinnamoyltropine standard. For **a**–**h**, yeast strains were cultured in selective media (YNB-DO + 2% dextrose + 5% glycerol) at 25 °C for 72 h prior to LC-MS/MS analysis. All traces are representative of *n* = 3 biologically independent samples

We used tandem MS/MS and fragmentation analysis to verify the identity of this product. Comparison of MS/MS spectra corresponding to the parent mass of cinnamoyltropine (*m/z*^+^ = 272) revealed a peak at a retention time of 3.684 min for CSY1282 but not for CSY1251, which produced fragments whose retention time and mass transitions were identical to those generated by a cinnamoyl-3α-tropine standard (Fig. 5a). The primary *m/z*^+^ 272 → 124 transition, which is consistent with the *m/z*^+^ = 124 tropine fragment produced during fragmentation of hyoscyamine^45^, was used to develop a multiple reaction monitoring (MRM) LC-MS/MS method and standard curve for quantification of cinnamoyltropine. The titer of cinnamoyltropine produced by CSY1282 after 72 h was 6.0 μg/L. We performed substrate feeding experiments in tropine-producing and –non-producing strains to elucidate the stereochemistry of the EcCS-catalyzed reaction (Supplementary Note 5, Fig. 5b–i).

## Discussion

Although several groups have engineered prokaryotes for polyamine overproduction^46,47^, few such efforts have been made in eukaryotic hosts. An earlier study engineered *S. cerevisiae* to overproduce spermidine based on disruption of *OAZ1* and *TPO1*, resulting in a 2.7-fold increase in spermidine titers^48^. The 71-fold increase in putrescine titers observed here highlights the importance of our multi-target strategy for increasing polyamine accumulation. Although subsequent experiments (Supplementary Figs. 8, 12, 14) indicated that putrescine production was not limiting flux, future efforts for improving titers towards industrial TA production may incorporate more comprehensive alterations to nitrogen metabolism, such as partial bypass of the urea cycle or upregulation of arginine and ornithine biosynthesis via pathway rewiring and flux balancing^49^.

Ping *et al*. recently reported production of NMPy in *E. coli* and *S. cerevisiae* strains^50^. Expression of three heterologous genes-an ODC ortholog from *E. coca* and PMT and MPO variants from *Anisodus* species-enabled NMPy production at nearly 20 mg/L in yeast. Putrescine accumulation due to the heterologous ODC may have inhibited the native ODC through the antizyme-mediated feedback regulation, resulting in reduced putrescine titers (15 mg/L) relative to that achieved in our platform (86 mg/L). Consistent with our observations of 4MAB oxidation by aldehyde dehydrogenases, the prior study noted Ald4p, Ald5p, and Hfd1p convert 4MAB to 4MAB acid in vitro and deleted these genes to improve NMPy accumulation. Our work shows that disruption of Hfd1p and Ald4p-Ald6p activity eliminates less than half of the 4MAB acid, and additional disruption of Ald2p and Ald3p activity eliminates this side product. Although the possibility that Hfd1p and Ald2p-Ald6p function synergistically to oxidize 4MAB prevents drawing quantitative conclusions regarding individual enzyme contributions to this side product, our work indicates that disruption of *HFD1* and *ALD2-6* is essential for achieving high NMPy production.

Although our optimization of putrescine production enabled NMPy titers (40 mg/L) roughly two-fold greater than previously reported^50^, the conversion of putrescine to NMPy was less efficient. Data for the NMPy-producing strain (CSY1243) indicated that despite near complete consumption of putrescine and NMP, NMPy accumulated to less than half the titer observed for putrescine in CSY1235 (>80 mg/L), suggesting a leak in metabolite flux between these TA precursors. We hypothesize that some putrescine may be converted to other polyamines or amino acids due to inefficient *N-*methylation by AbPMT1 and/or oxidation by DmMPO1^ΔC-PTS1^. TA biosynthesis occurs in roots, whereas the *DmMPO1* variant was identified from a leaf transcriptome library and thus may not efficiently utilize NMP. Replacement of *DmMPO1* with a root-expressed variant with higher catalytic efficiency and improving SAM availability for the methyltransferase step may increase flux from putrescine to NMPy.

Hygrine constitutes a major side product in the biosynthesis of tropinone in yeast, corroborating a prior study that observed substantial hygrine accumulation during *in planta* and in vitro characterization of *AbPYKS* and *AbCYP82M3*^19^. While the study noted accumulation of cuscohygrine *in planta* and in vitro due to decarboxylative condensation of an additional NMPy unit with MPOB, we were unable to observe this side product in yeast, highlighting the utility of microbial hosts for the production of plant natural products with improved selectivity. Our work suggests that hygrine production in yeast likely occurs via two mechanisms: (i) spontaneous decarboxylation of MPOB, and (ii) spontaneous condensation of NMPy with an acetate-derived keto-metabolite such as acetoacetate. Although we reduced the contribution of the second mechanism by reconstituting *ALD6* to abrogate acetate auxotrophy and the contributions of both mechanisms by lowering temperature, the decrease in hygrine production was overshadowed by the positive effect of *ALD6* on pathway flux. The generation of NADPH through dehydrogenase activity of *ALD6* likely provided a more kinetically favorable NADPH:NADP^+^ ratio for activity of the cytochrome P450/CPR pairing and NADPH-dependent TRI^51^, similar to the effect of glycerol supplementation^14^ (Supplementary Note 4). Future strategies for improving flux may reduce hygrine production from spontaneous MPOB decarboxylation. For example, colocalizing *AbPYKS* and *AbCYP82M3*^52^ may reduce latency between MPOB production and oxidation. A similar colocalization strategy may be applied to enzymes involved in NMPy biosynthesis to decrease condensation of NMPy with keto-metabolites.

Although our bottleneck analysis revealed that the PMT and PYKS steps were limiting tropine flux, time course data for the final tropine-producing strain (CSY1251) revealed that key inefficiencies remain. Approximately 30 mg/L of putrescine and NMPy accumulated (Supplementary Fig. 14), indicating that low flux of putrescine into NMPy and NMPy into tropinone remain major bottlenecks. We inferred that some aspect of the PKS enzyme other than expression might be limiting, such as malonyl-CoA availability or poor inherent activity in yeast. Incorporation of strategies to increase malonyl-CoA^53–56^ and to engineer PYKS for improved catalytic efficiency^57^ may increase tropine production.

While this manuscript was under final review, Ping et al. reported the biosynthesis of tropine in yeast^23^ by expressing *A. acutangulus* PYKS, CYP82M3, and TR1 orthologs in a NMPy-producing strain^50^. Little optimization of pathway flux from NMPy to tropine was performed beyond expression of an additional copy of native *ACC1* to improve malonyl-CoA availability. Through extensive optimization of precursor production, enzyme expression, pathway bottlenecks, side reactions, and growth conditions, we achieved tropine titers (5.9 mg/L) nearly 50-fold greater than those reported in that study (0.13 mg/L), providing a robust starting point for downstream TA biosynthesis. Although many of the enzymes required for biosynthesis of medicinally important TAs, such as hyoscyamine and scopolamine, are now identified, key gaps still remain. Our tropine-producing strain offers a platform for rapidly screening and testing gene candidates for missing enzyme activities, enabling more accessible and cost-effective production of these important medicines. Tropinone and tropine are precursors for a variety of alkaloids, including calystegines and derivatives of cocaine^18^. Extension of the engineered pathway to these compounds will facilitate characterization of their associated enzymes and study of their biological applications. Finally, as demonstrated with biosynthesis of cinnamoyltropine, coupling biosynthetic modules from diverse plant lineages to produce different acyl donors and acceptors within a heterologous host enables generation of new non-canonical or non-natural TA derivatives, thereby accelerating the drug discovery pipeline.

## Methods

### Chemical compounds and standards

Putrescine dihydrochloride, *N-*methylputrescine, hygrine, tropinone, and tropine were purchased from Santa Cruz Biotechnology (Dallas, TX). 4-(Methylamino)butyric acid hydrochloride, lithium acetoacetate, and cinnamic acid were purchased from Sigma (St. Louis, MO). Pseudotropine was purchased from Chem-Impex International (Wood Dale, IL). γ-Methylaminobutyraldehyde (4MAB) diethyl acetal was purchased from Toronto Research Chemicals (Toronto, ON). 4MAB was prepared from 4MAB diethyl acetal by treatment with an equal volume of 2 M HCl for 30 min at 60 °C^58^. NMPy was prepared from 4MAB diethyl acetal by treatment with five volumes of 2 M HCl for 30 min at 60 °C, overnight incubation at room temperature, washing with three volumes of ether to remove trace organic impurities, and evaporation of residual water under vacuum^19^. Cinnamoyl-3α-tropine (catalogue number K23.208.020) was synthesized by Aurora Fine Chemicals LLC (San Diego, CA).

### NMR verification of cinnamoyl-3α-tropine standard

To verify the identity and stereochemistry of the synthesized cinnamoyltropine as the ester of *trans*-cinnamic acid and α-tropine, ^1^H-NMR was performed on the standard as well as on purchased standards of *trans*-cinnamic acid, tropine, and pseudotropine using CD_3_OD as the solvent on a Varian Inova 600 MHz spectrometer at the Stanford University NMR Facility (Stanford, CA). Spectrum analysis and processing was performed using the Mnova software package (Mestrelab Research, v12.0.4). ^1^H-NMR spectra are provided in Supplementary Figs. 15–18.

NMR data of cinnamoyltropine were as follows: ^1^H NMR (600 MHz, CD_3_OD) δ 7.72 (d, J = 16.0 Hz, 1 H), 7.64 (m, 2 H), 7.43 (m, 3 H), 6.58 (dd, J = 16.1, 1.4 Hz, 1 H), 5.16 (t, J = 5.2 Hz, 1 H), 3.87 (d, J = 5.3 Hz, 2 H), 2.81 (s, 3 H), 2.45 (m, 4 H), 2.35 (m, 2 H), 2.19 (d, J = 16.2 Hz, 2 H).

NMR data of *trans*-cinnamic acid were as follows: ^1^H NMR (600 MHz, CD_3_OD) δ 7.66 (d, J = 16.0 Hz, 1 H), 7.59 (dd, J = 6.3, 2.5 Hz, 2 H), 7.40 (m, 3 H), 6.48 (dd, J = 15.9, 1.1 Hz, 1 H).

NMR data of tropine were as follows: ^1^H NMR (600 MHz, CD_3_OD) δ 3.93 (t, J = 5.1 Hz, 1 H), 3.09 (p, J = 2.9 Hz, 2 H), 2.26 (s, 3 H), 2.13 (td, J = 6.8, 6.2, 1.1 Hz, 2 H), 2.02 (m, 4 H), 1.71 (d, J = 14.5 Hz, 2 H).

NMR data of pseudotropine were as follows: ^1^H NMR (600 MHz, CD_3_OD) δ 3.84 (tt, J = 11.2, 6.2 Hz, 1 H), 3.15 (p, J = 2.9 Hz, 2 H), 2.26 (s, 3 H), 2.01 (m, 2 H), 1.77 (ddd, J = 12.9, 5.7, 2.8 Hz, 2 H), 1.59 (m, 4 H).

### Plasmid construction

Oligonucleotides used in this work were synthesized by the Stanford Protein and Nucleic Acid Facility (Stanford, CA) and are listed in Supplementary Data 1. Biosynthetic genes used in this study are listed by source and accession number in Supplementary Table 1. Native yeast genes were amplified from *S. cerevisiae* genomic DNA via colony PCR^59^. Gene sequences for heterologous enzymes were codon-optimized to improve expression in *S. cerevisiae* using GeneArt GeneOptimizer software (Thermo Fisher Scientific) and synthesized as either gBlock DNA fragments (Integrated DNA Technologies, IDT) or gene fragments (Twist Bioscience). Plasmids used in this study are listed in Supplementary Data 2. Two types of plasmids were used for gene expression in yeast: direct expression (DE) plasmids for testing biosynthetic genes of interest and yeast integration (YI) holding plasmids to provide a template for genomic integration of selected promoter-gene-terminator cassettes.

DE plasmids comprised a gene of interest flanked by a constitutive promoter and terminator, a low-copy CEN6/ARS4 yeast origin of replication, and an auxotrophic selection marker. DE plasmids were constructed by PCR-amplifying genes of interest to append 5’ and 3’ restriction sites using primer overhangs (Supplementary Data 1), digesting PCR products or synthesized gene fragments with appropriate pairs of restriction enzymes (SpeI, BamHI, EcoRI, PstI, and/or XhoI), and then ligating gene fragments into similarly digested vectors pAG414GPD-ccdB, pAG415GPD-ccdB, or pAG416GPD-ccdB^60^ using T4 DNA ligase (New England Biolabs, NEB).

YI plasmids comprised a gene of interest flanked by a constitutive promoter and terminator but lacked a yeast origin of replication or auxotrophic selection marker. YI plasmids were constructed by linearizing empty holding vectors pCS2656, pCS2657, pCS2658, pCS2661, or pCS2663 using ‘around-the-horn’ PCR with primers designed to bind at the 3’ and 5’ ends of the promoter and terminator, respectively (Supplementary Data 1). Genes of interest were PCR-amplified to append 5’ and 3’ overhangs with 35–40 bp of homology to the termini of the linearized vector backbones. Assembly of genes into YI vectors was performed using Gibson assembly. DE plasmids expressing GFP fusions of biosynthetic enzymes were prepared by first assembling PCR-amplified DNA fragments separately encoding GFP, the target enzyme, and a YI vector backbone using Gibson assembly, and subsequently subcloning the fusion constructs from YI plasmids into DE vectors using restriction enzymes and ligation cloning.

All PCR amplification was performed using Q5 DNA polymerase (NEB) and linear DNA was purified using the DNA Clean and Concentrator-5 kit (Zymo Research). Assembled plasmids were propagated in chemically competent *E. coli* (TOP10, Thermo Fisher Scientific) using heat-shock transformation and selection in Luria-Bertani (LB) broth or on LB-agar plates with either carbenicillin (100 μg/mL) or kanamycin (50 μg/mL) selection. Plasmid DNA was isolated by alkaline lysis from overnight *E. coli* cultures grown at 37 °C and 250 rpm in selective LB media using Econospin columns (Epoch Life Science) according to the manufacturer’s protocol. Plasmid sequences were verified by Sanger sequencing (Quintara Biosciences).

### Yeast strain construction

Strains used in this work (Supplementary Table 2) were derived from the parental strain CEN.PK2-1D^61^, referred to as CEN.PK2. Strains were grown non-selectively in yeast-peptone media supplemented with 2% w/v dextrose (YPD media), yeast nitrogen base (YNB) defined media (Becton, Dickinson and Company (BD)) supplemented with synthetic complete amino acid mixture (YNB-SC; Clontech) and 2% w/v dextrose, or on agar plates of the aforementioned media. Strains transformed with plasmids bearing auxotrophic selection markers (*URA3*, *TRP1*, *HIS3*, and/or *LEU2*) were grown selectively in YNB media supplemented with 2% w/v dextrose and the appropriate dropout solution (YNB-DO; Clontech) or on YNB-DO agar plates. Yeast strains derived from CSY1240 were deficient in acetate metabolism and were therefore grown on the aforementioned media supplemented with 0.1% w/v potassium acetate (i.e., YPAD or YNBA).

Yeast genomic modifications were performed using the CRISPRm method^62^. CRISPRm plasmids expressed *Streptococcus pyogenes* Cas9 and a single guide RNA (sgRNA) targeting a locus of interest in the yeast genome, and were constructed by assembly PCR and Gibson assembly of DNA fragments encoding *Sp*Cas9 (pCS3410), tRNA promoter and HDV ribozyme (pCS3411), a 20-nt guide RNA sequence, and tracrRNA and terminator (pCS3414) (Supplementary Data 2). For gene insertions, integration fragments comprising one or more genes of interest flanked by unique promoters and terminators were constructed using PCR amplification and cloned into holding vectors by Gibson assembly as described in Plasmid construction (Supplementary Fig. 1). Integration fragments were PCR-amplified using Q5 DNA polymerase (NEB) with flanking 40 bp microhomology regions to adjacent fragments and/or to the yeast genome at the integration site (Supplementary Data 1). For gene disruptions, integration fragments comprised 6–8 stop codons in all three reading frames flanked by 40 bp of microhomology to the disruption site, which was located within the first half of the open reading frame. For complete gene deletions, integration fragments comprised an auxotrophic marker gene flanked by 40 bp of microhomology to the deletion site. Approximately 300 ng of each integration fragment was co-transformed with 300 ng of CRISPRm plasmid targeting the desired genomic site. Positive integrants were identified by yeast colony PCR, Sanger sequencing, and/or functional screening by liquid chromatography and tandem mass spectrometry (LC-MS/MS).

### Yeast transformations

Yeast strains were chemically transformed using the Frozen-EZ Yeast Transformation II Kit (Zymo Research). Individual colonies were inoculated into YP(A)D media and grown overnight at 30 °C and 250 rpm. Saturated cultures were back-diluted between 1:10 and 1:50 in YP(A)D media and grown for an additional 5–7 h to reach exponential phase. Cultures were pelleted by centrifugation at 500 × *g* for 4 min and then washed twice by resuspending the pellet in 50 mM Tris-HCl buffer, pH 8.5. Washed pellets were resuspended in 20 μL of EZ2 solution per transformation and then mixed with 100–600 ng of total DNA and 200 μL of EZ3 solution. The yeast suspensions were incubated at 30 °C with gentle rotation for one hour. For plasmid transformations, the transformed yeast were directly plated onto YNB(A)-DO agar plates. For Cas9-mediated chromosomal modifications, yeast suspensions were instead mixed with 1 mL YP(A)D media, pelleted by centrifugation at 500 × *g* for 4 min, and then resuspended in 250 μL of fresh YP(A)D media. The suspensions were incubated at 30 °C with gentle rotation for an additional two hours to enable production of G418 resistance proteins and then spread onto YP(A)D plates containing 400 mg/L G418 (geneticin) sulfate. For all transformations, plates were incubated at 30 °C for 48–72 h before being used to inoculate cultures for metabolite assays.

### Spot dilution assays

Strains were inoculated into YNB(A)-DO media and grown overnight at 30 °C and 250 rpm. Saturated overnight cultures were pelleted by centrifugation at 500 × *g* for 4 min and resuspended in sterile Tris-HCl buffer, pH 8.0 to a concentration of 10^7^ cells/mL based on OD_600_. Ten-fold serial dilutions of each strain were prepared in Tris-HCl buffer and 10 μL of each dilution was spotted on pre-warmed YNB(A)-DO plates. Plates were incubated at 30 °C and imaged after 48 h.

### Growth conditions for metabolite assays

Small-scale metabolite production tests were conducted in YNB(A)-SC or YNB(A)-DO media in at least three replicates. Yeast colonies were inoculated into 300 μL of media and grown in 2 mL deep-well 96-well plates covered with AeraSeal gas-permeable film (Excel Scientific). Unless otherwise specified, cultures were grown for 48 h at 30 °C, 460 rpm, and 80% relative humidity in a Lab-Therm LX-T shaker (Adolf Kuhner).

### Growth conditions for media optimization

Strains were inoculated in triplicate into YPD media and grown overnight to saturation at 30 °C and 250 rpm in glass culture tubes. After 18 h of growth, cultures were back-diluted 10× into 300 μL of YNB-SC or YP media supplemented with corresponding carbon sources and grown in 2 mL deep-well 96-well plates covered with AeraSeal gas-permeable film (Excel Scientific) in triplicates for 72 h at 25 °C, 460 rpm, and 80% relative humidity in a Lab-Therm LX-T shaker (Adolf Kuhner).

### Growth conditions for time courses

For conventional low-density batch cultures, strains were inoculated in triplicate into YP(A)D media and grown overnight to saturation at 30 °C and 250 rpm. Overnight cultures were back-diluted to OD_600_ = 0.5 (for acetate-auxotrophic strains) or 0.1 (for acetate-prototrophic strains) in YNB(A)-SC media supplemented with appropriate carbon sources and grown in 50-mL shake flasks with 10 mL starting volume in triplicates at 30 °C or 25 °C and 300 rpm for 96–144 h. To simulate high-density batch culture conditions, strains were inoculated in triplicate into 10 mL of YPD media and grown overnight to saturation at 30 °C and 250 rpm. Saturated cultures were pelleted by centrifugation at 500 × *g* for 4 min and 3000 × *g* for 1 min and then resuspended in 10 mL of fresh YNB-SC or YP media supplemented with appropriate carbon sources and grown in 50-mL shake flasks with 10 mL starting volume in triplicates at 25 °C and 300 rpm for 144 h. Where indicated, fed-batch conditions were approximated by supplementing cultures after 72 h of growth with appropriate carbon sources and amino acids at 2% and 1X final concentrations, respectively. At appropriate time points, 250 μL samples were removed from cultures for analysis; 100 μL of culture was diluted 10× and used for optical density measurement at 600 nm on a Nanodrop 2000c spectrophotometer, and 150 μL of culture was used for metabolite quantification.

### Analysis of metabolite production

Cultures were pelleted by centrifugation at 3500 × *g* for 5 min at 12 °C and 100–200 μL aliquots of the supernatant were removed for direct analysis. Metabolite production was analyzed by LC-MS/MS using an Agilent 1260 Infinity Binary HPLC and an Agilent 6420 Triple Quadrupole mass spectrometer. Chromatography was performed using a Zorbax EclipsePlus C18 column (2.1 × 50 mm, 1.8 μm; Agilent Technologies) with 0.1% v/v formic acid in water as mobile phase solvent A and 0.1% v/v formic acid in acetonitrile as solvent B. The column was operated with a constant flow rate of 0.4 mL/min at 40 °C and a sample injection volume of 5 μL. Compound separation was performed using the following gradient:^45^ 0.00–0.75 min, 1% B; 0.75–1.33 min, 1–25% B; 1.33–2.70 min, 25–40% B; 2.70–3.70 min, 40–60% B; 3.70–3.71 min, 60–95% B; 3.71–4.33 min, 95% B; 4.33–4.34 min, 95–1% B; 4.34–5.00 min, equilibration with 1% B. The LC eluent was directed to the MS from 0.01–5 min operating with electrospray ionization (ESI) in positive mode, source gas temperature 350 °C, gas flow rate 11 L/min, and nebulizer pressure 40 psi. Metabolites were quantified by integrated peak area in MassHunter Workstation software (Agilent) based on the multiple reaction monitoring (MRM) parameters in Supplementary Table 3 and standard curves (Supplementary Fig. 19). Primary MRM transitions were identified by analysis of 0.1–1 mM aqueous standards using the MassHunter Optimizer software package (Agilent) and corroborated against published mass transitions if available, and/or against predicted transitions determined using the CFM-ID fragment prediction utility^63^ and the METLIN database^64^.

### Fluorescence microscopy

Individual colonies of yeast strains transformed with plasmids encoding biosynthetic enzymes fused to fluorescent protein reporters were inoculated into 1 mL YNB-DO media and grown overnight at 30 °C and 250 rpm. Overnight cultures were pelleted by centrifugation at 500 × *g* for 4 min and resuspended in 2 mL YNB-DO media with 2% w/v dextrose and then grown at 30 °C and 250 rpm for an additional 4–6 h to reach exponential phase and allow expressed fluorescent proteins to fold completely. Approximately 5–10 μL of culture was spotted onto a glass microscope slide and covered with a glass coverslip (Thermo Fisher Scientific) and then imaged using a Nikon TE2000 inverted microscope with a ×60 oil immersion objective. Fluorescence excitation was performed using a LambdaXL xenon arc lamp (Sutter Instrument Company) and the following filter settings: GFP, ET470/40X excitation filter and ET525/50 emission filter; mCherry, ET572/35X excitation filter and ET632/60 emission filter. Emitted light was captured with a CoolSNAP HQ2 CCD camera (Photometrics Scientific) and Micro-Manager software, and subsequent image analysis was performed in ImageJ (NIH). Images were converted to pseudocolor using the ‘Merge Channels’ and ‘Split Channels’ functions in ImageJ (Image → Color → Merge/Split Channels). Histogram stretching was applied equally across all images for a given channel (bright field, GFP, or mCherry) to improve contrast.

### Identification of orthologs from transcriptome databases

Orthologs of *N. tabacum N-*methylputrescine oxidase (*NtMPO1*) were identified using a tBLASTn search of the transcriptomes of *D. metel* and *A. belladonna* in the 1000 Plants Project database^65^. This search yielded only one unique sequence from each of the two transcriptomes that was full-length (i.e., within 10% of the length of the query sequence, 790 residues) and with expectation value 0.0: JNVS_scaffold_2009311 (*D. metel*) and BOLZ_scaffold_2171654 (*A. belladonna*). Coding sequences for these two putative genes were optimized for yeast expression and then cloned into expression vectors as described in the section on plasmid construction above.

### Enzyme structural analysis via homology modeling

Heterologous enzymes were analyzed for structural features that may prove problematic during expression in yeast, such as large unstructured regions, by examining homology models constructed using RaptorX with default modeling parameters^66^. Resultant protein models were visualized using PyMOL (Schrodinger).

### Statistics

Where indicated, the statistical significance of any differences in metabolite titer between conditions was verified using Student’s two-tailed *t*-test. Biological replicates are defined as independent cultures inoculated from separate yeast colonies or streaks and cultivated in separate containers.

### Reporting summary

Further information on research design is available in the Nature Research Reporting Summary linked to this article.

## Supplementary information

Supplementary Information

Peer Review

Reporting Summary

Description of Additional Supplementary Files

Supplementary Data 1

Supplementary Data 2

Source Data

## Data Availability

Data supporting the findings of this work are available within the paper and its Supplementary Information files. A reporting summary for this Article is available as a Supplementary Information file. The datasets generated and analyzed during the current study are available from the corresponding author upon request. The source data underlying Figs. 2b, c, e–f, 3c, d, and 4b–f, as well as Supplementary Figs. 2, 4, 5c, 6b, d, 7, 8, 9b, 11b, c, 12, 13, 14, and 19 are provided as a Source Data file.
