# Supplementary material for: Engineering a microbial biosynthesis platform for de novo production of tropane alkaloids
Source: Nat Commun. 2019 Aug 12;10:3634. doi: 10.1038/s41467-019-11588-w (PMC6690885; doi:10.1038/s41467-019-11588-w)
Supplement: Supplementary file 1 — Supplementary Information [file 41467_2019_11588_MOESM1_ESM.pdf]

Engineering a microbial biosynthesis platform for *de novo* production of tropane alkaloids

Prashanth Srinivasan and Christina D. Smolke

Supplementary Table 1. Genes used in this study.

Gene ID	Description	Source organism	Accession
ARG2	Glutamate *N*-acetyltransferase	*Saccharomyces cerevisiae*	P40360
CAR1	Arginase	*Saccharomyces cerevisiae*	P00812
FMS1	Polyamine oxidase	*Saccharomyces cerevisiae*	P50264
ORT1	Mitochondrial ornithine transporter	*Saccharomyces cerevisiae*	Q12375
SPE1	Ornithine decarboxylase	*Saccharomyces cerevisiae*	P08432
NCP1	NADP-cytochrome P450 reductase	*Saccharomyces cerevisiae*	P16603
AbCYP82M3	Tropinone synthase	*Atropa belladonna*	AYU65303.1
AbMPO1	Putative *N*-methylputrescine oxidase	*Atropa belladonna*	*This study*
AbPMT1	Putrescine *N*-methyltransferase	*Atropa belladonna*	Q9S7W8
AbPYKS	Pyrrolidine ketide synthase	*Atropa belladonna*	AYU65302.1
AsADC	Arginine decarboxylase	*Avena sativa*	P22220
AtAIH	Agmatine deiminase/iminohydrolase	*Arabidopsis thaliana*	Q8GWW7
AtARGAH2	Arginase	*Arabidopsis thaliana*	Q9ZPF5
AtATR1	NADP-cytochrome P450 reductase 1	*Arabidopsis thaliana*	Q9SB48
AtCPA	*N*-carbamoylputrescine amidase	*Arabidopsis thaliana*	Q8VYF5
AtPAL1	Phenylalanine-ammonia lyase 1	*Arabidopsis thaliana*	P35510
At4CL5	4-coumarate-CoA ligase	*Arabidopsis thaliana*	Q9LU36
DmMPO1	Putative *N*-methyltransferase oxidase	*Datura metel*	*This study*
DsTR1	Tropinone reductase I	*Datura stramonium*	P50162
DsPMT1	Putrescine *N*-methyltransferase	*Datura stramonium*	Q70EW6
EcCPR	NADP-cytochrome P450 reductase 1	*Eschscholzia californica*	O24425
EcCS	Cocaine synthase	*Erythroxylum coca*	A0A059Q4T4
NtMPO1	*N*-methylputrescine oxidase	*Nicotiana tabacum*	A4GZ88
PsCPR	NADP-cytochrome P450 reductase 1	*Papaver somniferum*	O24424
SlCPA	*N*-carbamoylputrescine amidase	*Solanum lycopersicum*	Q9XGI9
speB	Agmatinase	*Escherichia coli*	P60651

Supplementary Table 2. Yeast strains used in this study.

Strain	Genotype
CEN.PK2-1D	MATα *ura3-52*; *trp1-289*; *leu2-3/112*; *his3Δ1*; *MAL2-8C*; *SUC2*
CSY1225	CEN.PK2-1D; pCS4196, pCS4211, pCS4216
CSY1226	CEN.PK2-1D; pCS4223, pCS4225
CSY1227	CEN.PK2-1D; pCS4196, pCS4216, pCS4239
CSY1229	CEN.PK2-1D; *meu1-14*
CSY1230	CEN.PK2-1D; *oaz1-16*
CSY1231	CEN.PK2-1D; *spe4-20*
CSY1232	CEN.PK2-1D; *agp2-35*
CSY1233	CEN.PK2-1D; *sky1-29*
CSY1234	CEN.PK2-1D; *meu1-14*; *oaz1-16*
CSY1235	CSY1234; *his2*:: P_{TPI1}-*ARG2*-T_{STE2}, P_{PGK1}-*CAR1*-T_{PHO5}, P_{TDH3}-*FMS1*-T_{ADH1}, pCS4239
CSY1236	CSY1235; *hfd1-45*, pCS4239
CSY1237	CSY1235; *ald4-48*, pCS4239
CSY1238	CSY1235; *ald5-53*, pCS4239
CSY1239	CSY1235; *ald6-55*, pCS4239
CSY1240	CSY1235; *hfd1-45*; *ald4-48*; *ald5-53*; *ald6-55*, pCS4239
CSY1241	CSY1240; *ALD2Δ0-ALD3Δ0*:: *HIS3*, pCS4239
CSY1242	CSY1241; *trp1*:: P_{PGK1}-*AsADC*-T_{ADH1}, P_{TEF1}-*SPE1*-T_{CYC1}, P_{TDH3}-*speB*-T_{PHO5}
CSY1243	CSY1242; *ura3*:: P_{PGK1}-*AbPMT1*-T_{PHO5}, P_{TDH3}-*DmMPO1*$^{\Delta C\text{-}PTS1}$-T_{ADH1}
CSY1246	CSY1242; *ura3*:: P_{PGK1}-*AbPMT1*-T_{PHO5}, P_{TDH3}-*DmMPO1*$^{\Delta C\text{-}PTS1}$-T_{ADH1}, P_{TEF1}-*DsTR1*-T_{CYC1}
CSY1247	CSY1246; pCS4200, pCS4246, pCS4247
CSY1248	CSY1246; *leu2*:: P_{PGK1}-*AbCYP82M3*-T_{PHO5}, P_{TEF1}-*AtATR1*-T_{CYC1}, P_{HXT7}-*AbPYKS*-T_{PGK1}
CSY1249	CSY1248; *ald6*-55:: *ALD6*
CSY1251	CSY1249; *pad1*:: P_{PGK1}-*AbPMT1*-T_{PHO5}, P_{TDH3}-*DsPMT1*-T_{CYC1}, P_{HXT7}-*AbPYKS*-T_{PGK1}
CSY1282	CSY1251; pCS4207, pCS4252

Supplementary Table 3. LC-MS/MS multiple reaction monitoring parameters.

Compound	MRM* transition (m/z+)	Fragmentor	Collision energy	Reference
Cinnamoyltropine	272 → 124	120	25	[1]
4-(1-methyl-2-pyrrolidinyl)-3-oxobutanoic acid	186 → 84	74	13	[2]
Tropine	142 → 98	50	21	[3]
Hygrine	142 → 84	74	13	[4]
Tropinone	140 → 98	50	21	[3]
N-carbamoylputrescine	132 → 72	94	17	This study
Agmatine	131 → 72	94	17	This study
4-methylaminobutyric acid	118 → 87	63	13	This study
N-methylputrescine	103 → 72	20	9	[3]
4-methylaminobutanal	102 → 71	20	9	This study
Putrescine	89 → 72	18	9	[3]
N-methylpyrrolinium	84 → 57	50	35	This study

* MRM: multiple reaction monitoring.

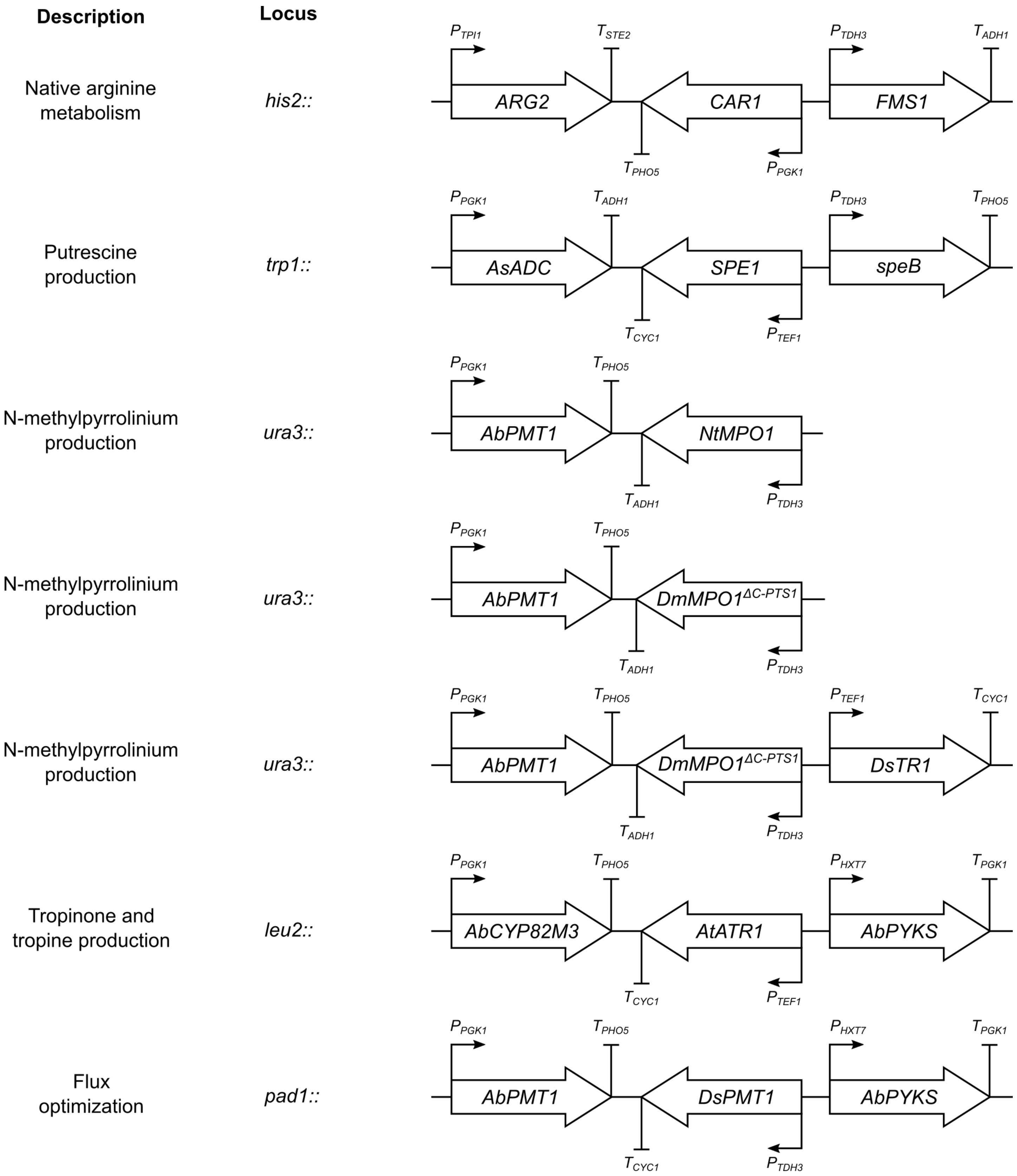

Supplementary Figure 1. Design of genomic integrations for pathway construction in yeast. Block arrows represent gene expression cassettes with unique promoter and terminator for each locus. Genus and species sources for heterologous genes are indicated by two letters preceding the gene symbol. Superscript annotations on gene symbols indicate N- or C-terminal modifications. Refer to Supplementary Table 1 for gene sources and Supplementary Table 2 for strain genotypes.

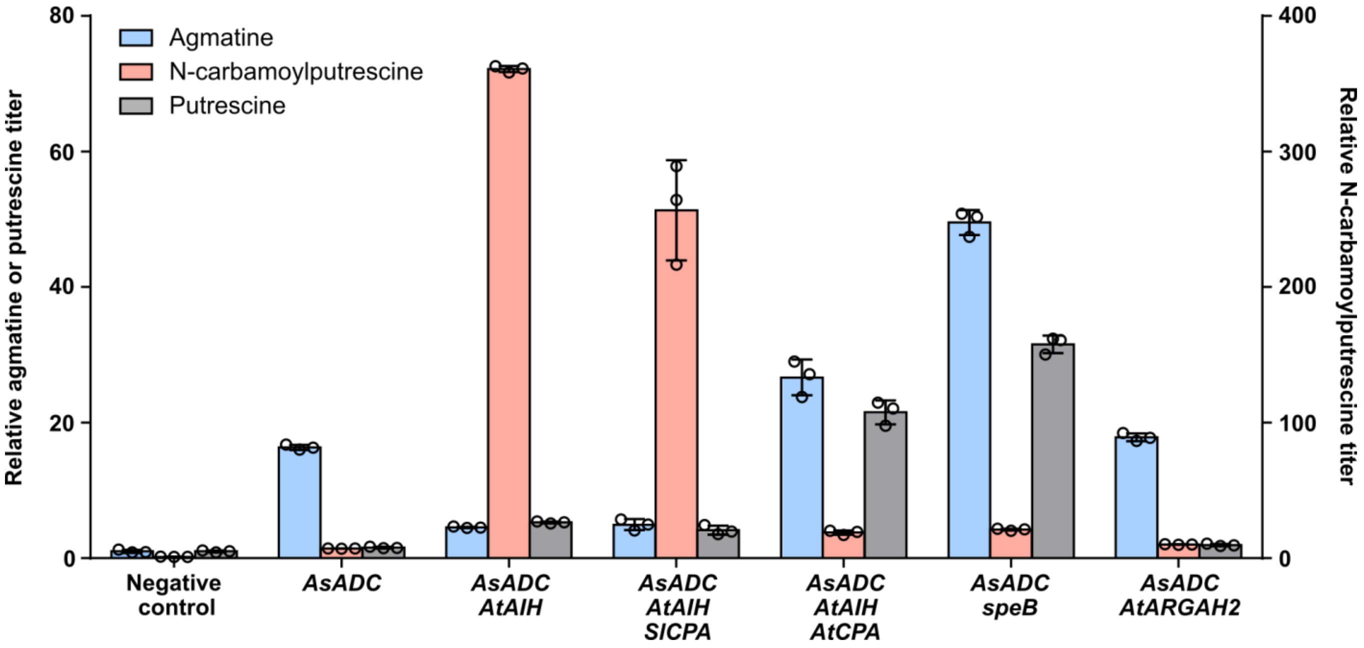

Supplementary Figure 2. Functional validation of agmatine/putrescine biosynthetic pathway genes in yeast. Wild-type yeast strain CEN.PK2 was transformed with three low-copy plasmids to co-express between zero (negative control) and three of the indicated biosynthetic genes. Plasmids expressing blue fluorescent protein (BFP) were used as negative controls for each of the three auxotrophic selection markers *URA3*, *TRP1*, and *LEU2* (pCS4208, 4212, 4213). Transformed strains were cultured in selective media (YNB-DO) with 2% dextrose at 30 °C for 48 h prior to LC-MS/MS analysis of metabolite production. All data show titers as measured by LC-MS/MS peak area relative to the negative control (CEN.PK2). Data represent the mean of n = 3 biologically independent samples (open circles) and error bars show standard deviation. Source data are provided as a source data file.

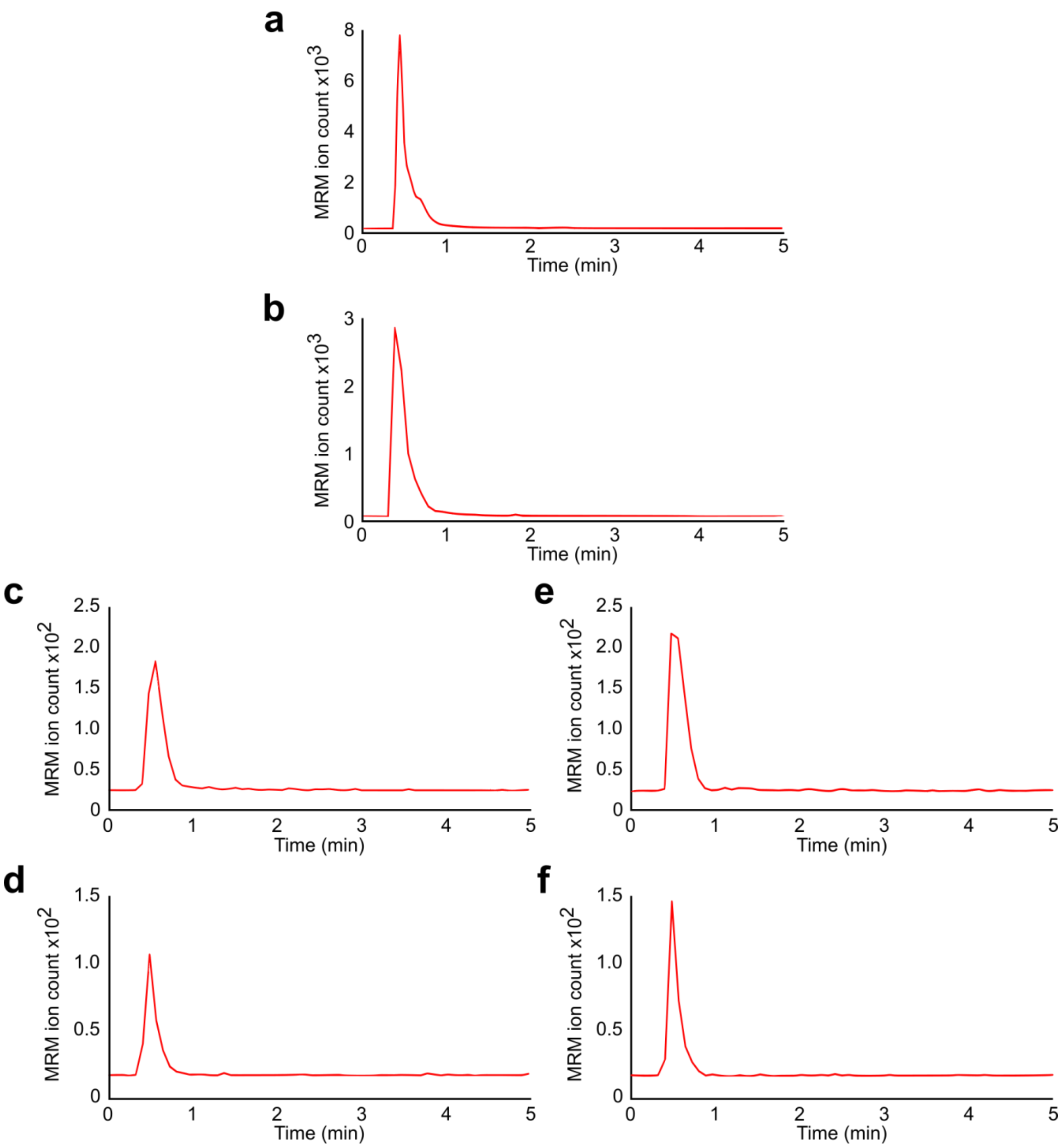

Supplementary Figure 3. LC-MS/MS multiple reaction monitoring chromatograms for detection of intermediates between putrescine and NMPy in CSY1235. (**a**) Multiple reaction monitoring (MRM) chromatogram of putrescine (m/z^+ 89 → 72) for CSY1235 harboring pCS4239 for putrescine overproduction. (**b**) MRM chromatogram of NMP (m/z^+ 103 → 72) for CSY1235 harboring pCS4239 and expressing *AbPMT1* from a low-copy plasmid. (**c,d**) MRM chromatograms of 4MAB (m/z^+ 102 → 71) and NMPy (m/z^+ 84 → 57), respectively, for CSY1235 harboring pCS4239 and expressing *AbPMT1* and *NtMPO1* from low-copy plasmids. (**e,f**) MRM chromatograms of 4MAB (m/z^+ 102 → 71) and NMPy (m/z^+ 84 → 57), respectively, for CSY1235 harboring pCS4239 and expressing *AbPMT1* and *DmMPO1*$^{\Delta C\text{-}PTS1}$ from low-copy plasmids. Y-axes of traces are raw MRM ion counts. All chromatograms were generated by LC-MS/MS analysis of the extracellular medium after 48 hours of growth at 30°C in selective media (YNB-DO) with 2% dextrose. Traces are representative of at least three biological replicates.

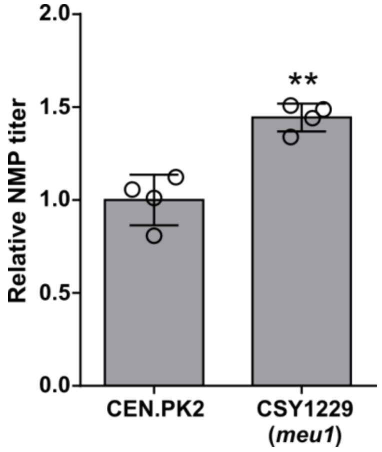

Supplementary Figure 4. Effect of *MEU1* disruption on SAM-dependent putrescine *N*-methylation by *AbPMT1*. Wild-type strain CEN.PK2 or *meu1* disruption strain CSY1229 were co-transformed with low-copy plasmids expressing *SPE1*, *AsADC*, and *speB* (pCS4239) and *AbPMT1* (pCS4193). Data indicate mean NMP titer relative to CEN.PK2 control as quantified by LC-MS/MS peak area for n = 4 biologically independent samples (open circles) after 48 hours of growth at 30 °C in selective media (YNB-DO) with 2% dextrose. Error bars show standard deviation. Student's two-tailed t-test: *P < 0.05, ** P < 0.01, *** P < 0.001. Source data are provided as a source data file.

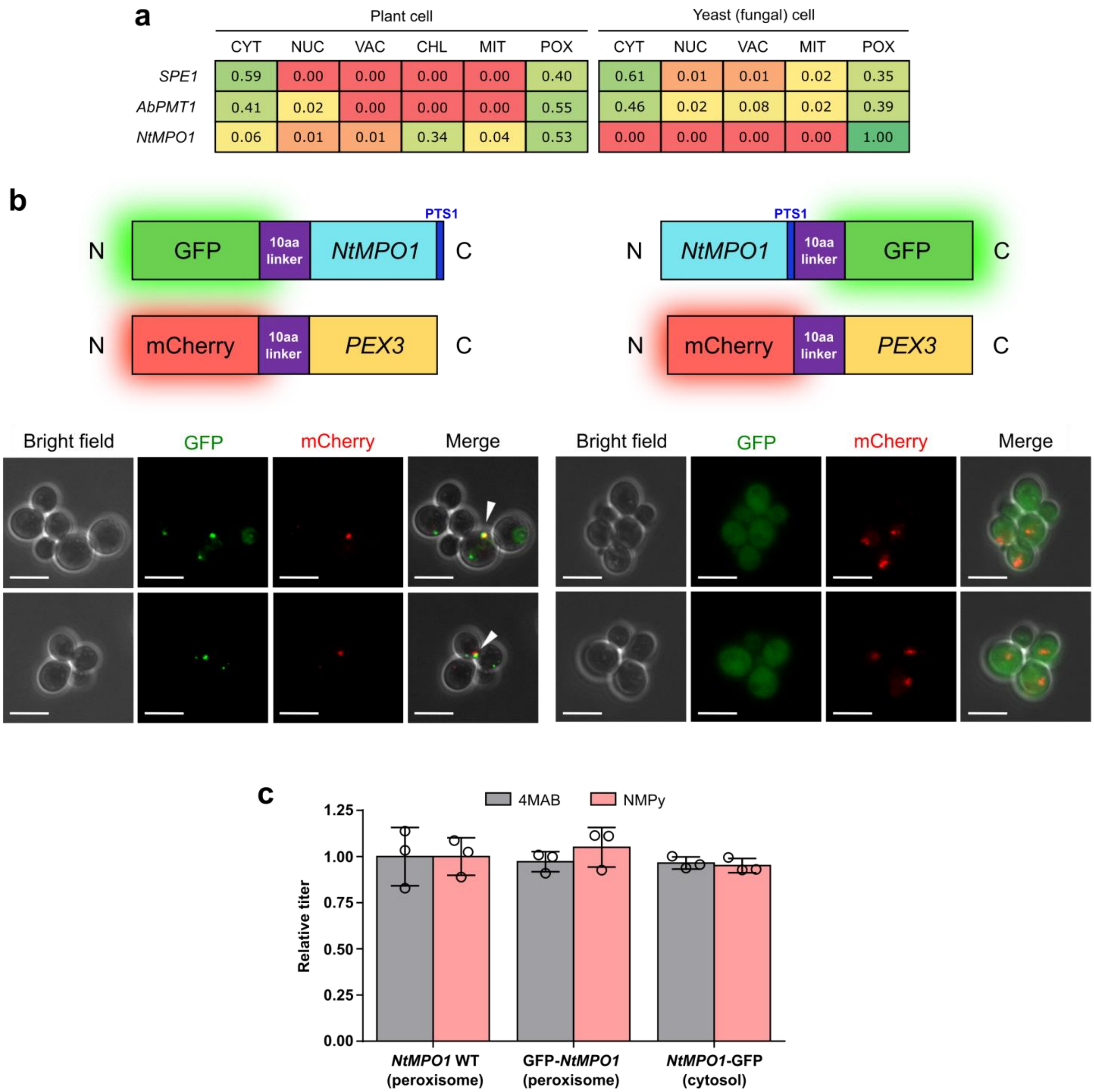

Supplementary Figure 5. Localization of MPO1 from *Nicotiana tabacum* to yeast peroxisomes. (**a**) *In silico* prediction of subcellular localization for NMPy biosynthetic genes in plant and yeast/fungal cells using the SherLoc2 web server. Values and coloring indicate probability scores (0 to 1) for localization to each compartment: CYT, cytosol; NUC, nucleus; VAC, vacuole; CHL, chloroplast; MIT, mitochondria; POX, peroxisome. (**b**) Fluorescence microscopy of *NtMPO1* N- and C-terminal GFP fusions co-expressed with peroxisome marker mCherry-PEX3 in wild-type yeast (CEN.PK2). White arrows indicate colocalization of GFP-tagged *NtMPO1* with peroxisomes. Scale bar, 10 µm. (**c**) Effect of forcing cytosolic localization of *NtMPO1* on 4MAB or NMPy production. Wild-type yeast (CEN.PK2) was co-transformed with low-copy plasmids expressing wild-type *NtMPO1* or N- or C-terminal GFP fusions together with low-copy plasmids expressing *SPE1*, *AsADC*, and *speB* (pCS4239) and *AbPMT1* (pCS4193). LC-MS/MS analysis was performed after 48 hours of growth at 30 ^{o}C in selective media (YNB-DO) with 2% dextrose. Data represent mean of n = 3 biologically independent samples (open circles); error bars show standard deviation. Most probable sub-cellular compartment is indicated based on microscopy data in (b). Source data of Supplementary Figure 5c are provided as a source data file.

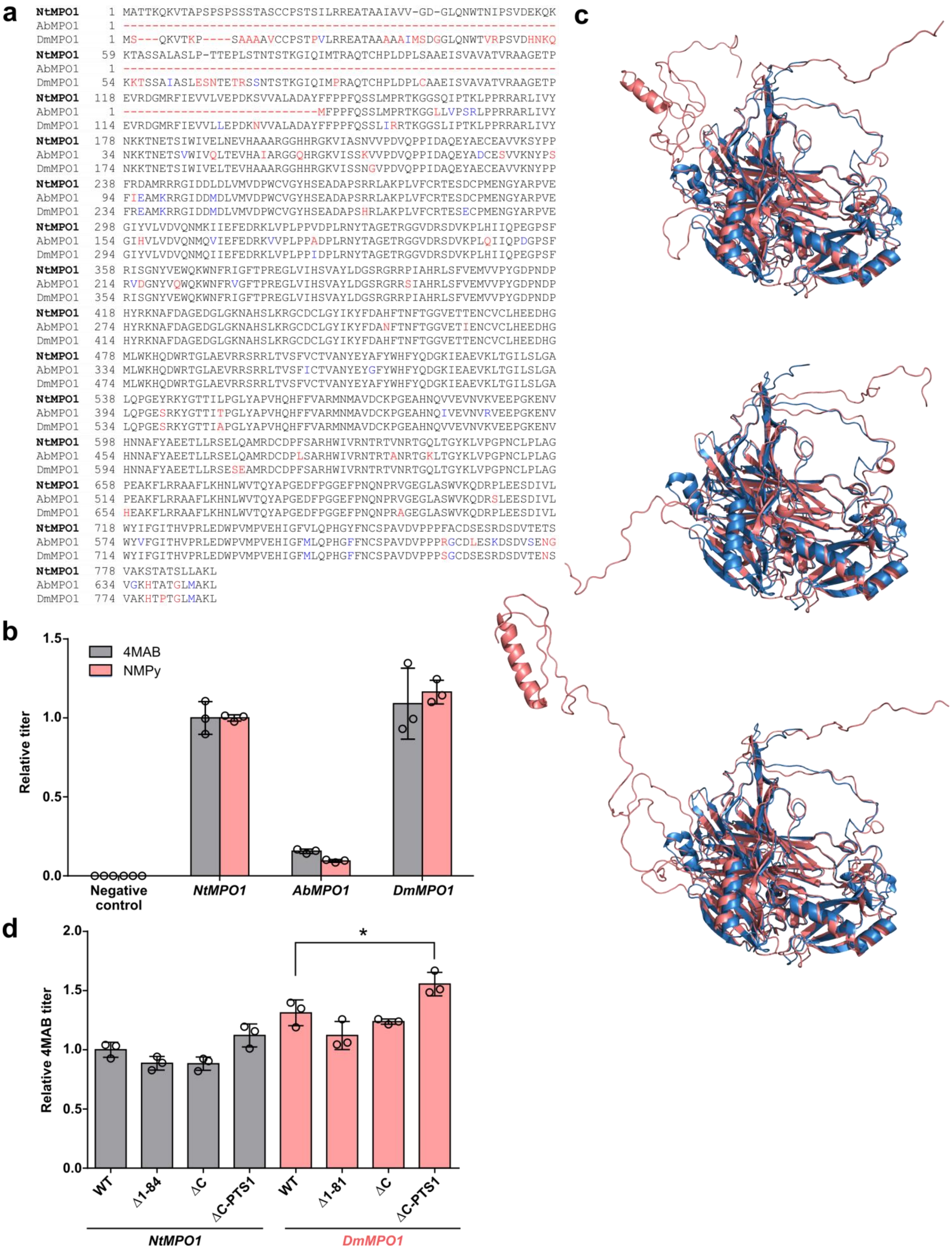
a
NtMPO1 1 MATTKQKVTAPSPSPSSSTASCCPSTSILRREATAAIAVV-GD-GLQNWTNIPSVDEKQK
AbMPO1 1 --
DmMPO1 1 MS---QKVTKP----SAAAAVCCPSTPVLRREATAAAAIMSDGGLQNWTVRPSVDHNKQ
NtMPO1 59 KTASSALASLP-TTEPLSTNTSTKGIQIMTRAQTCHPLDPLSAAEISVAVATVRAAGETP
AbMPO1 1 --
DmMPO1 54 KKTSSAIASLESNTETRSSNTSTKGIQIMPRAQTCHPLDPLCAAEISVAVATVRAAGETP
NtMPO1 118 EVRDGMRFIEVVLVEPDKSVVALADAYFFPPFQSSLMPRTKGGSQIPTKLPPRRARLIVY
AbMPO1 1 ----------------------------MFPPFQSSLMPRTKGGLLVPSRLPPRRARLIVY
DmMPO1 114 EVRDGMRFIEVVLLEPDKNVVALADAYFFPPFQSSLIRRTKGGSLIPTKLPPRRARLIVY
NtMPO1 178 NKKTNETSIWIVELNEVHAAARGGHHRGKVIASNVVPDVQPPIDAQEYAECEAVVKSYPP
AbMPO1 34 NKKTNETSVWIVQLTEVHAIARGGQHRGKVISSKVVPDVQPPIDAQEYADCESVVKNYPS
DmMPO1 174 NKKTNETSIWIVELTEVHAAARGGHHRGKVISSNGVPDVQPPIDAQEYAECEAVVKNYPP
NtMPO1 238 FRDAMRRRGIDDLDLVMVDPWCVGYHSEADAPSRRLAKPLVFCRTESDCPMENGYARPVE
AbMPO1 94 FIEAMKRRGIDDMDLVMVDPWCVGYHSEADAPSRRLAKPLVFCRTESDCPMENGYARPVE
DmMPO1 234 FREAMKRRGIDDMDLVMVDPWCVGYHSEADAPSHRLAKPLVFCRTESECPMENGYARPVE
NtMPO1 298 GIYVLVDVQNMKIIEFEDRKLVPLPPVDPLRNYTAGETRGGVDRSDVKPLHIIQPEGPSF
AbMPO1 154 GIHVLVDVQNMQVIEFEDRKVVPLPPADPLRNYTAGETRGGVDRSDVKPLQIIQPDGPSF
DmMPO1 294 GIYVLVDVQNMQIIEFEDRKLVPLPPIDPLRNYTAGETRGGVDRSDVKPLHIIQPEGPSF
NtMPO1 358 RISGNYVEWQKWNFRIGFTPREGLVIHSVAYLDGSRGRRPIAHRLSFVEMVVPYGDPNDP
AbMPO1 214 RVDGNYVQWQKWNFRVGFTPREGLVIHSVAYLDGSRGRRSIAHRLSFVEMVVPYGDPNDP
DmMPO1 354 RISGNYVEWQKWNFRIGFTPREGLVIHSVAYLDGSRGRRPIAHRLSFVEMVVPYGDPNDP
NtMPO1 418 HYRKNAFDAGEDGLGKNAHSLKRGCDCLGYIKYFDAHFTNFTGGVETTENCVCLHEEDHG
AbMPO1 274 HYRKNAFDAGEDGLGKNAHSLKRGCDCLGYIKYFDANFTNFTGGVETIENCVCLHEEDHG
DmMPO1 414 HYRKNAFDAGEDGLGKNAHSLKRGCDCLGYIKYFDAHFTNFTGGVETTENCVCLHEEDHG
NtMPO1 478 MLWKHQDWRTGLAEVRRSRRLTVSFVCTVANYEYAFYWHFYQDGKIEAEVKLTGILSLGA
AbMPO1 334 MLWKHQDWRTGLAEVRRSRRLTVSFICTVANYEYGFYWHFYQDGKIEAEVKLTGILSLGA
DmMPO1 474 MLWKHQDWRTGLAEVRRSRRLTVSFVCTVANYEYAFYWHFYQDGKIEAEVKLTGILSLGA
NtMPO1 538 LQPGEYRKYGTTILPGLYAPVHQHFFVARMNMAVDCKPGEAHNQVVEVNVKVEEPGKENV
AbMPO1 394 LQPGESRKYGTTITPGLYAPVHQHFFVARMNMAVDCKPGEAHNQIVEVNVRVEEPGKENV
DmMPO1 534 LQPGESRKYGTTIAPGLYAPVHQHFFVARMNMAVDCKPGEAHNQVVEVNVKVEEPGKENV
NtMPO1 598 HNNAFYAEETLLRSELQAMRDCDPFSARHWIVRNTRTVNRTGQLTGYKLVPGPNCLPLAG
AbMPO1 454 HNNAFYAEETLLRSELQAMRDCDPLSARHWIVRNTRTANRTGKLTGYKLVPGPNCLPLAG
DmMPO1 594 HNNAFYAEETLLRSESEAMRDCDPFSARHWIVRNTRTVNRTGQLTGYKLVPGPNCLPLAG
NtMPO1 658 PEAKFLRRAAFLKHNLWVTQYAPGEDFPGGEFPNQNPRVGEGLASWVKQDRPLEESDIVL
AbMPO1 514 PEAKFLRRAAFLKHNLWVTQYAPGEDFPGGEFPNQNPRVGEGLASWVKQDRSLEESDIVL
DmMPO1 654 HEAKFLRRAAFLKHNLWVTQYAPGEDFPGGEFPNQNPRAGEGLASWVKQDRPLEESDIVL
NtMPO1 718 WYIFGITHVPRLEDWPVMPVEHIGFVLQPHGYFNCSPAVDVPPPFACDSESRDSDVTETS
AbMPO1 574 WYVFGITHVPRLEDWPVMPVEHIGFMLQPHGFFNCSPAVDVPPPRGCDLESKDSDVSENG
DmMPO1 714 WYIFGITHVPRLEDWPVMPVEHIGFMLQPHGFFNCSPAVDVPPPSGCDSESRDSDVTENS
NtMPO1 778 VAKSTATSLLAKL
AbMPO1 634 VGKHTATGLMAKL
DmMPO1 774 VAKHTPTGLMAKL
b
4MAB
NMPy
Relative titer
1.5
1.0
0.5
0.0
Negative control
NtMPO1
AbMPO1
DmMPO1
c
d
*
Relative 4MAB titer
2.0
1.5
1.0
0.5
0.0
WT
Δ1-84
ΔC
ΔC-PTS1
WT
Δ1-81
ΔC
ΔC-PTS1
NtMPO1
DmMPO1

Supplementary Figure 6. Identification of novel *N*-methylputrescine oxidase variants from *Solanaceae* transcriptomes. MPO, *N*-methylputrescine oxidase. (**a**) Alignment of query *NtMPO1* sequence against *AbMPO1* and *DmMPO1* candidates from 1000 Plants Project database. Blue indicates conservation of amino acid structure; red indicates mismatches. (**b**) Comparison of relative activities of MPO orthologs. Strain CSY1235 was co-transformed with low-copy plasmids expressing *SPE1*, *AsADC*, and *speB* (pCS4239), *AbPMT1* (pCS4193), and one of the three MPO variants. (**c**) Homology models of MPO enzymes (pink) constructed based on the crystal structure of *Pisum sativum* copper-containing amino oxidase (PDB: 1KSI, blue) using the RaptorX web server. Top: *NtMPO1*; center: *AbMPO1*; bottom: *DmMPO1*. (**d**) Comparison of 4MAB production in engineered strains expressing methylputrescine oxidase N- or C-terminal truncations. Wild-type (WT) enzymes and indicated truncations were expressed from low-copy plasmids in CSY1235. For (b) and (d), LC-MS/MS analysis was performed after 48 hours of growth in selective media at 30 ^{o}C. Data represent mean of n = 3 biologically independent samples (open circles); error bars show standard deviation. Student's two-tailed t-test: *P < 0.05, ** P < 0.01, *** P < 0.001. Source data of Supplementary Figure 6b and 6d are provided as a source data file.

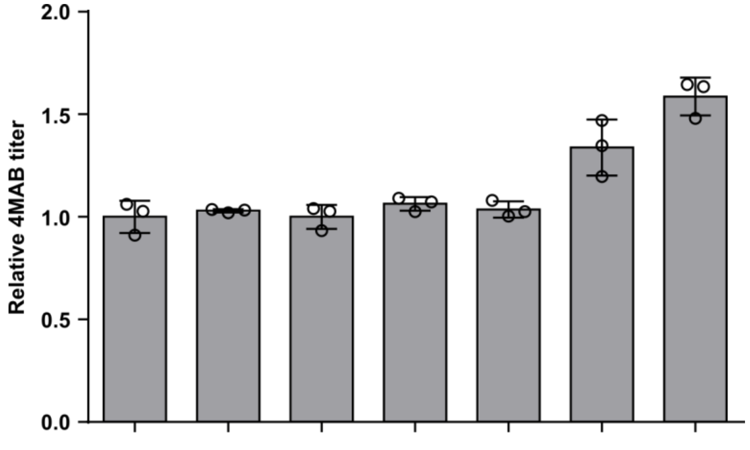

CSY#	1235	1236	1237	1238	1239	1240	1241
HFD1	+	−	+	+	+	−	−
ALD2	+	+	+	+	+	+	−
ALD3	+	+	+	+	+	+	−
ALD4	+	+	−	+	+	−	−
ALD5	+	+	+	−	+	−	−
ALD6	+	+	+	+	−	−	−

Supplementary Figure 7. Effect of aldehyde dehydrogenase disruptions on production of 4MAB in engineered yeast. Plus and minus symbols indicate presence or absence of functional enzyme, respectively. Strains were co-transformed with low-copy plasmids expressing *SPE1*, *AsADC*, and *speB* (pCS4239), *AbPMT1* (pCS4193), and *DmMPO1*$^{\Delta C\text{-}PTS1}$ (pCS4238) and were cultured in selective (YNB-DO) media with 2% dextrose at 30 °C for 48 h before LC-MS/MS analysis. Data represent the mean of n = 3 biologically independent samples (open circles) and error bars show standard deviation. Source data are provided as a source data file.

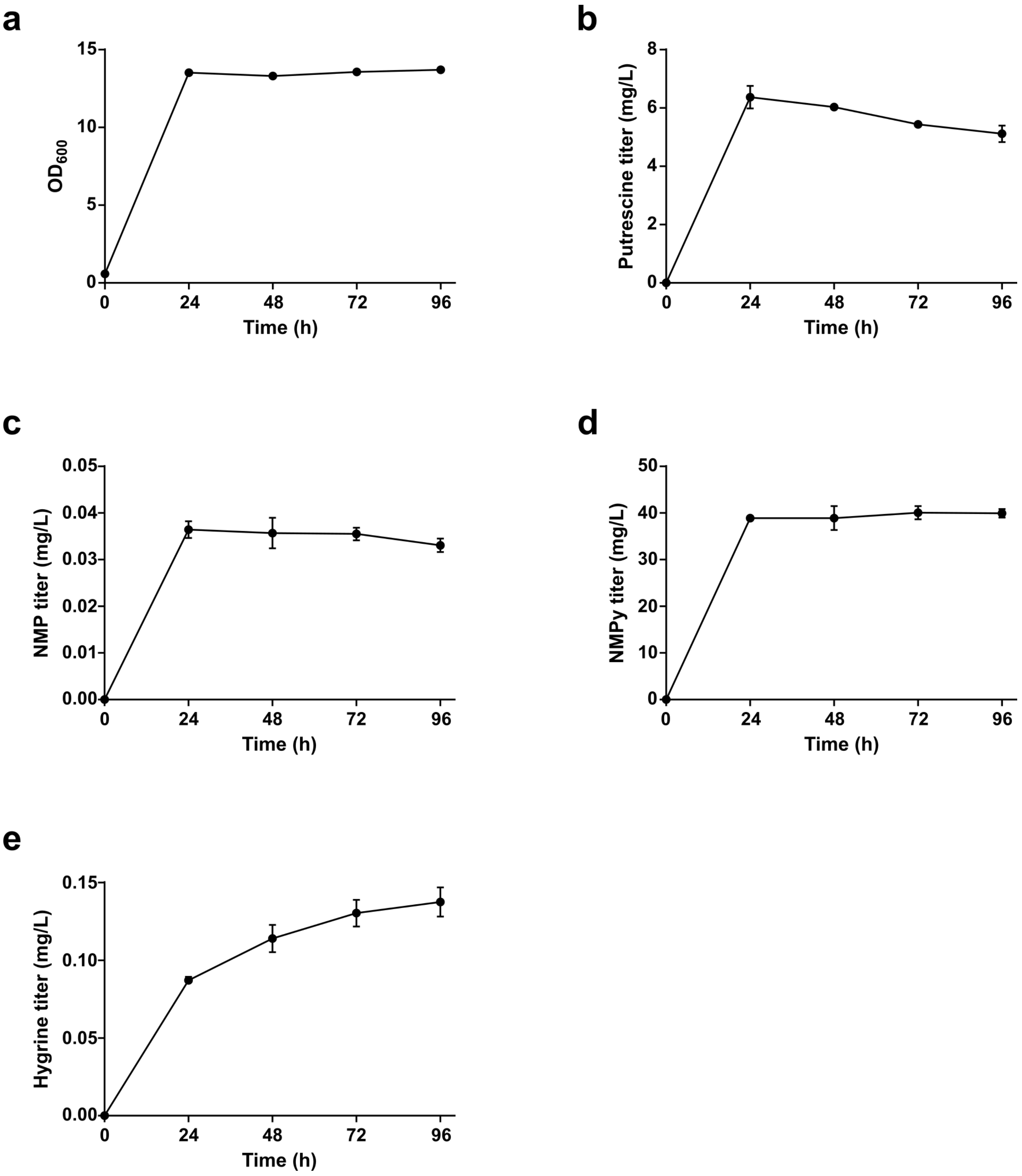

Supplementary Figure 8. Production of TA precursors in shake-flask cultures of the NMPy-producing strain CSY1243 over 96 hours of growth. Cultures were grown in non-selective media (YNBA-SC) at 30 °C and 300 rpm. Metabolite titers in the growth medium were quantified by LC-MS/MS analysis and optical density (OD_{600}) of diluted culture aliquots was measured at 600 nm. Data indicate the mean of n = 3 biologically independent samples and error bars show standard deviation. Source data are provided as a source data file.

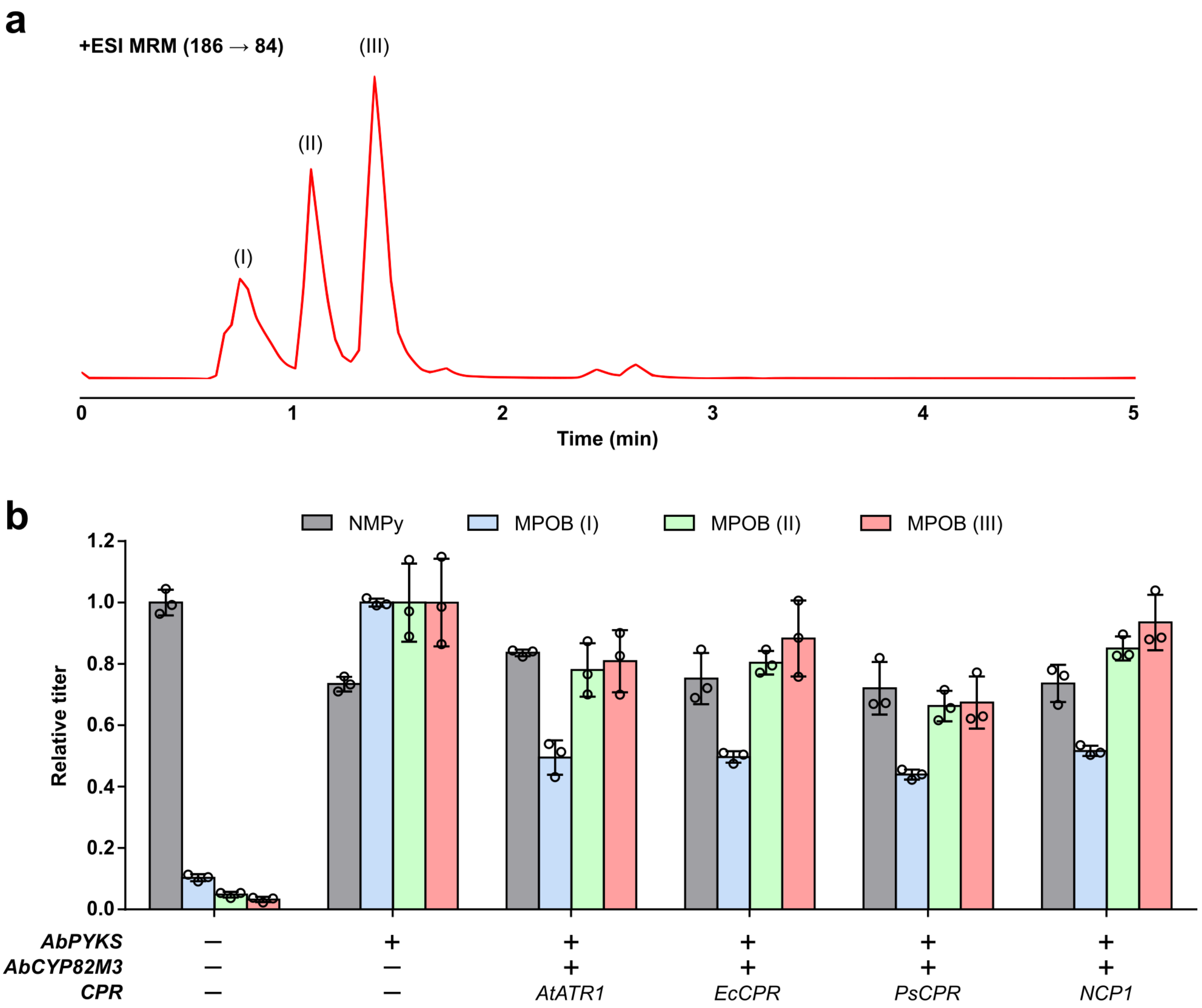

Supplementary Figure 9. Accumulation of NMPy and MPOB in the media of CSY1246 strains expressing *AbPYKS*. (**a**) Representative LC-MS/MS multiple reaction monitoring (MRM) chromatogram for detection of MPOB in the extracellular medium of CSY1246 expressing *AbPYKS* only from a low-copy plasmid (pCS4246). The three characteristic MPOB isoform peaks are labelled with (I), (II), and (III). LC-MS/MS analysis was performed after growth in selective media at 30 °C for 48 h. (**b**) Relative abundance of NMPy and MPOB (all 3 peaks) in the extracellular media of CSY1246 expressing *AbPYKS* (pCS4246), *AbCYP82M3* (pCS4247), and one of four CPRs (pCS4200-4203) from low-copy plasmids after 48 h of growth at 30 °C in selective media. Plus and minus symbols indicate presence or absence of gene. Data represent mean of n = 3 biologically independent samples (open circles); error bars indicate standard deviation. Source data of Supplementary Figure 9b are provided as a source data file.

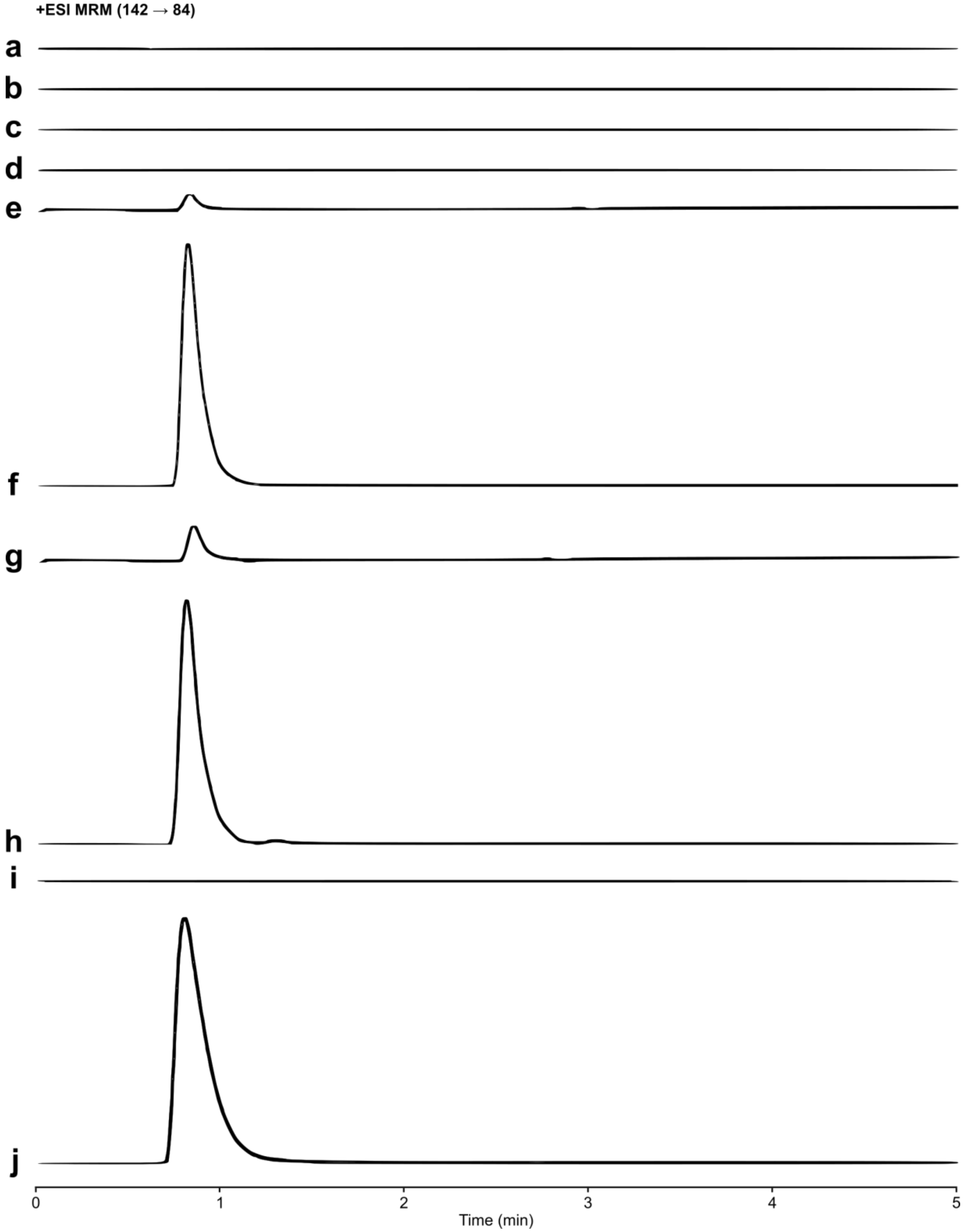

Supplementary Figure 10. Elucidation of the co-substrate for spontaneous condensation with NMPy to form hygrine. Cultures were incubated at 30 °C and 250 rpm for 48 hours prior to LC-MS/MS analysis. Traces show hygrine accumulation (+ESI MRM m/z^+ 142 → 84) and are representative of n = 3 biologically independent samples. Conditions: (**a**) CEN.PK2 only; (**b**) CEN.PK2 with 0.1% potassium acetate; (**c**) CEN.PK2 with 0.1% lithium acetoacetate; (**d**) CSY1235 expressing *AbPMT1* and *DmMPO1*$^{\Delta C\text{-}PTS1}$ from plasmids pCS4193 and pCS4238; (**e**) CSY1235 expressing *AbPMT1* and *DmMPO1*$^{\Delta C\text{-}PTS1}$ from plasmids pCS4193 and pCS4238, and with 0.1% potassium acetate; (**f**) CSY1235 expressing *AbPMT1* and *DmMPO1*$^{\Delta C\text{-}PTS1}$ from plasmids pCS4193 and pCS4238, and with 0.1% lithium acetoacetate; (**g**) CSY1243 with 0.1% potassium acetate; (**h**) CSY1243 with 0.1% lithium acetoacetate; (**i**) 100 mg/L NMPy with 0.1% potassium acetate; (**j**) 100 mg/L NMPy with 0.1% lithium acetoacetate.

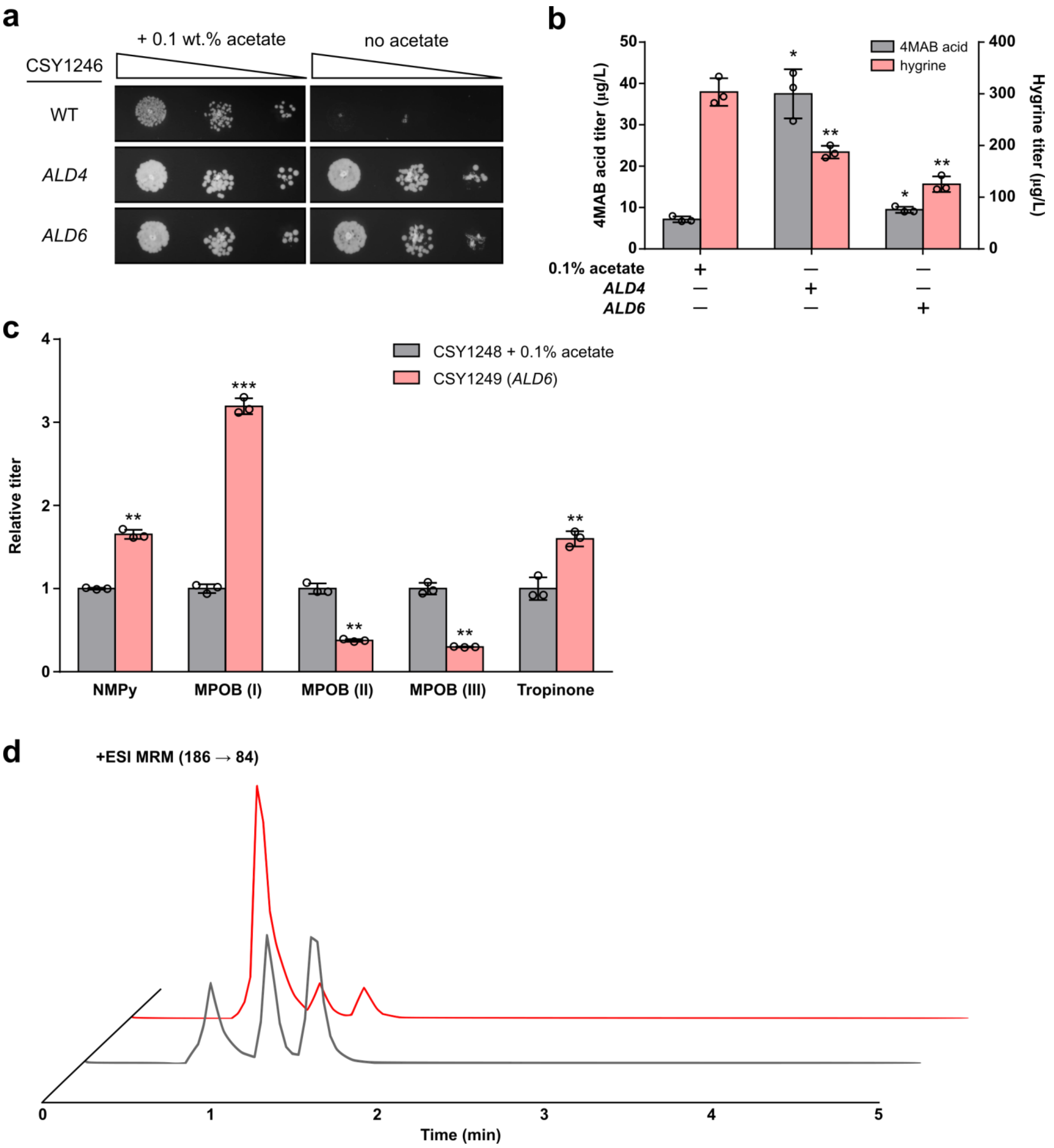
a
+ 0.1 wt.% acetate
no acetate
CSY1246
WT
ALD4
ALD6
b
4MAB acid titer (μg/L)
Hygrine titer (μg/L)
4MAB acid
hygrine
0.1% acetate
ALD4
ALD6
c
Relative titer
CSY1248 + 0.1% acetate
CSY1249 (ALD6)
NMPy
MPOB (I)
MPOB (II)
MPOB (III)
Tropinone
d
+ESI MRM (186 → 84)
Time (min)

Supplementary Figure 11. Effect of reconstituting *ALD6* activity on metabolite flux through NMPy towards tropine in CSY1249. (**a**) Effect of reconstituting functional *ALD4* or *ALD6* genes on the growth of the NMPy-producing yeast strain (CSY1246) with and without acetate supplementation. *ALD4* and *ALD6* were expressed from low-copy plasmids. 'WT' indicates CSY1246 with control (BFP) plasmid. Adjacent columns show ten-fold dilutions. (**b**) Production of 4MAB acid and hygrine side products with reconstituted acetate metabolism in engineered yeast strain CSY1246. Plus and minus symbols indicate presence or absence of fed metabolite (acetate) or *ALD4* and *ALD6* genes expressed from low-copy plasmids. Side product abundances were measured by LC-MS/MS analysis of the extracellular medium after 48 hours of growth in selective media supplemented with or without 0.1% w/v potassium acetate at 25 ^{o}C. (**c**) Production of intermediates between NMPy and tropinone in engineered strains with and without functional *ALD6*. Intermediate abundances were measured by LC-MS/MS analysis of the extracellular media of CSY1248 grown in non-selective media supplemented with 0.1% w/v potassium acetate (grey) or CSY1249 grown in non-selective media without acetate supplementation (pink) at 25 ^{o}C for 48 h. For (b) and (c), data represent mean of n = 3 biologically independent samples (open circles); error bars indicate standard deviation. Student's two-tailed t-test: *P < 0.05, **P < 0.01, ***P < 0.001. (**d**) Representative MRM chromatograms for MPOB production from CSY1248 (grey) and CSY1249 (red) cultured as described in (c). Source data of Supplementary Figure 11b and 11c are provided as a source data file.

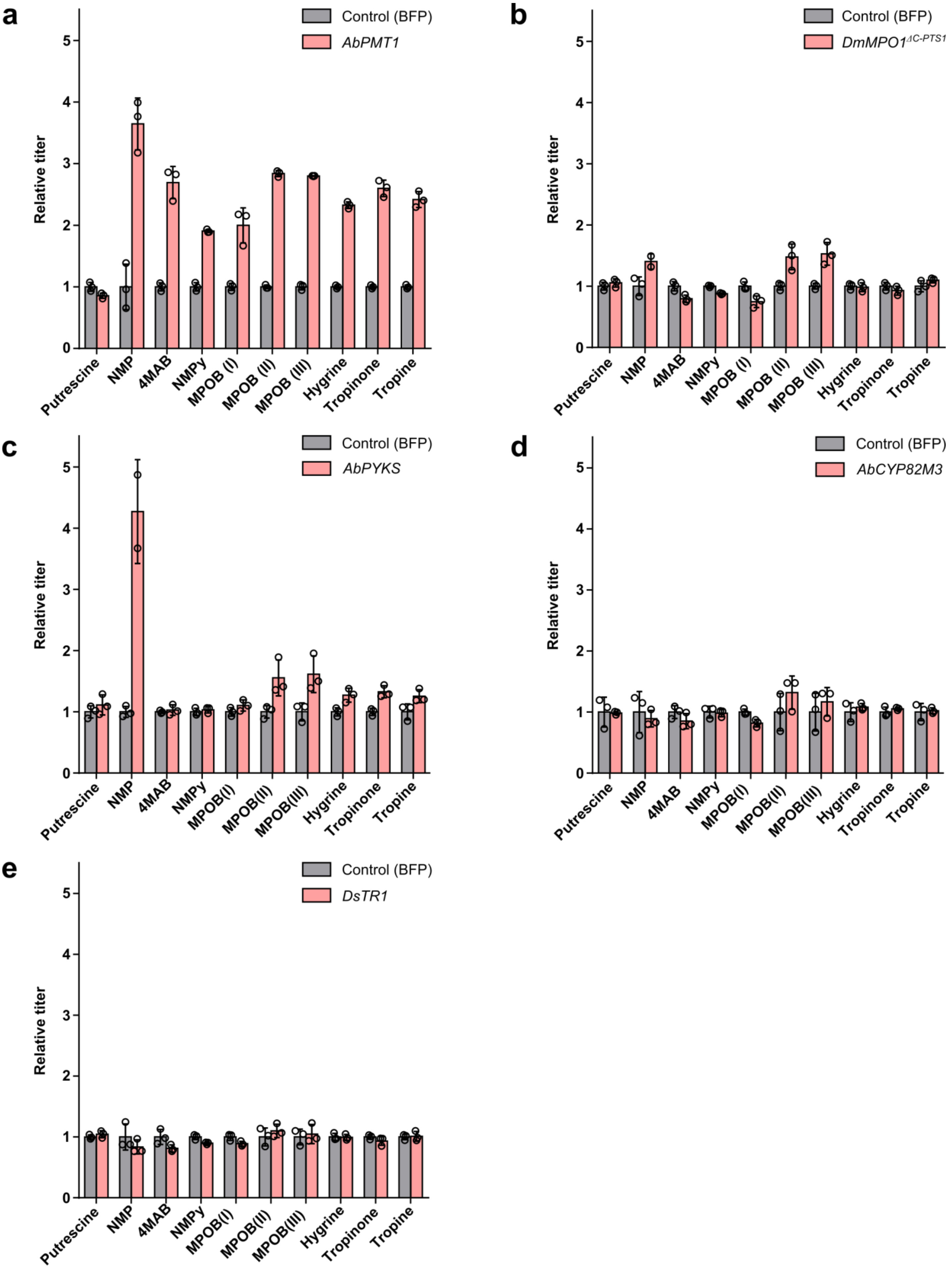
a
Control (BFP)
AbPMT1
Relative titer
0
1
2
3
4
5
Putrescine
NMP
4MAB
NMPy
MPOB (I)
MPOB (II)
MPOB (III)
Hygrine
Tropinone
Tropine
b
Control (BFP)
$DmMPO1^{\Delta C\text{-}PTS1}$
Relative titer
0
1
2
3
4
5
Putrescine
NMP
4MAB
NMPy
MPOB (I)
MPOB (II)
MPOB (III)
Hygrine
Tropinone
Tropine
c
Control (BFP)
AbPYKS
Relative titer
0
1
2
3
4
5
Putrescine
NMP
4MAB
NMPy
MPOB(I)
MPOB(II)
MPOB(III)
Hygrine
Tropinone
Tropine
d
Control (BFP)
AbCYP82M3
Relative titer
0
1
2
3
4
5
Putrescine
NMP
4MAB
NMPy
MPOB(I)
MPOB(II)
MPOB(III)
Hygrine
Tropinone
Tropine
e
Control (BFP)
DsTR1
Relative titer
0
1
2
3
4
5
Putrescine
NMP
4MAB
NMPy
MPOB(I)
MPOB(II)
MPOB(III)
Hygrine
Tropinone
Tropine

Supplementary Figure 12. Metabolic bottleneck analysis of tropine-producing strain CSY1249. An additional copy of each biosynthetic enzyme between putrescine and tropine was expressed from the following low-copy plasmids in strain CSY1249: (**a**) *AbPMT1*, pCS4193; (**b**) $DmMPO1^{\Delta C\text{-}PTS1}$, pCS4238; (**c**) *AbPYKS*, pCS4246; (**d**) *AbCYP82M3*, pCS4247; (**e**) *DsTR1*, pCS4310; or a BFP control (pCS4208, pCS4212, or pCS4213) corresponding to the same auxotrophic marker as each biosynthetic gene plasmid. Transformed strains were cultured in appropriate selective media at 25 °C for 48 hours prior to quantification of metabolites in the growth medium by LC-MS/MS. Data indicate the mean of n = 2 (panel b, $DmMPO1^{\Delta C\text{-}PTS1}$, NMP; panel c, *AbPYKS*, NMP) or n = 3 (all other samples) biologically independent samples (open circles) and error bars show standard deviation. Source data are provided as a source data file.

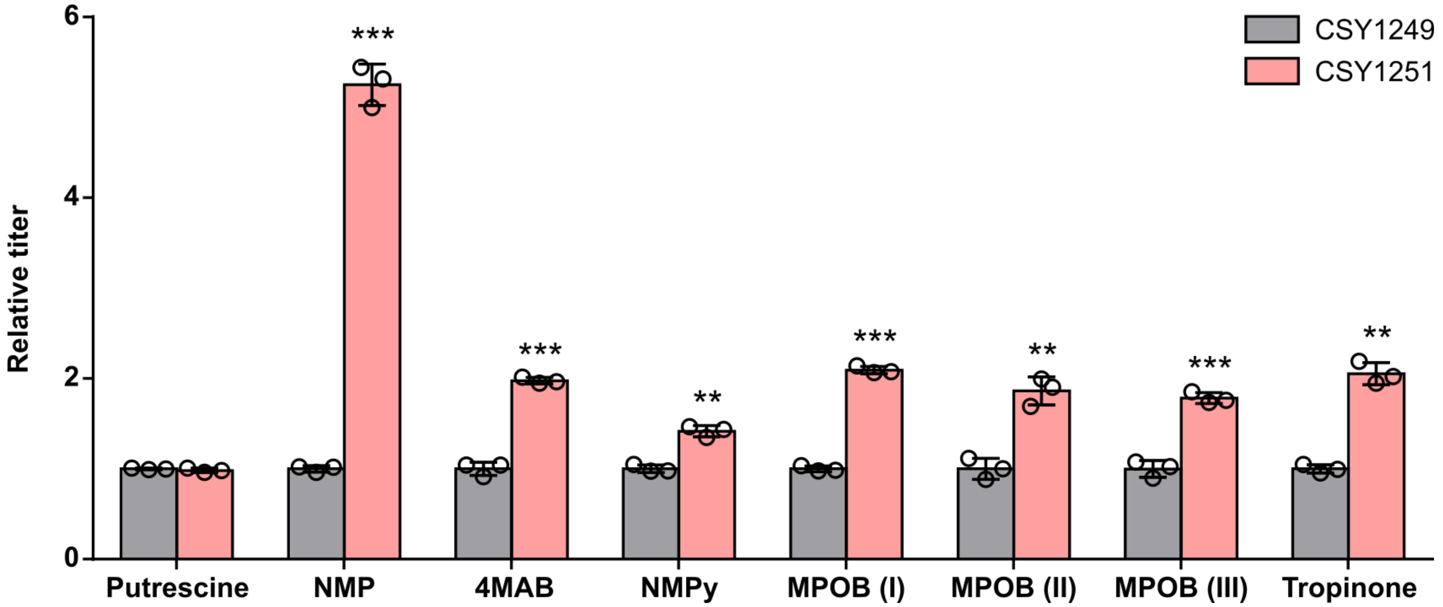

Supplementary Figure 13. Effect of extra copies of PMT and PYKS in CSY1251 on the production of intermediates between putrescine and tropine. Strains CSY1249 and CSY1251 were cultured in non-selective media at 25 ^{o}C for 48 hours and pathway intermediates in the growth media were quantified by LC-MS/MS analysis. Data indicate the mean of n = 3 biologically independent samples (open circles) and error bars indicate standard deviation. Student's two-tailed t-test: *P < 0.05, **P < 0.01, ***P < 0.001. Source data are provided as a source data file.

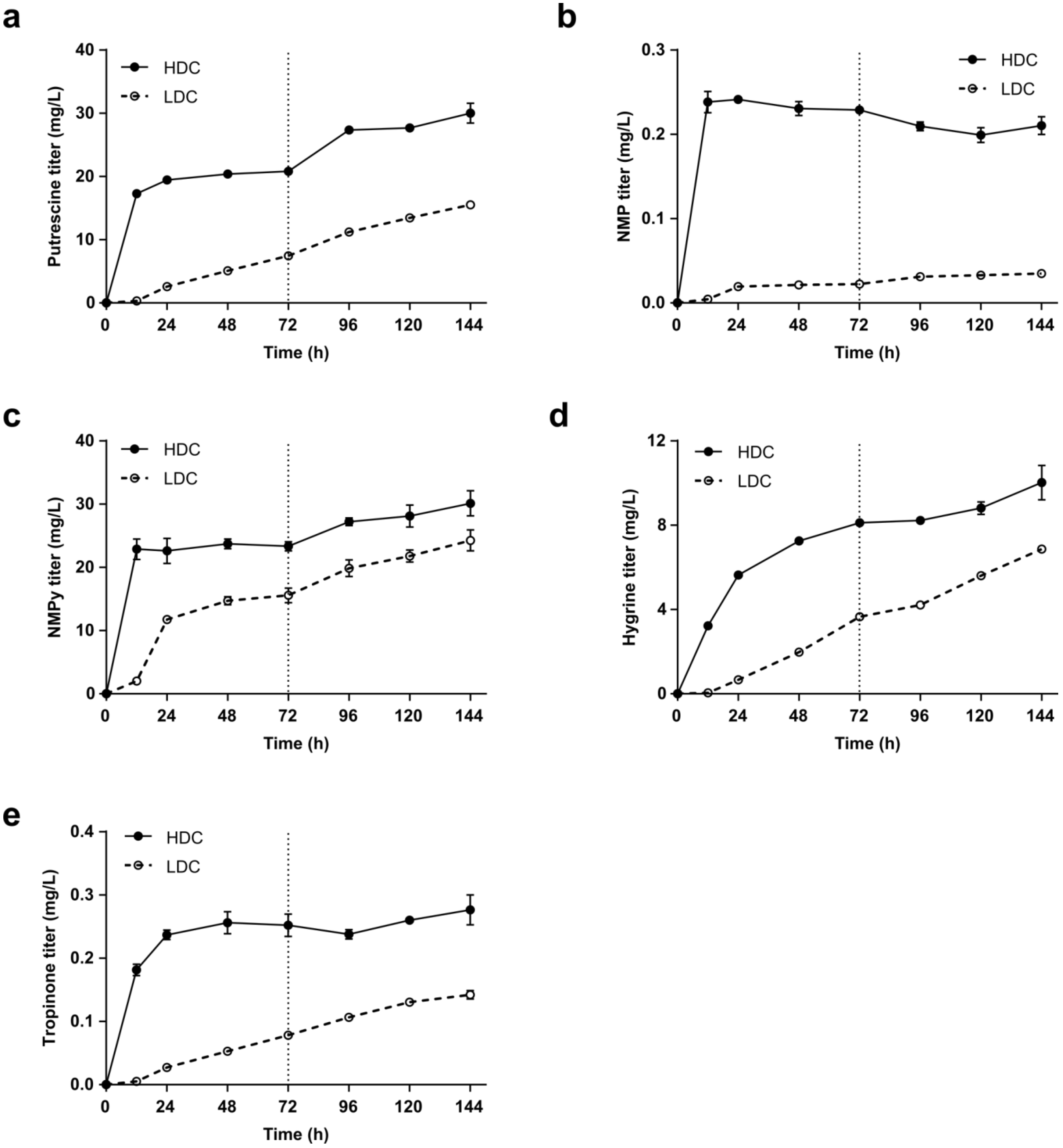

Supplementary Figure 14. Production of TA precursors in shake-flask cultures of the tropine-producing strain CSY1251 over six days of growth. Low-density (LDC) and high-density (HDC) cultures were grown in non-selective media (YNB-G; YNB + 1× amino acids + 2% dextrose + 5% glycerol) at 25 °C and 300 rpm. At t = 72 hours (dotted vertical line), cultures were supplemented with additional amino acids, dextrose, and glycerol to final concentrations of 1×, 2%, and 2%, respectively. Metabolite titers in the growth medium were quantified by LC-MS/MS analysis. Data indicate the mean of n = 3 biologically independent samples and error bars show standard deviation. Source data are provided as a source data file.

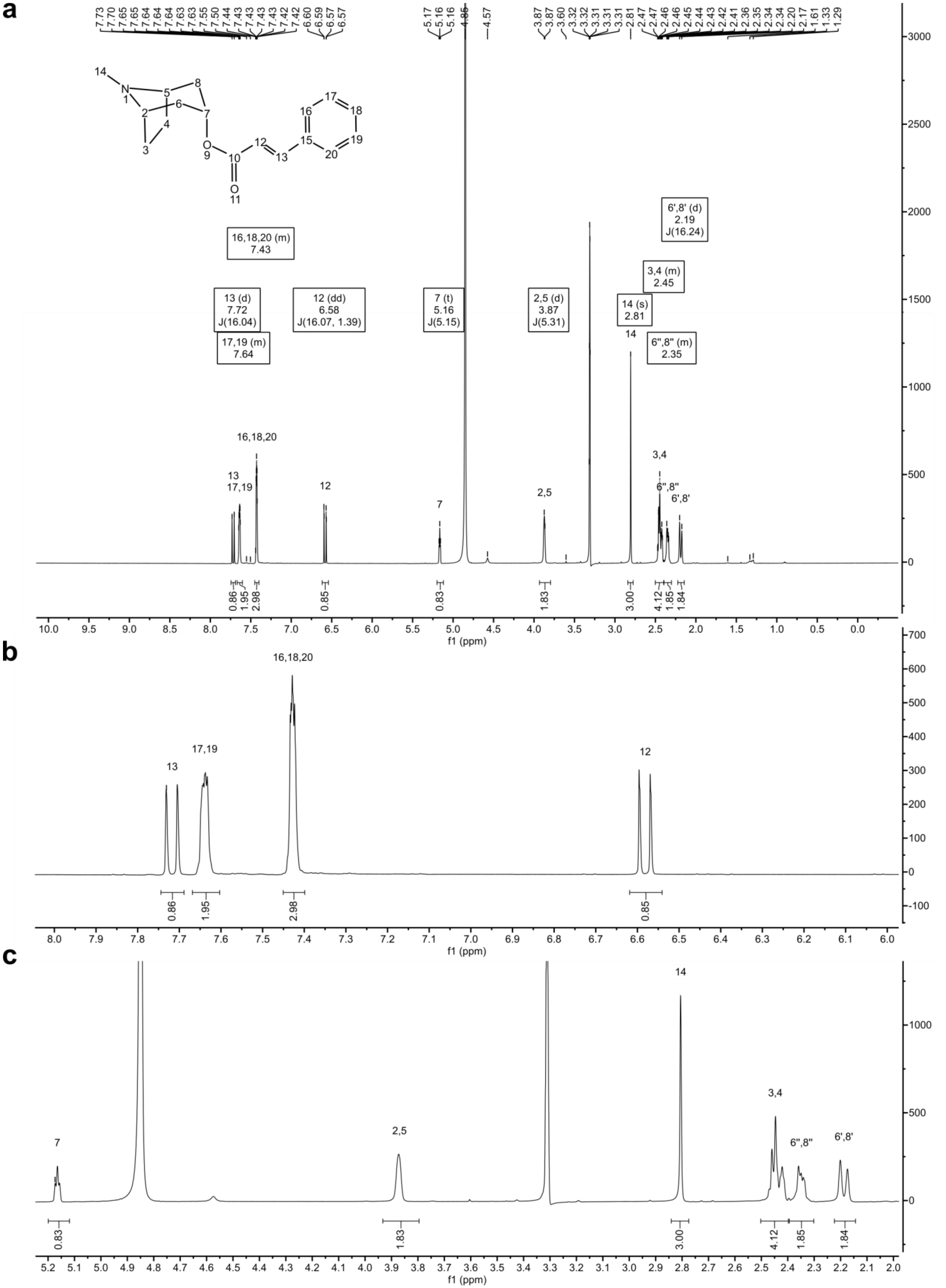
a
7.73 7.70 7.65 7.64 7.64 7.64 7.63 7.63 7.55 7.50 7.44 7.43 7.43 7.43 7.43 7.42 7.42 6.60 6.59 6.57 6.57
5.17 5.16 5.16 4.85 4.57
3.87 3.87 3.60 3.32 3.32 3.31 3.31 3.31 2.81 2.47 2.46 2.46 2.45 2.44 2.43 2.42 2.41 2.36 2.35 2.34 2.34 2.20 2.17 1.61 1.33 1.29
16,18,20 (m)
7.43
13 (d)
7.72
J(16.04)
17,19 (m)
7.64
12 (dd)
6.58
J(16.07, 1.39)
7 (t)
5.16
J(5.15)
2,5 (d)
3.87
J(5.31)
14 (s)
2.81
3,4 (m)
2.45
6',8' (d)
2.19
J(16.24)
6'',8'' (m)
2.35
16,18,20
13
17,19
12
7
2,5
14
3,4
6'',8''
6',8'
0.86 1.95 2.98 0.85 0.83 1.83 3.00 4.12 1.85 1.84
f1 (ppm)
b
16,18,20
17,19
13
12
0.86 1.95 2.98 0.85
f1 (ppm)
c
7
2,5
14
3,4
6'',8''
6',8'
0.83 1.83 3.00 4.12 1.85 1.84
f1 (ppm)

Supplementary Figure 15. ^{1}H-NMR spectrum of cinnamoyl-3α-tropine standard. (**a**) Full spectrum from δ = -0.5 to 10.0. Peak assignments are indicated for corresponding numbered atoms bearing one or more hydrogens. (**b**) Zoomed spectrum from panel (a) showing δ = 6.0 to 8.0. (**c**) Zoomed spectrum from panel (a) showing δ = 2.0 to 5.2. For (b) and (c), peak assignments correspond to atom numbering in panel (a).

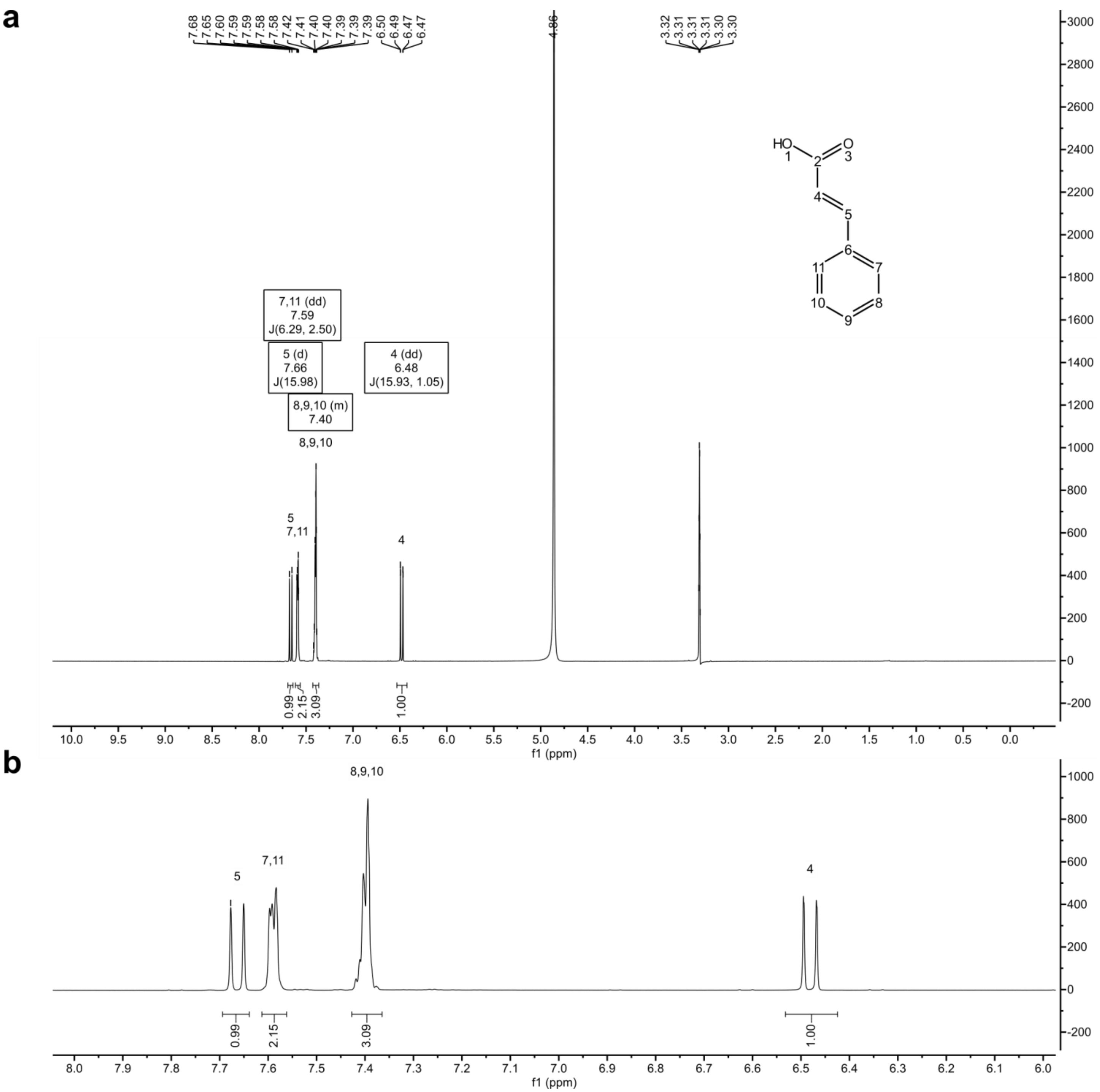

Supplementary Figure 16. ^{1}H-NMR spectrum of *trans*-cinnamic acid standard. (**a**) Full spectrum from δ = -0.5 to 10.0. Peak assignments are indicated for corresponding numbered atoms bearing one or more hydrogens. (**b**) Zoomed spectrum from panel (a) showing δ = 6.0 to 8.0. Peak assignments correspond to atom numbering in panel (a).

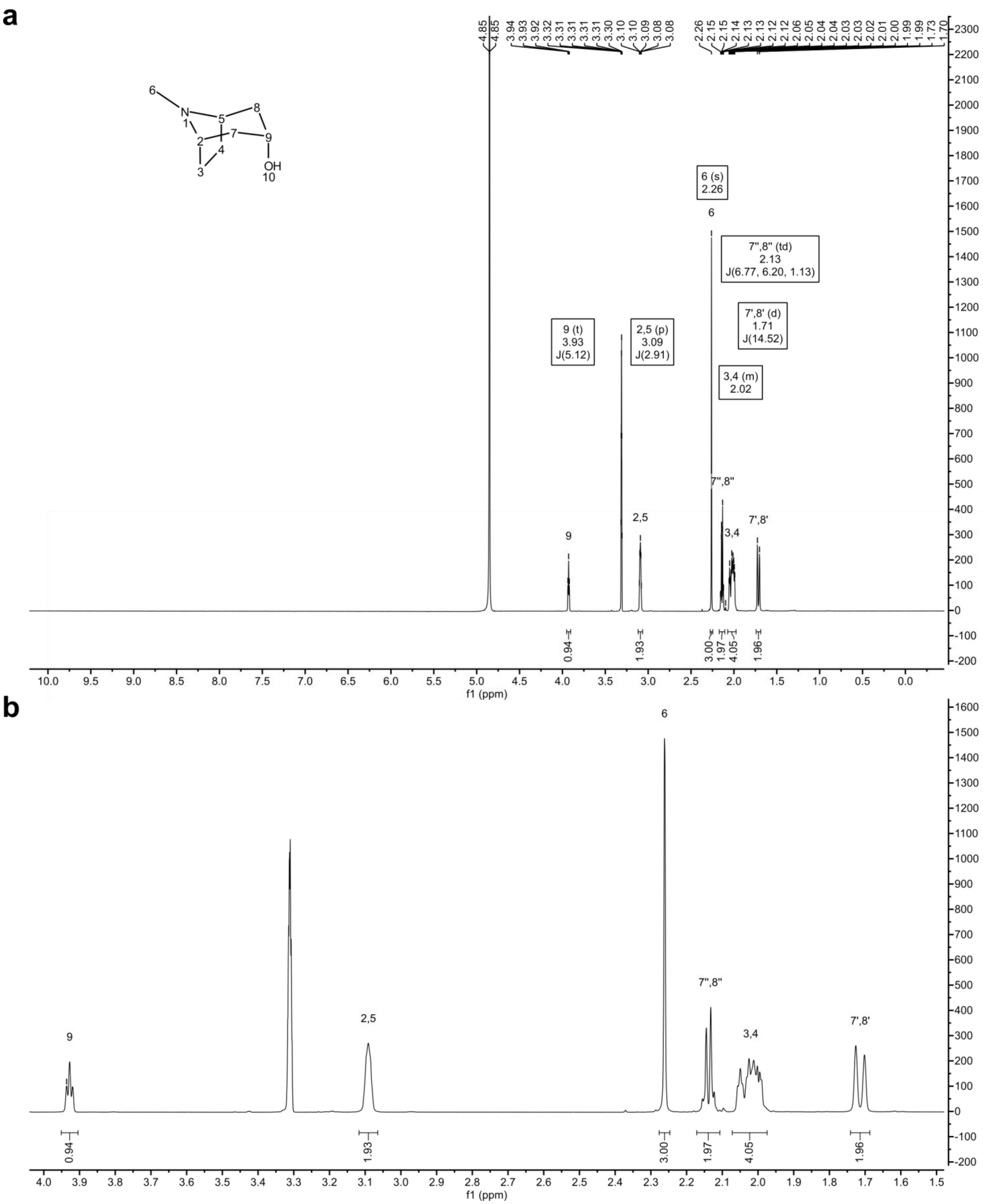

Supplementary Figure 17. ^{1}H-NMR spectrum of tropine standard. α-tropine was used in this case. (**a**) Full spectrum from δ = -0.5 to 10.0. Peak assignments are indicated for corresponding numbered atoms bearing one or more hydrogens. (**b**) Zoomed spectrum from panel (a) showing δ = 1.5 to 4.0. Peak assignments correspond to atom numbering in panel (a).

a

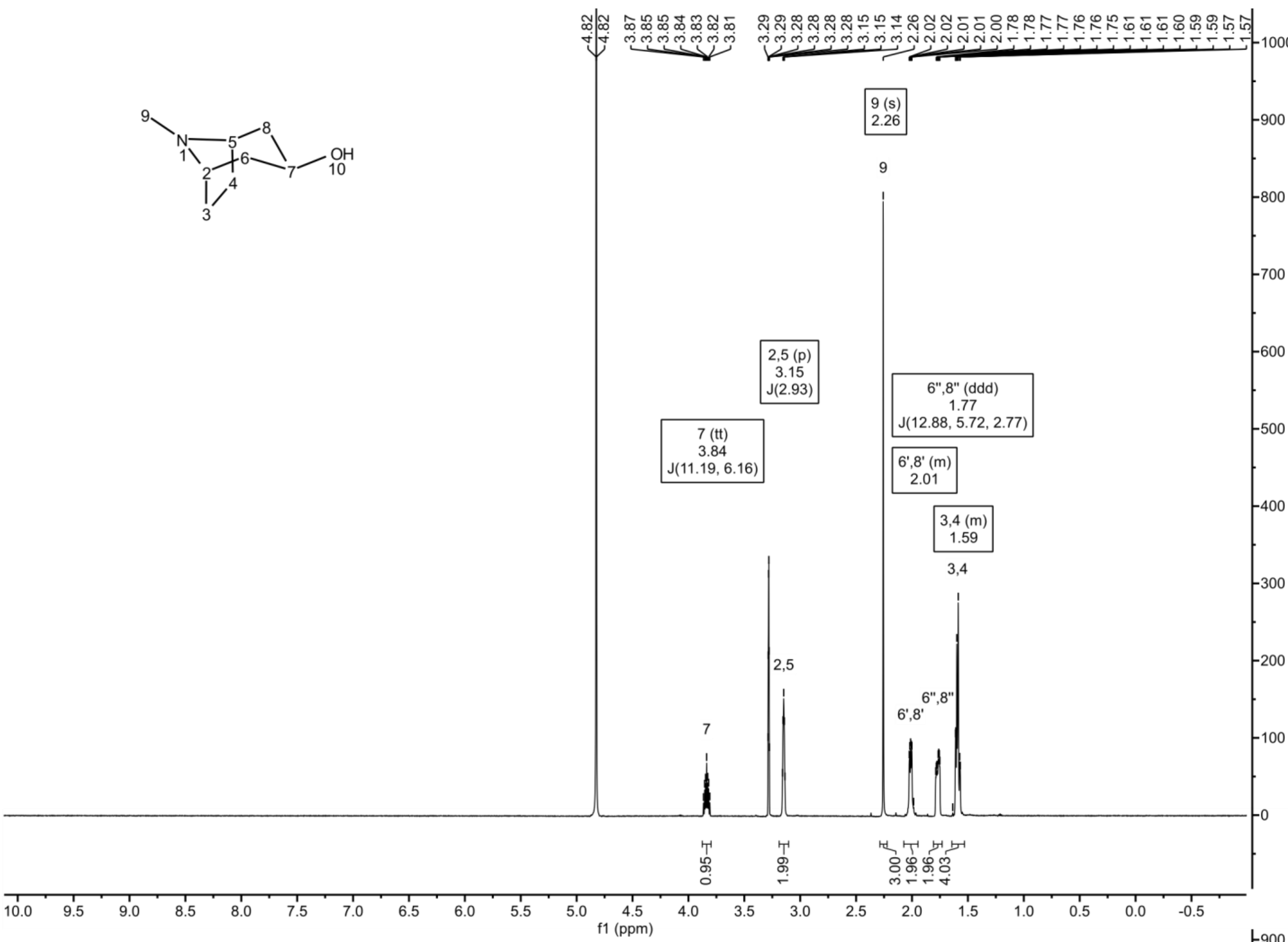

9 (s)
2.26
2,5 (p)
3.15
J(2.93)
7 (tt)
3.84
J(11.19, 6.16)
6",8" (ddd)
1.77
J(12.88, 5.72, 2.77)
6',8' (m)
2.01
3,4 (m)
1.59
OH
10
f1 (ppm)

b

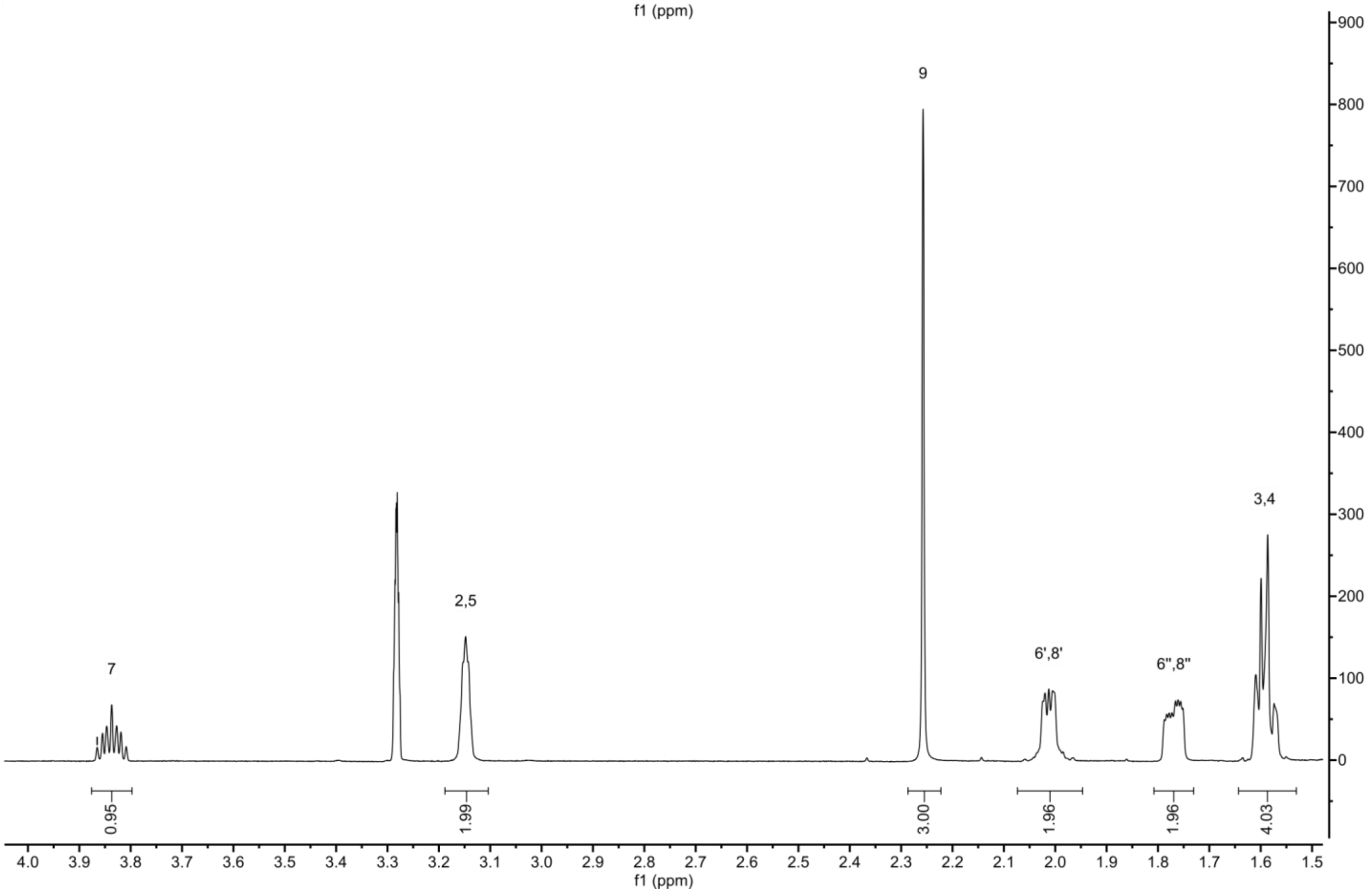

9
3,4
2,5
7
6',8'
6",8"
0.95
1.99
3.00
1.96
1.96
4.03
f1 (ppm)

Supplementary Figure 18. ^{1}H-NMR spectrum of pseudotropine standard. β-tropine was used in this case. (**a**) Full spectrum from δ = -0.5 to 10.0. Peak assignments are indicated for corresponding numbered atoms bearing one or more hydrogens. (**b**) Zoomed spectrum from panel (a) showing δ = 1.5 to 4.0. Peak assignments correspond to atom numbering in panel (a).

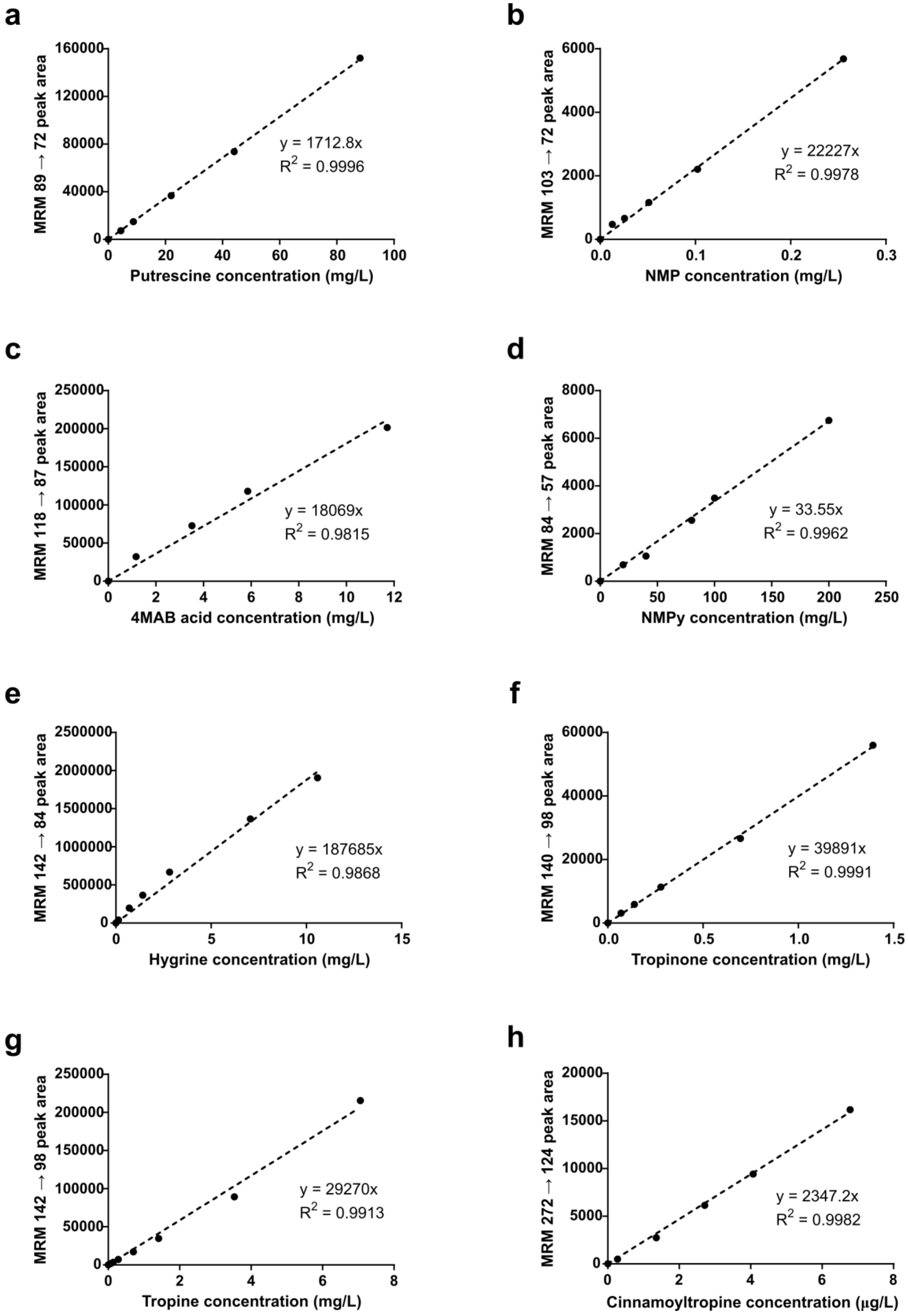
a
MRM 89 → 72 peak area
Putrescine concentration (mg/L)
y = 1712.8x
R^2 = 0.9996
b
MRM 103 → 72 peak area
NMP concentration (mg/L)
y = 22227x
R^2 = 0.9978
c
MRM 118 → 87 peak area
4MAB acid concentration (mg/L)
y = 18069x
R^2 = 0.9815
d
MRM 84 → 57 peak area
NMPy concentration (mg/L)
y = 33.55x
R^2 = 0.9962
e
MRM 142 → 84 peak area
Hygrine concentration (mg/L)
y = 187685x
R^2 = 0.9868
f
MRM 140 → 98 peak area
Tropinone concentration (mg/L)
y = 39891x
R^2 = 0.9991
g
MRM 142 → 98 peak area
Tropine concentration (mg/L)
y = 29270x
R^2 = 0.9913
h
MRM 272 → 124 peak area
Cinnamoyltropine concentration (μg/L)
y = 2347.2x
R^2 = 0.9982

Supplementary Figure 19. Representative standard curves for metabolite titer measurements in culture media. All standard curves were prepared in either non-selective (YNB(A)-SC) or selective (YNB(A)-DO) media, depending on the corresponding culture conditions. Dotted line indicates linear regression fit; regression equation and R^2 are indicated. Source data are provided as a source data file.

Supplementary Note 1. Optimization of MPO activity via sub-cellular localization and truncation studies.

Prior studies have shown that while NtPMT is expressed in the cytosol of tobacco cells, NtMPO1 localizes to the peroxisome lumen[5]. We performed *in silico* prediction of enzyme subcellular localization using the SherLoc2 utility for signal peptide detection[6], which revealed that NtMPO1 harbors a strong yeast consensus peroxisome-targeting sequence (PTS) at its C-terminus (Ala-Lys-Leu, denoted PTS1). This observation suggested that NtMPO1 may localize to peroxisomes when expressed heterologously in yeast (Supplementary Figure 5a). We confirmed this prediction via fluorescence microscopy of wild-type yeast cells expressing either N- or C-terminal GFP-tagged *AbPMT1* (pCS4209, 4210) and *NtMPO1* (pCS4214, 4215) from low-copy plasmids, which indicated that while AbPMT1 is found primarily in the cytosol, localization of NtMPO1 to peroxisomes is contingent on an exposed C-terminal PTS (Supplementary Figure 5b, Fig. 3b). Given that yeast peroxisomes likely do not possess the same transport machinery for exchanging NMP, 4MAB, and NMPy as plant peroxisomes, we hypothesized that the poor apparent activity of NtMPO1 was caused by its sequestration within peroxisomes. However, cytosolic expression of NtMPO1 achieved by masking the C-terminal PTS with a GFP fusion did not significantly impact extracellular 4MAB or NMPy levels (Supplementary Figure 5c), indicating that its peroxisomal localization was likely not the primary factor limiting 4MAB and NMPy production.

We next examined whether orthologs of NtMPO1 from TA-producing species exhibit greater activity in yeast. We performed a tBLASTn search of the transcriptomes of *A. belladonna* and *Datura metel* in the 1000 Plants Project database[7] using the amino acid sequence of NtMPO1 as a query and an E-value threshold of 10^{-150}. We identified two full-length ortholog sequences, denoted *AbMPO1* and *DmMPO1,* which each shared 91% sequence identity with *NtMPO1* (Supplementary Figure 6a). We obtained yeast codon-optimized sequences for the two orthologs and cloned them into low-copy expression plasmids. To evaluate their activity, we co-expressed each of the three MPO variants (pCS4218, 4227, 4228) with *AbPMT1* (pCS4193) from low-copy plasmids in our putrescine-overproducing strain (CSY1235), and measured 4MAB and NMPy accumulation in the medium by LC-MS/MS following 48 hours of growth. DmMPO1 showed comparable levels of 4MAB and NMPy production to the original NtMPO1 variant, whereas AbMPO1 exhibited significantly lower activity, despite differing by less than 10% in amino acid sequence (Supplementary Figure 6b).

We next examined whether the differences in activity between the three orthologs could be partially attributed to structural differences in their active sites. We constructed template-based homology models of NtMPO1, AbMPO1, and DmMPO1 based on the crystal structure of a *Pisum sativum* copper-containing amino oxidase (PDB: 1KSI) using the RaptorX web server[8]. Although no clear conclusions could be drawn regarding relationships between differences in active

site or substrate-binding pocket and observed *in vivo* activity, the homology models indicated that the orthologs possess long, unstructured N- and C-terminal tail regions (Supplementary Figure 6c). Although such unstructured regions are frequently caused by failure of the homology modeling software to adequately model regions of the polypeptide chain with poor homology to an existing template, we verified whether these unstructured regions were modeling artifacts or genuine structural features by testing truncations of the two active orthologs, NtMPO1 and DmMPO1. N-terminal truncations removed the first 84 and 81 residues of the two orthologs, respectively; whereas C-terminal truncations removed the last 21 residues. Since the importance of the PTS for MPO activity was unclear, we also constructed C-terminal truncations wherein the unstructured tail was removed but the PTS was retained (denoted $^{\Delta C\text{-}PTS1}$). We co-expressed each of the MPO truncations (pCS4233-4238) with *AbPMT1* (pCS4193) from low-copy plasmids in the putrescine-overproducing strain CSY1235, and quantified 4MAB and NMPy accumulation in the media after 48 hours of growth by LC-MS/MS. No significant differences in either 4MAB or NMPy titers were observed between the NtMPO1 truncations (Supplementary Figure 6d, Fig. 3c). However, removal of the C-terminal unstructured region from DmMPO1 while retaining the C-terminal PTS tripeptide resulted in a 19% increase in extracellular 4MAB levels relative to the wild-type DmMPO1 enzyme and a 55% increase relative to wild-type NtMPO1. Although only an 11% increase in NMPy titer was observed between these variants, we suspected that the additional 4MAB accumulation would improve flux towards downstream intermediates upon reconstitution of subsequent biosynthetic steps in the pathway. As such, all further optimization of the MPO step was performed using this truncated variant, denoted *DmMPO1*$^{\Delta C\text{-}PTS1}$.

Supplementary Note 2. Elucidation of co-substrate for spontaneous condensation with NMPy to form hygrine.

To elucidate the co-substrate that condenses with NMPy to produce hygrine, we performed experiments in which we assayed combinations of NMPy-producing and -non-producing yeast strains in media supplemented with acetate or acetoacetate for hygrine accumulation by LC-MS/MS (Supplementary Figure 10). No hygrine accumulated in cultures of wild-type yeast (CEN.PK2) fed either acetate or acetoacetate following 48 hours of growth. In cultures of NMPy-producing strains, hygrine accumulated with acetate or acetoacetate supplementation (CSY1235 expressing *AbPMT1* and *DmMPO1*$^{\Delta C\text{-}PTS1}$: 38 μg/L hygrine with acetate, 1.9 mg/L with acetoacetate; CSY1243: 108 μg/L with acetate, 3.5 mg/L with acetoacetate), but not in the absence of either. Hygrine accumulation was detected (to 17 mg/L) when 100 mg/L NMPy was incubated in media with acetoacetate in the absence of cells, but not when NMPy was incubated with acetate alone. These data suggest that acetate supplementation may increase levels of an endogenous keto-metabolite, such as acetoacetate or acetoacetyl-CoA, which condenses with NMPy to form hygrine.

Supplementary Note 3. Elimination of acetate auxotrophy to decrease spontaneous hygrine production.

We attempted to reduce hygrine production resulting from spontaneous condensation with an endogenous keto-metabolite derived from acetate. We examined the impact of removing fed acetate in the NMPy-producing strain (CSY1246), which lacks the enzymes for MPOB and tropinone biosynthesis, to isolate the acetate-dependent hygrine production mechanism from the MPOB-dependent one (Fig. 4a). We rescued acetate auxotrophy in CSY1246 by expressing *ALD4* and *ALD6* on low-copy plasmids (pCS4248, 4249), and evaluated accumulation of hygrine and 4MAB acid via LC-MS/MS analysis after 48 hours. While reconstitution of *ALD4* or *ALD6* enabled CSY1246 to grow in the absence of fed acetate (Supplementary Figure 11a), addition of *ALD4* caused a five-fold increase in the accumulation of 4MAB acid while *ALD6* did not produce a significant increase (Supplementary Figure 11b). Elimination of acetate feeding with *ALD4* or *ALD6* resulted in 38% and 59% decreases in hygrine accumulation, respectively, confirming that condensation with an acetate-derived metabolite contributes substantially to hygrine production. As acetate is an essential metabolite, it may not be feasible to completely eliminate this route for hygrine production. We re-integrated *ALD6* into our tropine-producing strain (CSY1248) to make strain CSY1249, and measured the accumulation of metabolites between NMPy and tropine via LC-MS/MS analysis after 48 hours of growth. Restoration of acetate metabolism in CSY1249 resulted in a 2.7-fold increase in tropine titers (1.5 mg/L) relative to CSY1248 (565 µg/L) and a 1.6-fold increase in hygrine accumulation (Fig. 4d). *ALD6* expression resulted in increases in NMPy and tropinone production and MPOB consumption (Supplementary Figure 11c), suggesting that elimination of acetate auxotrophy may improve metabolite flux through the entire pathway despite increasing the hygrine side reaction.

Supplementary Note 4. Optimization of media composition for *de novo* tropine biosynthesis in CSY1251.

We examined the impact of media composition on tropine production. We cultured CSY1251 in defined (yeast nitrogen base; YNB) or complex (yeast extract and peptone; YP) media supplemented with 0-5× amino acids, 2-4% dextrose as a carbon source for growth, and 2-5% of other carbon sources, and measured tropine titers in the media by LC-MS/MS after 72 hours (Fig. 4e). Increasing amino acid concentration to 5× or 2.5× resulted in 25% and 18% decreases in tropine titer in defined and complex media, respectively. Doubling the dextrose concentration from 2% to 4% decreased tropine titers by 67% in defined media, but increased titers by 55% in complex media. Supplementation of defined media with 2% dextrose and 2% galactose, raffinose, trehalose, arabinose, or sorbitol resulted in 13-60% increases in tropine titer relative to 4% dextrose, whereas all carbon sources except glycerol resulted in 48-68% decreases in tropine titer relative to 2% dextrose only. Supplementation of dextrose and glycerol increased tropine production relative to dextrose alone; under the best media condition (YNB + 1X amino acids + 2% dextrose + 5% glycerol; denoted YNB-G), tropine titers reached 4.9 mg/L, 35% greater than with 2% dextrose alone.

We observed that synthetic defined media outperformed richer YP media, contrary to previous reports of heterologous biosynthesis in yeast[9,10]. Similarly, higher starting concentrations of amino acids, dextrose, and other fermentable sugars resulted in decreased tropine production, potentially due to higher protein synthesis and growth rates leading to increased enzyme misfolding[10], as well as repression of late-stage promoters and endogenous pathways for biosynthesis and transport of basic amino acids[11,12]. Among alternate carbon sources, only glycerol supplementation increased tropine production, possibly due to its stabilization of cellular lipid membranes, improved folding and stability of heterologous proteins, and its role in the regeneration of the NADPH cofactor required for the activity of AbCYP82M3 and DsTR1[10].

Supplementary Note 5. Substrate feeding experiments to elucidate the stereochemistry of the EcCS reaction.

Given that a prior report of recombinant EcCS detected acyltransferase activity only on β-tropane isomers, such as pseudotropine and methylecgonine, and not on α-tropanes[13], we performed feeding experiments to support the observed EcCS activity on α-tropine. We cultured wild-type yeast (CEN.PK2) or the tropine-producing strain (CSY1251) transformed with plasmid-based *AtPAL1* (pCS4252), *At4CL5* and *EcCS* (pCS4207) in media supplemented with pure α-tropine and/or *trans*-cinnamic acid for 72 hours and analyzed the medium for cinnamoyltropine accumulation by LC-MS/MS (Fig. 5b-i). In the transformed CEN.PK2 strain, cinnamoyltropine was detected with expression of *AtPAL1*, *At4CL5*, and *EcCS* and supplementation of 0.5 mM α-tropine, but not in the absence of any of these components, validating that EcCS expressed in yeast exhibits activity on the α isomer of tropine. In the transformed tropine-producing strain (CSY1251), cinnamoyltropine was detected with expression of *At4CL5* and *EcCS* and addition of 0.2 mM cinnamic acid to the media, as well as with expression of *AtPAL1* in place of cinnamate supplementation, but not in the absence of a cinnamate source, verifying that the detected tropane ester is derived from *trans*-cinnamic acid.

Supplementary Discussion

Our analysis of intermediate accumulation between NMPy and tropine initially identified *AbCYP82M3* as a pathway bottleneck, consistent with a prior study indicating that this enzyme is rate-limiting for tropinone biosynthesis in *A. belladonna*[2]. We improved stability of P450 expression from genomic integration compared to plasmid-based expression, consistent with previous reports of ER stress caused by plasmid-based overexpression of P450 enzymes in yeast[14]. However, the impact of this alteration on MPOB accumulation remains unclear. We observed three distinct peaks in the MRM (186 → 84) spectrum for MPOB produced in yeast (Supplementary Figure 9a, S11d), in contrast to a single peak previously observed for chemically synthesized MPOB[2], suggesting that additional isomers of MPOB may be formed by NMPy condensation in yeast. The three MPOB peaks increased upon expression of *AbPYKS* in CSY1246 (Supplementary Figure 9b), suggesting that all isomers are generated by or derived from the product of AbPYKS. The inconsistent patterns of peak reduction upon addition of *AbCYP82M3* (Supplementary Figure 9b) and reconstitution of *ALD6* (Supplementary Figure 11c, d) suggest that the P450 enzyme may exhibit different affinities for each isomer. Substrate feeding experiments may elucidate the identity of the three MPOB peaks and the kinetic preferences of AbCYP82M3 for each, which could inform structure-guided AbPYKS engineering to improve flux through the desired MPOB substrate.

Supplementary References

1. Eudes, A. *et al.* Exploiting members of the BAHD acyltransferase family to synthesize multiple hydroxycinnamate and benzoate conjugates in yeast. *Microb. Cell Fact.* **15,** 198 (2016).
2. Bedewitz, M. A., Jones, A. D., D'Auria, J. C. & Barry, C. S. Tropinone synthesis via an atypical polyketide synthase and P450-mediated cyclization. *Nat. Commun.* **9,** 5281 (2018).
3. Bedewitz, M. A. *et al.* A root-expressed L-phenylalanine:4-hydroxyphenylpyruvate aminotransferase is required for tropane alkaloid biosynthesis in *Atropa belladonna*. **26,** 3745–3762 (2014).
4. Rubio, N. C., Bermejo-Barrera, P., Bermejo, A. M. & Moreda-Piñeiro, A. Development of a reliable method for assessing coca alkaloids in oral fluid by HPLC–MS-MS. *J. Anal. Toxicol.* (2018). doi:10.1093/jat/bky076
5. Naconsie, M., Kato, K., Shoji, T. & Hashimoto, T. Molecular evolution of n-methylputrescine oxidase in tobacco. *Plant Cell Physiol.* **55,** 436–444 (2014).
6. Briesemeister, S. *et al.* SherLoc2: A high-accuracy hybrid method for predicting subcellular localization of proteins. *J. Proteome Res.* **8,** 5363–5366 (2009).
7. Matasci, N. *et al.* Data access for the 1,000 Plants (1KP) project. *Gigascience* **3,** 17 (2014).
8. Källberg, M. *et al.* Template-based protein structure modeling using the RaptorX web server. *Nat. Protoc.* **7,** 1511–22 (2012).
9. Hahn-Hägerdal, B. *et al.* Role of cultivation media in the development of yeast strains for large scale industrial use. *Microb. Cell Fact.* **4,** 1–16 (2005).
10. Li, Y. *et al.* Complete biosynthesis of noscapine and halogenated alkaloids in yeast. *Proc. Natl. Acad. Sci.* **115,** 201721469 (2018).
11. Kayikci, O. & Nielsen, J. Glucose repression in the yeast *Saccharomyces cerevisiae*. *FEMS Yeast Res.* **15,** 1–8 (2015).
12. Peter, G. J., Důring, L. & Ahmed, A. Carbon catabolite repression regulates amino acid permeases in Saccharomyces cerevisiae via the TOR signaling pathway. *J. Biol. Chem.* **281,** 5546–5552 (2006).
13. Schmidt, G. W. *et al.* The last step in cocaine biosynthesis is catalyzed by a BAHD acyltransferase. *Plant Physiol.* **167,** 89–101 (2015).
14. Trenchard, I. J. & Smolke, C. D. Engineering strategies for the fermentative production of plant alkaloids in yeast. *Metab. Eng.* **30,** 96–104 (2015).
