# Supplementary material for: Engineering a microbial biosynthesis platform for de novo production of tropane alkaloids
Source: Nat Commun. 2019 Aug 12;10:3634. doi: 10.1038/s41467-019-11588-w (PMC6690885; doi:10.1038/s41467-019-11588-w)
Supplement: Supplementary file 3 — Reporting Summary [file 41467_2019_11588_MOESM3_ESM.pdf]

nature research

Corresponding author(s): Christina D. Smolke

Last updated by author(s): Jul 16, 2019

Reporting Summary

Nature Research wishes to improve the reproducibility of the work that we publish. This form provides structure for consistency and transparency in reporting. For further information on Nature Research policies, see Authors & Referees and the Editorial Policy Checklist .

Statistics

For all statistical analyses, confirm that the following items are present in the figure legend, table legend, main text, or Methods section.

n/a	Confirmed	
[ ]	[x]	The exact sample size (*n*) for each experimental group/condition, given as a discrete number and unit of measurement
[ ]	[x]	A statement on whether measurements were taken from distinct samples or whether the same sample was measured repeatedly
[ ]	[x]	The statistical test(s) used AND whether they are one- or two-sided<br>*Only common tests should be described solely by name; describe more complex techniques in the Methods section.*
[x]	[ ]	A description of all covariates tested
[x]	[ ]	A description of any assumptions or corrections, such as tests of normality and adjustment for multiple comparisons
[ ]	[x]	A full description of the statistical parameters including central tendency (e.g. means) or other basic estimates (e.g. regression coefficient) AND variation (e.g. standard deviation) or associated estimates of uncertainty (e.g. confidence intervals)
[x]	[ ]	For null hypothesis testing, the test statistic (e.g. *F*, *t*, *r*) with confidence intervals, effect sizes, degrees of freedom and *P* value noted<br>*Give P values as exact values whenever suitable.*
[x]	[ ]	For Bayesian analysis, information on the choice of priors and Markov chain Monte Carlo settings
[x]	[ ]	For hierarchical and complex designs, identification of the appropriate level for tests and full reporting of outcomes
[x]	[ ]	Estimates of effect sizes (e.g. Cohen's *d*, Pearson's *r*), indicating how they were calculated

Our web collection on statistics for biologists contains articles on many of the points above.

Software and code

Policy information about availability of computer code

Data collection	Agilent MassHunter Workstation LC/MS Data Acquisition for 6400 Series Triple Quadrupole software (ver. B.08.02) was used for collection of LC-MS/MS data. Agilent MassHunter Workstation Optimizer for 6400 Series Triple Quadrupole software (ver. B.08.02) was used for development of multiple reaction monitoring parameters for chemical standards. Micro-Manager software (ver. 1.4.22) was used for acquisition of microscopy data. NCBI BLAST online software (ver. 2.8.1) was used for alignment of DNA and amino acid sequences. SherLoc2 software (ver. 26-10-2009) was used for prediction of protein subcellular localization. RaptorX online software (ver. 2018) was used for construction of template-based homology models of protein structures.
Data analysis	Agilent MassHunter Workstation Qualitative Analysis Navigator software (ver. B.08.00) was used for analysis of all LC-MS/MS data. Microsoft Excel 2013 and Graphpad Prism 7 were used for analysis and statistical evaluation of all quantitative data, and for preparation of figures. NIH ImageJ software (ver. 1.52) was used for analysis of microscopy data. PyMOL software (ver. 2.2.3) was used for visualization and analysis of enzyme structures. Mestrelab Mnova with NMR plugin (ver. 12.0.4) was used for analysis of NMR data. Inkscape (ver. 0.91) was used for preparation of figure panels.

For manuscripts utilizing custom algorithms or software that are central to the research but not yet described in published literature, software must be made available to editors/reviewers. We strongly encourage code deposition in a community repository (e.g. GitHub). See the Nature Research guidelines for submitting code & software for further information.

Data

Policy information about availability of data

All manuscripts must include a data availability statement. This statement should provide the following information, where applicable:

- Accession codes, unique identifiers, or web links for publicly available datasets
- A list of figures that have associated raw data
- A description of any restrictions on data availability

Data supporting the findings of this work are available within the paper and its Supplementary Information files. A reporting summary for this Article is available as a Supplementary Information file. The datasets generated and analyzed during the current study are available from the corresponding author upon request. The

source data underlying Figures 2b-c, 2e-f, 3c-d, and 4b-f, as well as Supplementary Figures 2, 4, 5c, 6b, 6d, 7, 8a-e, 9b, 11b-c, 12a-e, 13, 14a-e, and 19a-h are provided as a Source Data file.

Field-specific reporting

Please select the one below that is the best fit for your research. If you are not sure, read the appropriate sections before making your selection.

☒ Life sciences ☐ Behavioural & social sciences ☐ Ecological, evolutionary & environmental sciences

For a reference copy of the document with all sections, see nature.com/documents/nr-reporting-summary-flat.pdf

Life sciences study design

All studies must disclose on these points even when the disclosure is negative.

Sample size	No prior estimation of requisite sample size was performed. All presented data represent measurements from sample sizes of at least three biological replicates, where biological replicates (as defined in 'Replication' below) represent independently grown microbial cultures. Since (i) engineering was performed at the cellular level, (ii) metabolite titers are a bulk measure of a cellular population, and (iii) each assayed microbial culture represents a large population of individual cells, three biological replicates were sufficient for reliable measurement of changes in metabolite production at the population level.
Data exclusions	No data were excluded from the analyses.
Replication	All measurements of metabolite titers and fluorescence microscopy were repeated on independent biological samples (i.e., independent microbial cultures or strains). All attempts at replication of results presented in this study were successful. All replicates performed in this study were biological replicates, rather than technical replicates, and represent independent data points. For example, replicate microbial cultures were grown in separate containers and assayed independently from one another.
Randomization	Randomization was not relevant to this study, as the biological subjects for assays were bulk populations of microbial cells.
Blinding	Blinding was not relevant to this study, as the biological subjects for assays were bulk populations of microbial cells.

Reporting for specific materials, systems and methods

We require information from authors about some types of materials, experimental systems and methods used in many studies. Here, indicate whether each material, system or method listed is relevant to your study. If you are not sure if a list item applies to your research, read the appropriate section before selecting a response.

Materials & experimental systems

n/a	Involved in the study	
☒	☐	Antibodies
☐	☒	Eukaryotic cell lines
☒	☐	Palaeontology
☒	☐	Animals and other organisms
☒	☐	Human research participants
☒	☐	Clinical data

Methods

n/a	Involved in the study	
☒	☐	ChIP-seq
☒	☐	Flow cytometry
☒	☐	MRI-based neuroimaging

Eukaryotic cell lines

Policy information about cell lines

Cell line source(s)	Wild-type Saccharomyces cerevisiae strain CEN.PK2-1D was obtained from EuroSCARF (30000B).
Authentication	The commercial cell line used in this study (i.e., Saccharomyces cerevisiae CEN.PK2-1D) was not independently authenticated by the authors of this study. Engineered yeast strains were authenticated by DNA sequencing of relevant genomic modifications, as described in the Methods section of the study.
Mycoplasma contamination	Yeast cell lines were not tested for mycoplasma contamination.
Commonly misidentified lines (See ICLAC register)	Not applicable.
