# Supplementary material for: Engineering a microbial biosynthesis platform for de novo production of tropane alkaloids
Source: Nat Commun. 2019 Aug 12;10:3634. doi: 10.1038/s41467-019-11588-w (PMC6690885; doi:10.1038/s41467-019-11588-w)
Supplement: Supplementary file 4 — Description of Additional Supplementary Files [file 41467_2019_11588_MOESM4_ESM.docx]

**Description of Additional Supplementary Files**

File Name: Supplementary Data 1
Description: Oligonucleotide and primer sequences used for plasmid and strain construction

File Name: Supplementary Data 2
Description: Plasmids used in this study
