# Supplementary material for: Engineering a microbial biosynthesis platform for de novo production of tropane alkaloids
Source: Nat Commun. 2019 Aug 12;10:3634. doi: 10.1038/s41467-019-11588-w (PMC6690885; doi:10.1038/s41467-019-11588-w)
Supplement: Supplementary file 2 — Peer Review [file 41467_2019_11588_MOESM2_ESM.pdf]

Reviewers' comments:

Reviewer #1 (Remarks to the Author):

The study by Srinivasan and Smolke reports, for the first time, de novo biosynthesis of tropine, a precursor of several medicinally relevant tropane alkaloids. While the reported titer is very low, this nevertheless is a proof-of-concept of production of this class of compounds in a microbial cell factory. The study is well designed and executed and well written. I particularly enjoyed the systematic application of diverse metabolic engineering strategies to improve the production of tropine precursors and then tropine itself.
I have however one major concern with the manuscript: many titers are presented as relative increase rather than absolute values. This, in my view, is a data reduction practice that should be avoided. As the authors had the standards of the compounds, there is no reason for not providing the absolute values.
Otherwise, I have only a couple of minor comments:
Line 80: The authors state that very low titers of NMPy were achieved in Ping et al (ACS Syn Bio, 2019). However I could not find the actual titers obtained in the current study, hence this argument needs to be substantiated.
Figure 2a is identical with Fig1-ModuleI and is unnecessary. Same with Fig5a.

Reviewer #2 (Remarks to the Author):

The manuscript authored by Prashanth Srinivasan and Christina D. Smolke described an engineered yeast platform for production of tropine and derivate cinnamoyltropine. The synthetic strain incorporated over 10 additional genes from different plants and bacterias. The manuscript was well organized and the work was extensively presented especially in the first module part about the metabolic engineering of the precursor. However it’s not clear why the final products’ yields are so low and there is miss linkage between high yield of precursor and low contents of key intermediates in the later steps. Here are a number of important points need to be clearly addressed.
1) The authors put lots of efforts on the improvement of flux from arginine metabolism to putrescine. For example, by overexpressing native yeast gene Arg2p associated with arginine biosynthesis to increase the titers of the precursor. As there are multiple metabolic steps from glutamic acid to arginine, why the author only selected the first step catalyzed by Arg2p for overexpressing. What’s the rationality inside it and how about co-overexpress all the genes in the pathway? In addition in the short pathway from glutamic acid to arginine, L-ornithine is one of the intermediate which is also designed to produce after arginine catalyzed by Car1p. How about cuting short the redundant pathway for directing production of ornithine?
2) About 10 years before, Qian et al. have published for production of putrescine in E coli with over g/L (1.68-24.2g/L) titer (BB, DOI 10.1002/bit.22502) whose metabolic pathway is similar to yeast (doi:10.1371/journal.pone.0034734.g001). Why the authors did not use this kind of simple/single pathway with high-yield engineering strategy in yeast to achieve g/L level of titer instead of complex integration three different pathways with only mg/L yield of putrescine. The engineering of the first part about production of putrescine may need further optimization with more rational pathway design. This g/L of putrescine will also help to get higher yield of final products.
3) In part 2, about the production of NMPy, what’s the exact final titer of NMPy from the strain CSY1243. It’s better to show a time course of titer along with the key precursor putrescine. In addition, by checking the published NMPy yield in yeast from ref 23, the titer ca 20mg/L instead of 2-3mg/L as the authors indicated in the manuscript. The authors need to correct the number and compare with the titer which was achieved in the manuscript.
4) In part 3, to validate enzyme activity and identify potential bottlenecks, the authors monitored the accumulation of NMPy, MPOB, tropinone and tropine and presented the result in fig 4b. But the above metabolites’ yields were not fully covered, like NMPy and MPOB were missed. These datasets need to be incorporated.

5) In line 394-396, about the hypothesis of second mechanism for hygrine production, what's the direct data to support this and how is about the second mechanism. It's better to explain/discuss this well as hygrine does a big by-pass flux leaking in the pathway.
6) As there is stereochemistry related to both the two final products, tropinine and cinnamoyltropine, full sets of NMR data to show the correct structures will be encouraged to have from the purified products in the fermentation strains.
7) With the optimized strains for production of tropinine and cinnamoyltropine, the titers of these final products and key intermediates, like putrescine, NMPy, tropinone, and tropine, are needed to monitor based on the time course and discuss the bottlenecks and how about the consuming of the precursors and intermediates.
8) Overall, as the precursor, putrescine had reach almost 100mg/L, but in the following 5 steps, the final product tropine only got 1.5mg/L. The AbCYP82M3 was generally thought as the potential bottleneck like most other cases in the NP metabolite engineering, but there was no significant increase with additional copy. So the metabolites flux in the synthesized pathway were not fully addressed and need more analysis to figure out the key issue.
9) For the titer caculation of the metabolites, the standard cureves need to be shown in the supportion information.

Reviewer #3 (Remarks to the Author):

The data presented in this manuscript presents a significant step forward in the metabolic engineering tropane alkaloids, a class of pharmaceutically important plant natural products. The authors have utilized a combinatorial approach of both engineering and optimizing plant derived enzymes involved in tropane alkaloid biosynthesis together with the manipulation of endogenous yeast pathways to improve flux and minimize side reactions that reduce target compound production. This is a very nice study that builds on previous achievements from this lab centered around the engineering of plant pathways in microbial hosts. The impact of this study is slightly compromised due to the very recent publication by Ping and co-authors (ACS Synthetic Biology 2019, 8, 257-263) who engineered the production of N-methylpyrrolinium in yeast and E. coli and used a very similar strategy of reducing endogenous aldehyde dehydrogenase activity in yeast to reduce side reactions that reduce target product accumulation.
There are several items for the authors to consider.
1) Figure 2e is a good visual representation of the putrescine produced in the various strains but the authors should consider providing appropriate graphs or tables of the actual quantitative data perhaps in the supplemental data.
2) The authors invested a significant effort to boost the production of putrescine in yeast and the experiments are well performed and the data presentation is clear with appropriate quantification of metabolites. In subsequent sections of the paper the same levels of rigor were not followed. For example, the authors critique the titers of N-methylpyrrolinium achieved in the study by Ping et al., stating on line 80 that they only achieved 2-3 mg/L. This is a false statement as those authors report a titer of almost 18 mg /L following strain optimization. In contrast, absolute titers of putrescine, NMP, 4MAB, and N-methylpyrrolinium achieved in this study are not reported (Fig 3 and S3 and S4), only relative amounts. The manuscript would be strengthened if data presenting absolute quantification of these metabolites were included. Furthermore, it is definitely not appropriate to criticize the titers of NMPy achieved by Ping et al., and then not report the titers achieved in your own work.
3) The authors tested the efficacy of several MPO genes and ultimately found that deleting the c-terminus of MPO1 from Datura metel provided improved production in their system. In relation to this, the authors should be aware that multiple MPO genes are present in higher plants. The sequence obtained from Datura metel from the 1Kp plant database was derived from a leaf library. Tropanes are synthesized in the roots in members of the Solanaceae family and the genes involved in their synthesis are root expressed. Therefore, it is possible that in this case the authors may have selected an MPO gene that has not evolved to optimally utilize NMP, which may limit flux through the system.

This is perhaps a valuable point to add to the discussion.
4) The fact the cinnamoyltropine was formed using EcCS in this yeast system is entirely unexpected and warrants further investigation. The original report of EcCS activity (Plant Physiology 2015, 167: 89-101) stated that recombinant EcCS was not active with tropine (alpha-configuration) as the acyl acceptor, only pseudotropine and methylecgonine (both beta-configuration). DsTRI is reported to only synthesize tropine, not pseudotropine and thus one would predict that a tropane ester would not be formed in this yeast system. Could racemization of tropine be occurring in yeast or does DsTI synthesize small amounts of pseudotropine? How confident are the authors that the product they are detecting is cinnamoyltropine and not cinnamoylpseudotropine? Follow-up experiments are needed to confirm the identity of this tropane ester product. In addition, this final experiment seems to have been rushed, because, as with the data presented in Figure 3, no absolute quantification of the titers of the tropane ester of cinnamate are provided. Surely, it would be typical to quantify the end-point products of a metabolic engineering strategy?
5) The authors should be careful when stating that cinnamoyltropine is a non-natural tropane. There is a single report in the literature that detected cinnamoyltropine in Latua pubiflora via GC-MS (Munoz & Casale 2003, Zeitschrift Fur Naturforschung C-a Journal of Biosciences, 58: 626-628). Although these data have not been independently confirmed, toning down the significance of making cinnamate tropane esters may be appropriate. Certainly, the approach the authors are taking has the potential to make non-natural tropanes, just not sure whether cinnamoyltropine is one of them.
6) Line 546: AbCYP82M3 is likely rate limiting for tropinone biosynthesis, not tropine biosynthesis.

Response to Reviewer Comments

We would like to thank the reviewers for their constructive comments and helpful suggestions for improving our manuscript. The comments were very useful in helping us to strengthen the manuscript, and in particular for identifying bottlenecks and inefficiencies in our engineered pathway. In the sections below, we address each comment made by the reviewers.

Reviewer #1 (Remarks to the Author):

> *The study by Srinivasan and Smolke reports, for the first time, de novo biosynthesis of tropine, a precursor of several medicinally relevant tropane alkaloids. While the reported titer is very low, this nevertheless is a proof-of-concept of production of this class of compounds in a microbial cell factory. The study is well designed and executed and well written. I particularly enjoyed the systematic application of diverse metabolic engineering strategies to improve the production of tropine precursors and then tropine itself.*
>
> *I have however one major concern with the manuscript: many titers are presented as relative increase rather than absolute values. This, in my view, is a data reduction practice that should be avoided. As the authors had the standards of the compounds, there is no reason for not providing the absolute values.*

We thank the reviewer for their kind remarks and their suggestion. Although we had initially felt that fold-changes or relative titers effectively conveyed important differences between samples and strains, we agree that this could be equally, if not more effectively, conveyed through actual titers. Therefore, we have repeated key experiments (the MPO truncation test in Fig. 3c and the strain ladder showing differences in 4MAB acid and NMPy titers between different ALD disruptions in Fig. 3d) with standard curves to collect titer measurements so that all data in the main figures are now reported as absolute values. In addition, we have replaced relative titer data in Fig. 4 (the effect of growth temperature on tropine and hygrine production in Fig. 4c; and the effect of *ALD6* reconstitution on 4MAB acid and hygrine titers, originally Fig. 4e but now in Fig. S10b) with absolute titers, for which we already had titer measurements. We feel that the fold-change comparisons made in the supplementary figures, when paired with absolute titer data presented in the main figures, now presents a more compelling argument for the effect of strain modifications on metabolite fluxes.

> *Otherwise, I have only a couple of minor comments:*
>
> *Line 80: The authors state that very low titers of NMPy were achieved in Ping et al (ACS Syn Bio, 2019). However I could not find the actual titers obtained in the current study, hence this argument needs to be substantiated.*

We thank the reviewer for pointing out this error. We had initially cited an incorrect value for NMPy titers achieved in that paper (specifically, from an earlier strain in the paper), and have corrected this reference in our text accordingly. Although the titers we have achieved in our NMPy-producing strain (CSY1243; 39 mg/L) are roughly two-fold greater than the titers reported by Ping *et al.* (~18 mg/L), likely due to our extensive optimization of putrescine production up to 86 mg/L, our observed conversion of putrescine to NMPy did not appear to be as efficient as in the final strain reported by Ping *et al*, as indicated by the roughly 50% loss of flux from putrescine to NMPy from CSY1235 to CSY1243. We point out this discrepancy in the Discussion section.

Figure 2a is identical with Fig1-ModuleI and is unnecessary. Same with Fig5a.

We thank the reviewer for pointing out this potential redundancy. We felt that Fig. 2a includes some additional details not found in Fig. 1—namely, the distinction between native and heterologous putrescine biosynthetic pathways, as well as the agmatine iminohydrolase (AIH)-catalyzed pathway—which justify its inclusion. We agree that Fig. 5a is entirely redundant and have removed this panel from the revised manuscript.

Reviewer #2 (Remarks to the Author):

The manuscript authored by Prashanth Srinivasan and Christina D. Smolke described an engineered yeast platform for production of tropine and derivate cinnamoyltropine. The synthetic strain incorporated over 10 additional genes from different plants and bacteria. The manuscript was well organized and the work was extensively presented especially in the first module part about the metabolic engineering of the precursor. However it's not clear why the final products' yields are so low and there is miss linkage between high yield of precursor and low contents of key intermediates in the later steps. Here are a number of important points need to be clearly addressed.

1) The authors put lots of efforts on the improvement of flux from arginine metabolism to putrescine. For example, by overexpressing native yeast gene Arg2p associated with arginine biosynthesis to increase the titers of the precursor. As there are multiple metabolic steps from glutamic acid to arginine, why the author only selected the first step catalyzed by Arg2p for overexpressing. What's the rationality inside it and how about co-overexpress all the genes in the pathway? In addition in the short pathway from glutamic acid to arginine, L-ornithine is one of the intermediate which is also designed to produce after arginine catalyzed by Car1p. How about cutting short the redundant pathway for directing production of ornithine?

We thank the reviewer for their useful comments regarding putrescine optimization. Initial gene targets for overproduction of putrescine were selected based on inspection of arginine and polyamine metabolic pathways in the KEGG database, in concert with data reported in a previous study from the Nielsen laboratory (Qin *et al*., *Nat. Commun.* 2015, 8(6):8224) which engineered yeast for ornithine overproduction. In that study, it was found that while overexpression of all four of *ARG5-8* together gave only a 31% increase in ornithine titer, overexpression of *ARG2* gave a 36% improvement. As such, *ARG2* was initially targeted for overexpression due to its much higher individual impact, and *ARG5-8* were kept as contingency targets if putrescine titers were still found to be limiting in later steps of the pathway. Moreover, as *ARG5-8* all feed into the urea cycle, we were concerned that strong overexpression of all of these mitochondrial genes may result in mitochondrial dysfunction, as we had observed with overexpression of *ORT1*. Therefore, we targeted single genes whose individual overexpression was demonstrated previously or which was expected to increase flux to putrescine.

In response to the suggestion for cutting short the redundant pathway, we assume that the reviewer is referring to the urea cycle, where ornithine is converted to arginine and back, and that interrupting the urea cycle at ornithine (so that it is not converted to arginine, i.e., via disruption of *ARG3*) might increase ornithine accumulation. This is possible, but we were concerned that eliminating the urea cycle would lead to a metabolic backpressure or buildup that inhibits ammonia detoxification, or which more generally retards growth.

Overall, we felt that many of these suggestions for further improving putrescine production would be very useful for later strain optimization towards achieving more industrially relevant titers of tropane alkaloid precursors. However, we did not feel that they were strictly necessary for the present proof-of-

concept study, especially given that our later bottleneck analysis in the tropine-producing strain showed that PMT (and not putrescine production) was limiting flux. We have revised the manuscript text to briefly address these points.

2) About 10 years before, Qian et al. have published for production of putrescine in E. coli with over g/L (1.68-24.2g/L) titer (BB, DOI 10.1002/bit.22502) whose metabolic pathway is similar to yeast (doi:10.1371/journal.pone.0034734.g001). Why the authors did not use this kind of simple/single pathway with high-yield engineering strategy in yeast to achieve g/L level of titer instead of complex integration three different pathways with only mg/L yield of putrescine. The engineering of the first part about production of putrescine may need further optimization with more rational pathway design. This g/L of putrescine will also help to get higher yield of final products.

We thank the reviewer for drawing our attention to this prior result. While we were aware of significantly higher putrescine production reported in prokaryotic organisms such as *E. coli* (as in the paper cited above) and *Corynebacterium*, we selected yeast as a host due to its advantages in reconstituting eukaryotic (plant) metabolic pathways. In general, production of primary metabolites, or those close to primary metabolism (i.e., basic amino acids and polyamines) tends to be significantly higher in prokaryotes. As such, we do not feel that the achievement of g/L putrescine titers in *E. coli* necessarily indicates that similar titers are achievable in yeast with similar modifications to metabolism. Moreover, many of the engineering strategies implemented in the *E. coli* paper pointed out by the reviewer were also implemented in our study in yeast—overexpression of ornithine decarboxylase and upstream ornithine and arginine biosynthesis genes, elimination of putrescine-consuming reactions, and elimination of regulatory mechanisms—and yet, putrescine titers still only approached 0.1 g/L in our yeast system. In fact, in the absence of the heterologous putrescine pathways (i.e., with rewiring of the native pathways only), putrescine titers were significantly lower.

While g/L titers for ornithine in yeast were previously achieved in the study by the Nielsen laboratory (Qin *et al.*, *Nat. Commun.* 2015, 8(6):8224), this required extensive rewiring of many modules within yeast metabolism. We had initially planned to implement some of these more extensive metabolic modifications if it was found that putrescine production was limiting downstream tropane alkaloid precursor flux; however, as our later bottleneck testing and time course experiments in the tropine-producing strain showed, putrescine did not appear to be a primary bottleneck limiting tropine production at this development stage. As such, the reviewer's suggestions for more extensive single-pathway rewiring for g/L putrescine production will be extremely useful for future strain optimization efforts towards achieving industrially relevant tropane alkaloid precursor titers. However, we did not feel that it was necessary or within the scope of this proof-of-concept paper. We have revised the manuscript text to briefly address these points.

3) In part 2, about the production of NMPy, what's the exact final titer of NMPy from the strain CSY1243. It's better to show a time course of titer along with the key precursor putrescine. In addition, by checking the published NMPy yield in yeast from ref 23, the titer ca 20mg/L instead of 2-3mg/L as the authors indicated in the manuscript. The authors need to correct the number and compare with the titer which was achieved in the manuscript.

We thank the reviewer for pointing out this omission. Although we had initially felt that relative titers effectively conveyed important differences between samples and strains, particularly for intermediates between putrescine and tropine, we agree that absolute titers for key intermediates are important for drawing conclusions regarding bottlenecks, losses of metabolite fluxes from the pathway, and for making

comparisons to other published work. Therefore, we have repeated relevant experiments for Fig. 3—specifically, the MPO truncation test (3c) and the ALD disruption comparison (3d)—and report absolute titers for the intermediate NMPy and the side product 4MAB acid in the revised manuscript. We have also performed a time course experiment to measure the accumulation of putrescine, NMP, and NMPy in the integrated NMPy-producing strain (CSY1243). Although putrescine titers in the putrescine-producing strain (CSY1235) reached over 80 mg/L, NMPy titers in CSY1243 reached only 39 mg/L, despite nearly complete consumption of putrescine and NMP (Fig. S7), suggesting a significant loss of flux which we discuss further in the Discussion section in the revised manuscript. We also thank the reviewer for pointing out the incorrectly cited NMPy titer from ref. 23. We have corrected this reference in our text and amended the relevant Discussion section accordingly.

> *4) In part 3, to validate enzyme activity and identify potential bottlenecks, the authors monitored the accumulation of NMPy, MPOB, tropinone and tropine and presented the result in fig 4b. But the above metabolites' yields were not fully covered, like NMPy and MPOB were missed. These datasets need to be incorporated.*

We thank the reviewer for this comment. The purpose of the data presented in Fig. 4b was to indicate any differences between cytochrome P450 reductase partners, and not necessarily to identify bottlenecks (which was performed only in subsequent sections). While the reviewer is correct that only the production of tropinone, tropine, and hygrine—i.e., the novel products produced by PYKS + CYP82M3—is shown in the main text (Fig. 4b), the consumption of NMPy and MPOB with expression of PYKS and CYP82M3 is shown in the Supplementary Text (originally Fig. S9b, now Fig. S8b). We agree that our reference to this data in the text was unclear, and have amended the relevant section in the Results to more explicitly indicate where the relevant metabolites are quantified. We have now performed additional experiments to identify metabolite bottlenecks in the final tropine-producing strain, and have included titer measurements for intermediates between putrescine and tropine in the form of time course data in Fig. 4f and S13.

> *5) In line 394-396, about the hypothesis of second mechanism for hygrine production, what's the direct data to support this and how is about the second mechanism. It's better to explain/discuss this well as hygrine does a big by-pass flux leaking in the pathway.*

We thank the reviewer for this suggestion and agree that the magnitude of the hygrine leak flux merits further characterization. Our initial hypothesis for the second mechanism of hygrine production—spontaneous condensation with acetate—was based on our observation from Fig. 4b that hygrine accumulation was detected in the NMPy-producing strain (CSY1243) in the absence of PYKS and CYP82M3 expression.

To better characterize the co-substrate which condenses with NMPy to produce hygrine, we performed feeding experiments in which we assayed different combinations of NMPy-producing and –non-producing strains with growth media supplemented with acetate and acetoacetate. We observed no hygrine accumulation in cultures of wild-type yeast fed either acetate or acetoacetate. In cultures of NMPy-producing strains, we observed hygrine accumulation with either acetate or acetoacetate supplementation in the media, but not in the absence of either. However, while we were able to detect hygrine accumulation when NMPy was incubated with acetoacetate alone (i.e., a 'cell-free' reaction), we were not able to detect hygrine when NMPy was incubated with acetate in the absence of cells, indicating that direct NMPy condensation with acetate may not account for hygrine production in the NMPy-producing strain. Rather, we suspect that either acetate or acetoacetate supplementation increases

intracellular levels of an unknown keto-metabolite—potentially acetoacetyl-CoA or acetoacetate itself, both of which are native yeast metabolites (Shimizu, Nagai, and Katsuki, *J Biochem* 1974, (1):69-76)—which then condenses with NMPy to form hygrine. We feel that the further experimentation required to establish the specific identity of the condensation co-substrate for this side reaction *in vivo* is beyond the scope of this study. However, we have amended the text and Fig. 4a to reflect the uncertainty in the identity of the keto-substrate for hygrine production. Moreover, we believe that our subsequent efforts to abolish acetate auxotrophy and eliminate the need for acetate feeding are still justified, since our feeding data indicates that acetate feeding increases hygrine accumulation.

> *6) As there is stereochemistry related to both the two final products, tropine and cinnamoyltropine, full sets of NMR data to show the correct structures will be encouraged to have from the purified products in the fermentation strains.*

We thank the reviewer for these suggestions. While we agree that there is stereochemistry associated with the final product tropine, we did not feel it was necessary to perform further characterization of the *de novo* produced tropine. It is well established in the literature through both *in vitro* and *in vivo* studies that tropinone reductase I and II show mutually exclusive stereospecificities (Nakajima *et al. PNAS* 1993, 90:9591-9595; Nakajima *et al. J. Biol Chem.* 1994, 269:11695-11698; Nakajima *et al. J. Biol Chem.* 1998, 274(23):16563-16568; Nakajima *et al. PNAS* 1998, 95:4876-4881). In particular, it has been previously demonstrated that TRI from *D. stramonium* shows exclusive specificity for the alpha isomer of tropine from tropinone (Portsteffen *et al. Phytochemistry* 1994, 37(2):391-400). We believe that this substantial body of evidence in the literature for the α-specificity of DsTRI is sufficient to identify our *de novo* produced tropine as the alpha isomer. We have, however, performed additional characterization of the stereospecificity of the subsequent cocaine synthase-catalyzed reaction producing cinnamoyltropine from α-tropine.

We were able to procure a custom-synthesized standard of cinnamoyl-3α-tropine from a commercial synthesis company (Aurora Fine Chemicals LLC). To confirm its stereochemistry and identity, we performed NMR on the synthesized cinnamoyltropine, as well as on the constituent substrates trans-cinnamic acid, tropine, and pseudotropine (β-tropine). Spectral comparison indicated that the synthesized standard was in fact the ester of trans-cinnamic acid and tropine, not pseudotropine (Fig. S14-S17). Using a standard curve, we determined that *de novo* production of cinnamoyltropine in our engineered strain reached titers of 6 μg/L, which was too low to feasibly produce and purify a sufficient quantity for NMR. Therefore, we instead demonstrated that both the retention time and complete mass fragmentation pattern of the *de novo* produced cinnamoyltropine are identical to that of the standard by LC-MS/MS, including the primary m/z^+ 272 → 124.1 transition and all secondary transitions (m/z^+ 272 → 67.2, 77.2, 93.2, 131.2).

We have also performed additional feeding experiments to provide further support for cocaine synthase activity on the α isomer of tropine (Fig. 5b). We demonstrate that wild-type yeast expressing only EcCS, At4CL5, and AtPAL1 can produce cinnamoyltropine when fed a commercial standard of pure α-tropine (Santa Cruz Biotechnology). We also confirmed by communication with the supplier and by NMR (Fig. S16, S17) that our commercial α-tropine standard was free of pseudotropine contamination, and that the only contaminant indicated by the supplier was water. This indicates that when expressed in yeast, EcCS does indeed have acyltransferase activity on the α isomer of tropine.

> *7) With the optimized strains for production of tropine and cinnamoyltropine, the titers of these final products and key intermediates, like putrescine, NMPy, tropinone, and tropine, are needed to monitor*

based on the time course and discuss the bottlenecks and how about the consuming of the precursors and intermediates.

We thank the reviewer for this helpful suggestion, and we agree that time course data of intermediate and product titers over time would provide a much more comprehensive picture of key bottlenecks in the pathway. Accordingly, we have performed time course experiments for the final tropine-producing strain and quantified the intermediates putrescine, NMP, NMPy, hygrine, tropinone, and tropine over the course of the fermentation (Fig. 4f and S13). Based on this analysis, we were able to determine that a significant quantity of produced NMPy remained unconverted to tropine at the end of the fermentation, suggesting that the PYKS-catalyzed condensation of NMPy with malonyl-CoA might remain the primary bottleneck limiting flux in this strain, even with two copies of *AbPYKS* in CSY1251. We discuss these findings and their implications in the Discussion section. However, as production of cinnamoyltropine was so much lower than tropine (3 orders of magnitude, 6 μg/L vs. 6 mg/L), we did not feel that a time course of the cinnamoyltropine-producing strain would provide any additional information regarding bottlenecks that would not be revealed in the time course of the tropine-producing strain.

8) Overall, as the precursor, putrescine had reach almost 100mg/L, but in the following 5 steps, the final product tropine only got 1.5mg/L. The AbCYP82M3 was generally thought as the potential bottleneck like most other cases in the NP metabolite engineering, but there was no significant increase with additional copy. So the metabolites flux in the synthesized pathway were not fully addressed and need more analysis to figure out the key issue.

We thank the reviewer for pointing out this discrepancy as an area for further exploration. To this end, we have included several additional experiments intended to further improve tropine titers and elucidate remaining bottlenecks or leak fluxes.

First, we performed a metabolic bottleneck detection experiment in the tropine-producing strain CSY1249 in which we expressed an additional copy of each heterologous biosynthetic enzyme between putrescine and tropine, and examined the impact on tropine titers. We observed that an additional copy of *PMT* provided an over two-fold increase in tropine titers, with less dramatic improvements provided by an additional copy of *AbPYKS*, suggesting that PMT expression was severely limiting flux through this pathway. We constructed an optimized strain with additional copies of the flux-limiting *PMT* and *PYKS* genes, leading to an over two-fold increase in tropine titer in CSY1251 (Fig. 4d).

Second, we performed carbon source optimization and identified that glycerol supplementation in synthetic defined media significantly increases tropine production, and comment on potential reasons for this improvement in the Discussion section.

Finally, as described above, we performed time course experiments for the final tropine-producing strain CSY1251 using both conventional low-density cultures (i.e., starting from a very low initial OD) as well as high-density cultures, and observed that tropine titers reached ~6 mg/L in the high-density cultures—a four-fold improvement from our initial reported tropine titers. Moreover, titers of the side product hygrine reached 10 mg/L, while about 30 mg/L of NMPy remained unreacted at the end of the fermentation. This indicated that low flux of NMPy into MPOB and tropinone was severely limiting tropine production, despite an additional copy of *AbPYKS* present in this strain. As such, we inferred that some aspect of the PKS enzyme other than expression level might now be the primary bottleneck, such as co-substrate (malonyl-CoA) availability or poor inherent activity in yeast. For instance, a recent study established that an engineered/evolved variant of AbPYKS shows significantly greater activity in yeast (Wrenbeck, E. *et al. ACS Syn. Biol.* 2019, 8(3):474-481). However, we feel that further optimization of PKS and/or malonyl-CoA availability would be more appropriate for follow-up studies.

We feel that our time course experiments now better identify the source of the discrepancies and inefficiencies between initial putrescine and final tropine titers. Of the 80 mg/L putrescine that was produced in CSY1235, only about half (<40 mg/L) is converted to NMPy, as indicated by our NMPy time course data in CSY1243, suggesting some loss of flux between putrescine and NMPy which we address in the Discussion section. Of this, 30 mg/L NMPy remains unconsumed at the end of the time course in the tropine-producing strain CSY1251, suggesting inefficient utilization of NMPy and explaining why tropine and hygrine are significantly lower than would be expected. We feel that optimization of these flux losses would be appropriate for a subsequent study focused on improving tropine titers towards industrially relevant levels, but that current optimization and troubleshooting efforts are sufficient for this initial proof-of-concept paper.

9) For the titer calculation of the metabolites, the standard curves need to be shown in the supporting information.

We thank the reviewer for pointing out this omission. We have included representative standard curves for all quantified metabolites in Supplementary Figure 18.

Reviewer #3 (Remarks to the Author):

The data presented in this manuscript presents a significant step forward in the metabolic engineering tropane alkaloids, a class of pharmaceutically important plant natural products. The authors have utilized a combinatorial approach of both engineering and optimizing plant derived enzymes involved in tropane alkaloid biosynthesis together with the manipulation of endogenous yeast pathways to improve flux and minimize side reactions that reduce target compound production. This is a very nice study that builds on previous achievements from this lab centered around the engineering of plant pathways in microbial hosts. The impact of this study is slightly compromised due to the very recent publication by Ping and co-authors (ACS Synthetic Biology 2019, 8, 257-263) who engineered the production of N-methylpyrrolinium in yeast and E. coli and used a very similar strategy of reducing endogenous aldehyde dehydrogenase activity in yeast to reduce side reactions that reduce target
product accumulation.
There are several items for the authors to consider.
1) Figure 2e is a good visual representation of the putrescine produced in the various strains but the authors should consider providing appropriate graphs or tables of the actual quantitative data perhaps in the supplemental data.

We thank the reviewer for this helpful suggestion regarding data presentation clarity. We have updated the heatmap in Fig. 2e with the actual putrescine titer values ± s.d., so that absolute titers can now be more easily compared. We have also included all data underlying graphs, including this heatmap, in the source data file.

2) The authors invested a significant effort to boost the production of putrescine in yeast and the experiments are well performed and the data presentation is clear with appropriate quantification of metabolites. In subsequent sections of the paper the same levels of rigor were not followed. For example, the authors critique the titers of N-methylpyrrolinium achieved in the study by Ping et al., stating on line 80 that they only achieved 2-3 mg/L. This is a false statement as those authors report a

titer of almost 18 mg /L following strain optimization. In contrast, absolute titers of putrescine, NMP, 4MAB, and N-methylpyrrolinium achieved in this study are not reported (Fig 3 and S3 and S4), only relative amounts. The manuscript would be strengthened if data presenting absolute quantification of these metabolites were included. Furthermore, it is definitely not appropriate to criticize the titers of NMPy achieved by Ping et al., and then not report the titers achieved in your own work.

We thank the reviewer for drawing our attention to this omission. Although we had initially felt that relative titers effectively conveyed important differences between samples and strains, particularly for intermediates between putrescine and tropine, we agree that absolute titers for key intermediates are important for drawing conclusions regarding bottlenecks, losses of flux from the pathway, and particularly for making comparisons to the literature. Therefore, we have repeated relevant experiments for Fig. 3—specifically, the MPO truncation test (Fig. 3c) and the ALD disruption comparison (Fig. 3d)—and reported absolute titers for the intermediate NMPy and the side product 4MAB acid. We have also performed a time course experiment to measure the accumulation of putrescine, NMP, and NMPy in the integrated NMPy-producing strain (CSY1243). We also thank the reviewer for pointing out the incorrectly cited NMPy titer from ref. 23. We have corrected this reference in our text and amended the relevant Discussion section accordingly.

3) The authors tested the efficacy of several MPO genes and ultimately found that deleting the c-terminus of MPO1 from Datura metel provided improved production in their system. In relation to this, the authors should be aware that multiple MPO genes are present in higher plants. The sequence obtained from Datura metel from the 1Kp plant database was derived from a leaf library. Tropanes are synthesized in the roots in members of the Solanaceae family and the genes involved in their synthesis are root expressed. Therefore, it is possible that in this case the authors may have selected an MPO gene that has not evolved to optimally utilize NMP, which may limit flux through the system. This is perhaps a valuable point to add to the discussion.

We are grateful to the reviewer for bringing this distinction between leaf- and root-expressed MPO variants to our attention, as we were not aware of the source of our identified *Datura* MPO ortholog. We agree that it would be prudent for further optimization efforts to screen additional root-specific MPO variants, and comment on this distinction in the Discussion section.

4) The fact the cinnamoyltropine was formed using EcCS in this yeast system is entirely unexpected and warrants further investigation. The original report of EcCS activity (Plant Physiology 2015, 167: 89-101) stated that recombinant EcCS was not active with tropine (alpha-configuration) as the acyl acceptor, only pseudotropine and methylecgonine (both beta-configuration). DsTRI is reported to only synthesize tropine, not pseudotropine and thus one would predict that a tropane ester would not be formed in this yeast system. Could racemization of tropine be occurring in yeast or does DsTRI synthesize small amounts of pseudotropine? How confident are the authors that the product they are detecting is cinnamoyltropine and not cinnamoylpseudotropine? Follow-up experiments are needed to confirm the identity of this tropane ester product.

We thank the reviewer for pointing out the unexpected nature of this result and the importance of stereochemistry for this product, and we agree that further characterization of this product was required. We do not believe that spontaneous racemization of tropine to pseudotropine occurs, as the requisite oxidation-reduction through the tropinone intermediate requires the high group-transfer potential supplied by the enzyme cofactor NADPH/$NADP^+$. As the reviewer pointed out, tropinone reductase I (and

specifically, TRI from *D. stramonium*) has well-established stereospecificity for the α-isomer of tropine only (Portsteffen *et al. Phytochemistry* 1994, 37(2):391-400). As such, we do not believe that the observed *de novo* cinnamoyltropine is derived from trace pseudotropine produced enzymatically or spontaneously in yeast. Therefore, we have performed additional experiments and characterization of the *de novo* cinnamoyltropine product to support our claims.

First, we procured a custom-synthesized standard of cinnamoyl-3α-tropine from a commercial synthesis company (Aurora Fine Chemicals LLC). To confirm its stereochemistry and identity, we performed NMR on the synthesized cinnamoyltropine, as well as on the constituent substrates trans-cinnamic acid, tropine, and pseudotropine (β-tropine). Spectral comparison indicated that the synthesized standard was in fact the ester of trans-cinnamic acid and tropine, not pseudotropine (Fig. S14-S17). We demonstrated that both the retention time and complete mass fragmentation pattern of the *de novo* yeast-produced cinnamoyltropine were identical to that of the standard by LC-MS/MS, including the primary 272 → 124.1 peak and all secondary peaks (m/z^+ 67.2, 77.2, 93.2, 131.2).

To provide further support for the unexpected activity of EcCS on α-tropine, we have included additional feeding experiments presented in Fig. 5b. We demonstrate that wild-type yeast expressing only EcCS, At4CL5, and AtPAL1 can produce cinnamoyltropine when fed a commercial standard of pure α-tropine (Santa Cruz Biotechnology). We also confirmed by communication with the supplier and by NMR that our commercial α-tropine standard was free of pseudotropine contamination (Fig. S16, S17), and that the only contaminant detected by the supplier was water. This indicates that when expressed in yeast, EcCS does indeed have acyltransferase activity on the α isomer of tropine. However, the activity on this isomer is so low as to be considered trace, as indicated by the 3 order-of-magnitude difference in cinnamoyltropine titers (6 μg/L) relative to tropine production (6 mg/L), which may explain why it was potentially undetectable in previous studies characterizing EcCS.

> *In addition, this final experiment seems to have been rushed, because, as with the data presented in Figure 3, no absolute quantification of the titers of the tropane ester of cinnamate are provided. Surely, it would be typical to quantify the end-point products of a metabolic engineering strategy?*

We thank the reviewer for pointing out this omission. At the time of our initial submission, we lacked a standard of cinnamoyltropine and thus were forced to use littorine, a structurally similar compound, to estimate its *de novo* titer. As described above, we have since acquired a custom-synthesized standard of cinnamoyltropine from a commercial synthesis company (Aurora Fine Chemicals LLC). We have accordingly included titer quantification data with a standard curve in the main text and supplemental information, respectively.

> *5) The authors should be careful when stating that cinnamoyltropine is a non-natural tropane. There is a single report in the literature that detected cinnamoyltropine in Latua pubiflora via GC-MS (Munoz & Casale 2003, Zeitschrift Fur Naturforschung C-a Journal of Biosciences, 58: 626-628). Although these data have not been independently confirmed, toning down the significance of making cinnamate tropane esters may be appropriate. Certainly, the approach the authors are taking has the potential to make non-natural tropanes, just not sure whether cinnamoyltropine is one of them.*

We thank the reviewer for bringing this paper to our attention, as we were not previously aware of any reports of cinnamoyltropine production in plants. We have therefore adopted the term 'non-canonical' tropane alkaloid, rather than non-natural tropane alkaloid, to refer to cinnamoyltropine in the text, as we believe the former term better reflects the fact that cinnamate tropane esters are not conventionally found in naturally-occurring medicinal tropane alkaloid-producing plants.

6) Line 546: AbCYP82M3 is likely rate limiting for tropinone biosynthesis, not tropine biosynthesis.

We thank the reviewer for pointing out this error, and have corrected the text in the revised manuscript.

Reviewers' comments:

Reviewer #1 (Remarks to the Author):

The authors have addressed all the comments. The additional experiments on elucidating the flux controlling steps of the pathway further strengthen the manuscript.

Reviewer #2 (Remarks to the Author):

The revised manuscript authored by Prashanth Srinivasan and Christina D. Smolke reported ca 4-fold increased tropine production titers from 1.53 to 6.0 mg/L with more metabolic enginerring work and optimization of media. It's interested that the titers of final target TA, cinnamoyltropine was still around 6.0 ug/L from current strain CSY1250 which was 5.8 ug/L from last version of strain CSY1282. But the mauscript was well presented with more extensive studies. By comparing the two versions and the reviewers' comments, here are some points would like the author to address.

1) As mentioned above, why the titiers of cinnamoyltropine did not increase as the comparing level of tropine from different strains. And also the yields are three-order of magnitude different between tropine and cinnamoyltropine. Is that because the cinnamoyl-CoA module limited the precursor supply from Module III to IV?
2) The authors explained in the response letter why current ME design strategy in yeast was chosen for production of putrescine in Module I. But for the production of NMPy in module II, the detection of 4MAB was discontinued. In the first half part, the 4MAB and NMP were both checked for evaluation the strains for production of NMPy. But in the last half part about the aldehyde dehydrogenases disruptions, only NMPy was measured. As the (bio)chemical conversions of 4MAB to NMPy and 4MAB to 4MAB acid are both exited in the system, it's better to have both 4MAB and NMPy measured as indicated in figure 3d in the first submitted version while the 4MAB is missing in current figure 3d.
3) About the side reaction of 4MAB acid from 4MAB, the authors stated that ALD2 and ALD3 contributed majority of 4MAB acid production from the results of Hfd1p and ald4p-ald6p discruption strain (less than half of the produced 4MAB acid) and ALD-null strain (completely eliminated the 4MAB acid). Since there is not only deletion of ALD2 and ALD3 strain data, is it proper to have that strong conclusion or if it's possible that there is a synergistic effect in the ALD-null strain?
4)In another side reaction of hygrine, the authors tried new feeding experiments based on the reviewer's comment and had new hypothesis that NMPy reacted with keto-metabilote instead of directly with acetate as indicated in the first version. This proposal makes more sense. But in the discussion paragraph from line 404-416, it's better have the exact titers of hygrine in the series of feeding experiments to comparing to the previous number as indicated in line 396 from the constructed strain.
5)As both the reviewer #2 and #3 raised about the sterochemistry issue of cinnamoyltropine generated from the constructed strain. Even the author confirmed that the custom-synthesized cinnamoyltropine is alpha form based on NMR analysis, but the linked evidence from the yeast product to synthesized standard is purely based on LC-MS/MS. From the similar reported cinnamoyltropine biosynthesis by EcCS in yeast in ref #46 for the beta form, the isomerization was also proposed and the LC/MS results could not provide strong evidence about the absolute sterochemistry. In addition, it's better to have wider scope of MS/MS spectra in figure 5a instead of right cut at the edge of m/z 272 point.

Reviewer #3 (Remarks to the Author):

The authors have addressed all of the concerns of this reviewer. This is an important study and I hope to see it published soon.

Response to Reviewer Comments

We would like to thank the reviewers for their constructive comments and suggestions for further improving our revised manuscript. In the sections below, we address each comment made by the reviewers.

Reviewer #1 (Remarks to the Author):

The authors have addressed all the comments. The additional experiments on elucidating the flux controlling steps of the pathway further strengthen the manuscript.

We thank the reviewer for their supportive remarks, and agree that the suggested additional experiments have produced a more convincing narrative.

Reviewer #2 (Remarks to the Author):

The revised manuscript authored by Prashanth Srinivasan and Christina D. Smolke reported ca 4-fold increased tropine production titers from 1.53 to 6.0 mg/L with more metabolic engineering work and optimization of media. It's interested that the titers of final target TA, cinnamoyltropine was still around 6.0 ug/L from current strain CSY1250 which was 5.8 ug/L from last version of strain CSY1282. But the manuscript was well presented with more extensive studies. By comparing the two versions and the reviewers' comments, here are some points would like the author to address.

1) As mentioned above, why the titers of cinnamoyltropine did not increase as the comparing level of tropine from different strains. And also the yields are three-order of magnitude different between tropine and cinnamoyltropine. Is that because the cinnamoyl-CoA module limited the precursor supply from Module III to IV?

We thank the reviewer for pointing out the need for clarification regarding cinnamoyltropine titers. At the time of our initial manuscript submission, we lacked an authentic standard for cinnamoyltropine; as such, we had estimated the titers of the *de novo* cinnamoyltropine product based on a standard curve of littorine, a structurally related tropane alkaloid (we indicated this in the original manuscript). For the revised submission, we procured a custom-synthesized standard of cinnamoyltropine and used this authentic standard for *de novo* product quantification. Therefore, the original cinnamoyltropine titer (5.8 μg/L) may have been overestimated due to differences in the ionizability of littorine and cinnamoyltropine, potentially masking any increase in cinnamoyltropine titer from CSY1250 to CSY1282. We believe that this difference in the chemical standard used for product quantification at least partially accounts for the perceived lack of commensurate improvement in cinnamoyltropine titers with tropine optimization.

Moreover, we believe that the low affinity of EcCS for α-tropine, rather than the phenylpropanoid module, is the primary factor limiting conversion to cinnamoyltropine. Although this data is not shown in the manuscript (as we believed it to be extraneous for demonstration of *de novo* cinnamoyltropine biosynthesis), feeding additional 0.2 mM cinnamic acid to strain CSY1282 expressing *AtPAL1*, *At4CL5*, and *EcCS* provided a marginal improvement in cinnamoyltropine production, whereas feeding additional 0.5 mM α-tropine resulted in a more dramatic (~3-fold) increase in titer (see Figure A below). This suggests that α-tropine is limiting flux, likely due to the inherently low affinity of EcCS for α-tropine. We believe that additional optimization of the production of this non-canonical product, which will likely require enzyme engineering, is beyond the scope of this paper.

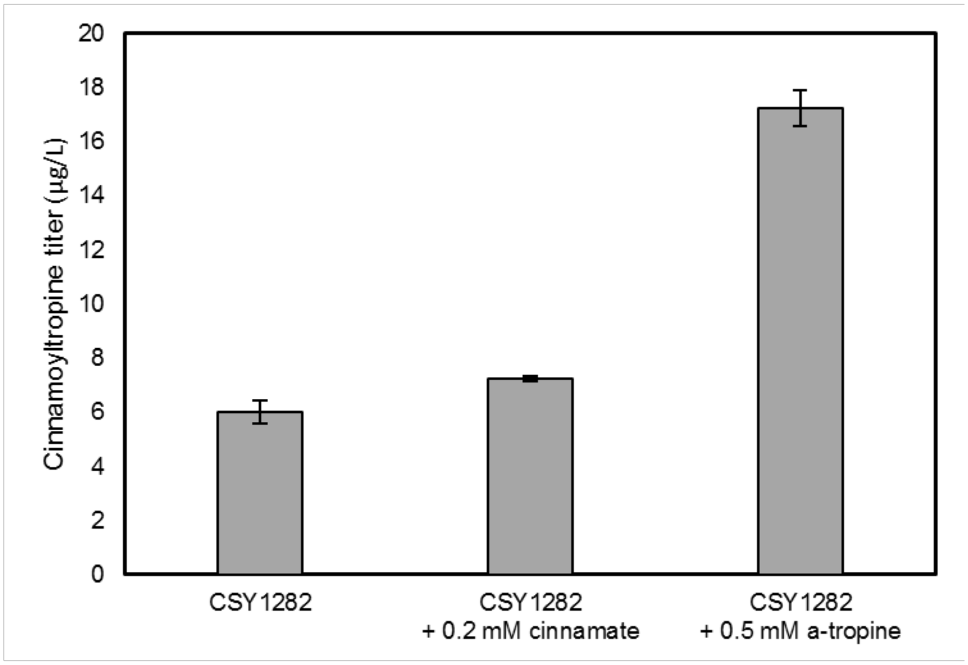

Figure A. Effect of substrate feeding on cinnamoyltropine production in CSY1282.

2) The authors explained in the response letter why current ME design strategy in yeast was chosen for production of putrescine in Module I. But for the production of NMPy in module II, the detection of 4MAB was discontinued. In the first half part, the 4MAB and NMP were both checked for evaluation the strains for production of NMPy. But in the last half part about the aldehyde dehydrogenases disruptions, only NMPy was measured. As the (bio)chemical conversions of 4MAB to NMPy and 4MAB to 4MAB acid are both exited in the system, it's better to have both 4MAB and NMPy measured as indicated in figure 3d in the first submitted version while the 4MAB is missing in current figure 3d.

We thank the reviewer for pointing out this omission. We agree that 4MAB quantification data would be useful in better illustrating the impact of ALD disruptions on the flux balance between NMPy and 4MAB acid. Since the trend in 4MAB levels across the ALD disruptions resembled the trend in NMPy levels, and since we do not wish to over-crowd Figure 3d with a third axis/dataset, we have instead included the relevant 4MAB quantification data as Figure S7 in the Supplementary Data file. We believe that this additional figure, taken together with Figure 3d, now provides a complete picture of the effect of ALD disruptions on the main and side reactions.

3) About the side reaction of 4MAB acid from 4MAB, the authors stated that ALD2 and ALD3 contributed majority of 4MAB acid production from the results of Hfd1p and ald4p-ald6p disruption strain (less than half of the produced 4MAB acid) and ALD-null strain (completely eliminated the 4MAB acid). Since there is not only deletion of ALD2 and ALD3 strain data, is it proper to have that strong conclusion or if it's possible that there is a synergistic effect in the ALD-null strain?

We thank the reviewer for suggesting this possibility. We agree that there is potential for a synergistic effect from deletion of all six ALD genes on the 4MAB acid product. Therefore, we have reworded the relevant sections in the manuscript to reflect this possibility.

4) In another side reaction of hygrine, the authors tried new feeding experiments based on the reviewer's comment and had new hypothesis that NMPy reacted with keto-metabolite instead of

directly with acetate as indicated in the first version. This proposal makes more sense. But in the discussion paragraph from line 404-416, it's better have the exact titers of hygrine in the series of feeding experiments to comparing to the previous number as indicated in line 396 from the constructed strain.

We thank the reviewer for pointing out this omission, and we agree that titer data for the feeding experiments would be useful in drawing more quantitative conclusions when paired with the qualitative LC-MS/MS data in Fig. S10 (previously Fig. S9). We have therefore provided hygrine titer values for each of the feeding conditions in the relevant results section. These values corroborate that approximately 15% of the hygrine production observed with plasmid-based overexpression of *AbPYKS* and *AbCYP82M3* in Fig. 4b is derived from NMPy condensation with a keto-metabolite derived from fed acetate (~110 μg/L for trace *vii* in Fig. S10, versus 775-900 μg/L with *AbPYKS* and *AbCYP82M3* in Fig. 4b). Moreover, the observation that acetoacetate supplementation greatly increased hygrine accumulation in the NMPy-producing strains (Fig. S10, trace *v* vs. *vi*; trace *vii* vs. *viii*), and that acetoacetate (but not acetate) incubation with NMPy led to hygrine accumulation (Fig. S10, trace ix vs. x), lends further support to our hypothesis that acetoacetate is one possible endogenous keto-metabolite which can condense with NMPy.

5) As both the reviewer #2 and #3 raised about the stereochemistry issue of cinnamoyltropine generated from the constructed strain. Even the author confirmed that the custom-synthesized cinnamoyltropine is alpha form based on NMR analysis, but the linked evidence from the yeast product to synthesized standard is purely based on LC-MS/MS. From the similar reported cinnamoyltropine biosynthesis by EcCS in yeast in ref #46 for the beta form, the isomerization was also proposed and the LC/MS results could not provide strong evidence about the absolute stereochemistry. In addition, it's better to have wider scope of MS/MS spectra in figure 5a instead of right cut at the edge of m/z 272 point.

We thank the reviewer for raising this point regarding clarification of stereochemistry. Since CSY1282 produced cinnamoyltropine at titers of only 6 μg/L, it was not feasible to grow a large enough volume of culture (100's of liters) and then purify sufficient cinnamoyltropine from the culture medium (>1-2 mg) to perform NMR on the *de novo* product. Therefore, we felt that the most effective and compelling way to support our conclusions regarding the identity of the *de novo* product was to procure a custom-synthesized authentic standard, confirm the alpha stereochemistry of the standard by NMR, and then demonstrate that both the retention time and MS/MS fragmentation spectrum of the standard matches that of the *de novo* product.

As the reviewer pointed out, the authors of ref #46 suggested that isomerization of 3β-tropine in the culture medium might account for the double peak observed in that paper (but provided no evidence of this occurring). However, as we discussed in our original response letter, we do not believe that spontaneous racemization of tropine/pseudotropine occurs, as the requisite oxidation-reduction through the tropinone intermediate requires the high group-transfer potential supplied by the enzyme cofactor $NADPH/NADP^+$, and is therefore highly thermodynamically unfavorable as a spontaneous process.

Although we did not have an authentic cinnamoyl-3β-tropine standard with which to compare retention times to the cinnamoyl-3α-tropine standard, we noticed that the m/z 272 → 124 peak produced when wild-type yeast expressing *AtPAL1*, *At4CL5*, and *EcCS* were fed β-tropine showed a slightly lower retention time (3.617 min) compared to the 272 → 124 peak produced when the same strain was fed α-tropine (3.684 min), indicating that the two isomers of cinnamoyltropine can be distinguished based on a slight retention time shift (see Figure B below). That the retention time of the cinnamoyltropine peak produced by our engineered strain CSY1282 is consistent with that of the cinnamoyl-3α-tropine standard (Fig. 5b; 3.684 min) and not with that of the strain fed β-tropine (Fig. B below, 3.617 min) further supports our conclusions that our engineered strain is producing the α-isomer of cinnamoyltropine from α-tropine. Furthermore, we observed only a single peak for both the cinnamoyltropine standard and *de novo* product, in contrast to the double peak observed in ref #46 that led the authors to suspect the co-

existence of both isomers. In addition, the relative retention time shift we observed between the α and β isomers of cinnamoyltropine (Fig. B) is consistent with the relative separation between the double peaks observed by the authors in ref #46. If both the α and β isomers were present in our cultures, we would have observed a similar double peak (i.e., the superimposition of traces *i* and *ii* in Fig. B below). The absence of such a double peak in our cultures provides additional support that isomerization of tropine to pseudotropine is not responsible for the observed *de novo* cinnamoyltropine product, and that our engineered strain is producing primarily the alpha isomer of cinnamoyltropine. Regarding the presentation of MS/MS spectra in Figure 5a, our product ion method scanned for fragments of m/z up to 500, but we observed no peaks above the parent mass of m/z = 272 and therefore showed only the portion of the spectrum up to the parent mass. However, we agree that showing a wider spectrum would be useful to indicate the presence (or in this case, absence) of additional peaks or products. Therefore, we have updated Fig. 5a to show the complete mass spectra up to m/z = 500.

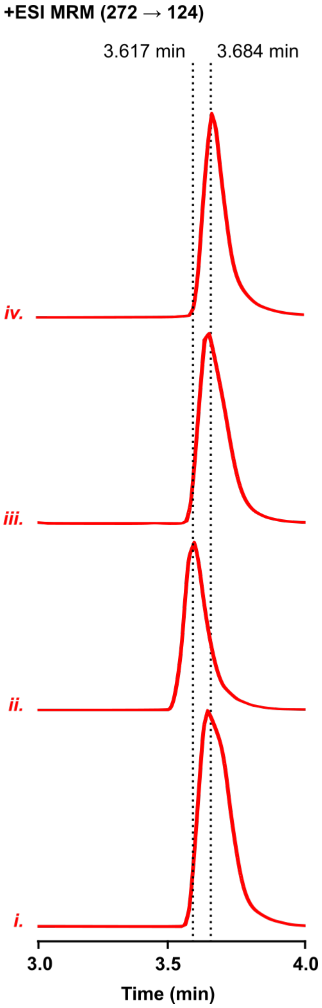

Figure B. Identification of alpha and beta cinnamoyltropine isomers based on retention time. Traces: (i) CEN.PK2 expressing *AtPAL1*, *At4CL5*, and *EcCS* fed 0.5 mM α-tropine; (ii) CEN.PK2 expressing *AtPAL1*, *At4CL5*, and *EcCS* fed 0.5 mM β-tropine; (iii) CSY1282; (iv) authentic cinnamoyl-3α-tropine standard.

Reviewer #3 (Remarks to the Author):

> *The authors have addressed all of the concerns of this reviewer. This is an important study and I hope to see it published soon.*

We thank the reviewer for their supportive remarks.

REVIEWERS' COMMENTS:

Reviewer #2 (Remarks to the Author):

Overall speaking the authors have addressed / explained most of the comments raised. Especially with more quantitative data incorprated, this version is in much better shape. Even there is distinct result / conclusion between current study and the ref #46, the reviewer understands the authors have tried their best to claim the conclusion due to unrealistic enough amount of target compound from current strain. The reviewer expects the authors to have more direct evdence to confirm their conclusion once higher titers engineering starin is in hands in their future study. As there was a paper by Ping et al., published this month reporting both alpha and beta forms of tropine biosynthesis in Anisodus acutangulus and synthetic biology study in yeast (ACS Synth. Biol. DOI: 10.1021/acssynbio.9b00152). The authors should update the literature cited in the manuscript and are encoraged to add some comparsion and discussion about that. In the meanwhile, since the ref #23 by Ping et al. has been on line for almost half a year, it better to delete the words “During the preparation of this manuscript,” in line 595 to low the tone and emphasize how importance about the field which attracts more attentions worldwide from different angles of views.

Response to Reviewer Comments

We would like to thank the reviewers for their constructive comments and suggestions for further improving our revised manuscript. In the sections below, we address each comment made by the reviewers.

Reviewer #2 (Remarks to the Author):

> *Overall speaking the authors have addressed / explained most of the comments raised. Especially with more quantitative data incorporated, this version is in much better shape. Even there is distinct result / conclusion between current study and the ref #46, the reviewer understands the authors have tried their best to claim the conclusion due to unrealistic enough amount of target compound from current strain. The reviewer expects the authors to have more direct evidence to confirm their conclusion once higher titers engineering strain is in hands in their future study.*

We thank the reviewer for their supportive comments and recommendations regarding future product characterization. We agree that once higher-producing strains are established, it will be prudent to perform direct structural characterization of the produced cinnamoyltropine.

> *As there was a paper by Ping et al., published this month reporting both alpha and beta forms of tropine biosynthesis in Anisodus acutangulus and synthetic biology study in yeast (ACS Synth Biol. DOI: 10.1021/acssynbio.9b00152). The authors should update the literature cited in the manuscript and are encouraged to add some comparison and discussion about that.*

We thank the reviewer for their suggestion to update the cited literature. We have included references to this most recent study in the Introduction and Discussion sections, as well as a comparison of the published work to our own results in the Discussion.

> *In the meanwhile, since the ref #23 by Ping et al. has been on line for almost half a year, it better to delete the words "During the preparation of this manuscript," in line 595 to low the tone and emphasize how importance about the field which attracts more attentions worldwide from different angles of views.*

We thank the reviewer for pointing out the need for updating the language referring to this study. We have removed both instances of the words "During the preparation of this manuscript" (in the Introduction and on line 595 in the Discussion) to better reflect the timeline of recent publications in this field.
